# Supplementary material for: Chemoselective Oxidation of Isoxazolidines with Ruthenium Tetroxide: A Successful Intertwining of Combined Theoretical and Experimental Data
Source: Molecules. 2022 Aug 24;27(17):5390. doi: 10.3390/molecules27175390 (PMC9478963; doi:10.3390/molecules27175390)
Supplement: Supplementary file 1 [file molecules-27-05390-s001.zip › molecules-1850817-supplementary.pdf]

Supporting Information

to

Chemoselective Oxidation of Isoxazolidines with Ruthenium Tetroxide: A Successful Intertwining of Combined Theoretical and Experimental Data

Laura Legnani [1], Salvatore V. Giofré [2], Daniela Iannazzo [3], Consuelo Celesti [3,4], Lucia Veltri [5] and Maria Assunta Chiacchio [6,*]

[1] Dipartimento di Biotecnologie e Bioscienze, Università di Milano-Bicocca, Piazza della Scienza 2, 20126 Milano, Italy
[2] Dipartimento di Scienze Chimiche, Biologiche, Farmaceutiche ed Ambientali, Università di Messina, Viale F. Stagno D'Alcontres, 98166 Messina, Italy
[3] Dipartimento di Ingegneria, Università di Messina, Contrada di Dio, 98166 Messina, Italy
[4] Dipartimento di Medicina Clinica e Sperimentale, Università di Messina, Via Consolare Valeria, 98125 Messina, Italy
[5] Dipartimento di Chimica e Tecnologie Chimiche, Università della Calabria, Via Pietro Bucci 12/C, 87036 Aracavacata di Rende, Italy
[6] Dipartimento di Scienze del Farmaco e della Salute, Università di Catania, Viale A. Doria 6, 95125 Catania, Italy
* Correspondence: ma.chiacchio@unict.it

Table of Contents

Figure S1. ^{1}H NMR spectrum of **2a** in $CDCl_3$, recorded at 25°C and 500 MHz……………………….S3

Figure S2. ^{13}C NMR spectrum of **2a** in $CDCl_3$, recorded at 25°C and 125 MHz………………………S3

Figure S3. ^{1}H NMR spectrum of **2b** in $CDCl_3$, recorded at 25°C and 500 MHz………………………..S4

Figure S4. ^{13}C NMR spectrum of **2b** in $CDCl_3$, recorded at 25°C and 125 MHz……………………….S4

Figure S5. ^{1}H NMR spectrum of **2c** in $CDCl_3$, recorded at 25°C and 500 MHz……………..................S5

Figure S6. ^{13}C NMR spectrum of **2c** in $CDCl_3$, recorded at 25°C and 125 MHz………………………..S5

Figure S7. ^{1}H NMR spectrum of **3a** in $CDCl_3$, recorded at 25°C and 500 MHz………….……………..S6

Figure S8. ^{13}C NMR spectrum of **3a** in $CDCl_3$, recorded at 25°C and 125 MHz……………..................S6

Figure S9. ^{1}H NMR spectrum of **3c** in $CDCl_3$, recorded at 25°C and 500 MHz…………………………S7

Figure S10. ^{13}C NMR spectrum of **3c** in $CDCl_3$, recorded at 25°C and 125 MHz………………………...S7

Figure S11. ^{1}H NMR spectrum of **6** in $CDCl_3$, recorded at 25°C and 500 MHz…………………………..S8

Figure S12. ^{13}C NMR spectrum of **6** in $CDCl_3$, recorded at 25°C and 125 MHz………………………....S8

Figure S13. ^{1}H NMR spectrum of **7** in $CDCl_3$, recorded at 25°C and 500 MHz……………....................S9

Figure S14. ^{13}C NMR spectrum of **7** in $CDCl_3$, recorded at 25°C and 125 MHz………………………....S9

Table S1. Free energies and imaginary frequencies for transition states to the oxidation reaction of **1a**....S10

Table S2. Free energies and imaginary frequencies for transition states to the oxidation reaction of **1b**....S11

Table S3. Free energies and imaginary frequencies for transition states to the oxidation reaction of **1b**…S12

Figure S15. NBO analysis of ion pair IP located along the reaction pathway of compounds **1a-c**……….S13

B3LYP / def2svp / emp=gd3bj / int=ultrafine / solvent =water cartesian coordinates…………...S14-S44

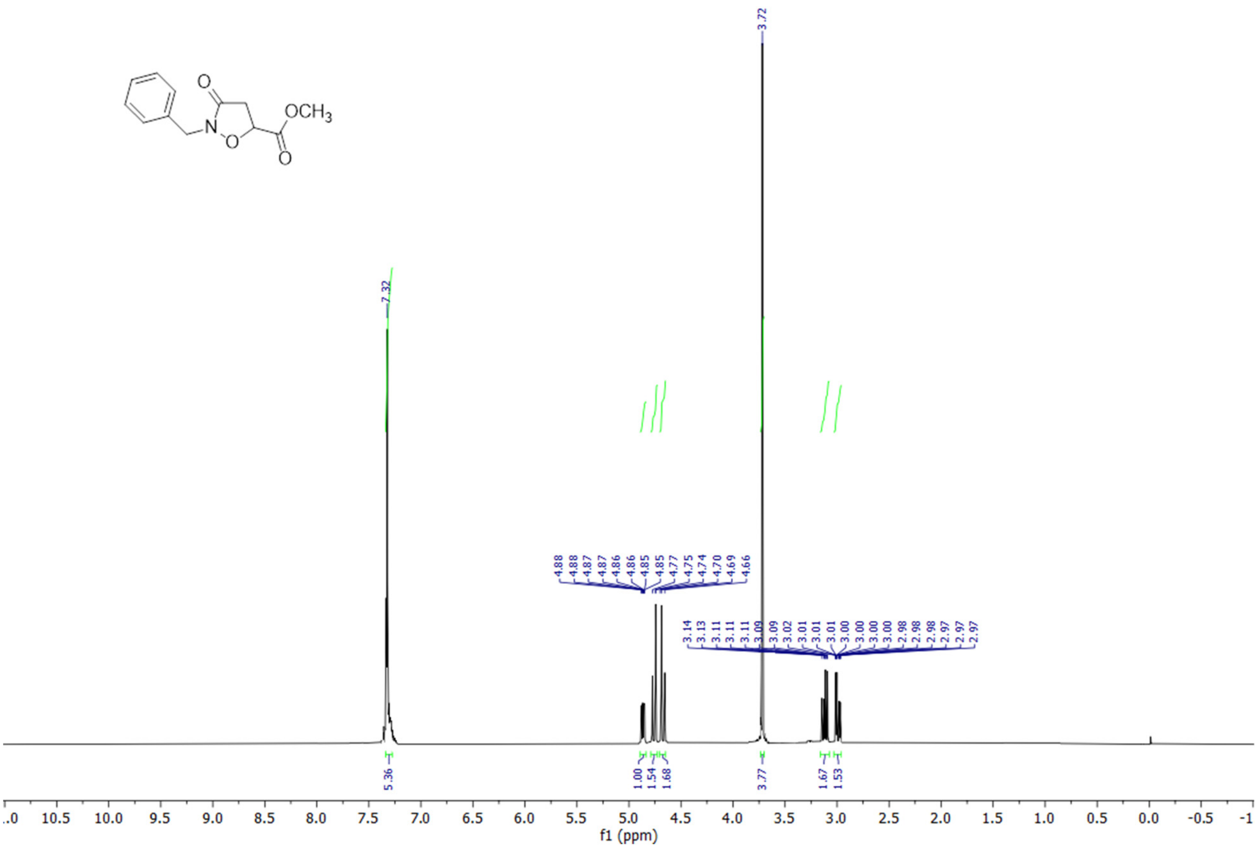

Figure S1. ^{1}H NMR spectrum of **2a** in $CDCl_3$, recorded at 25°C and 500 MHz

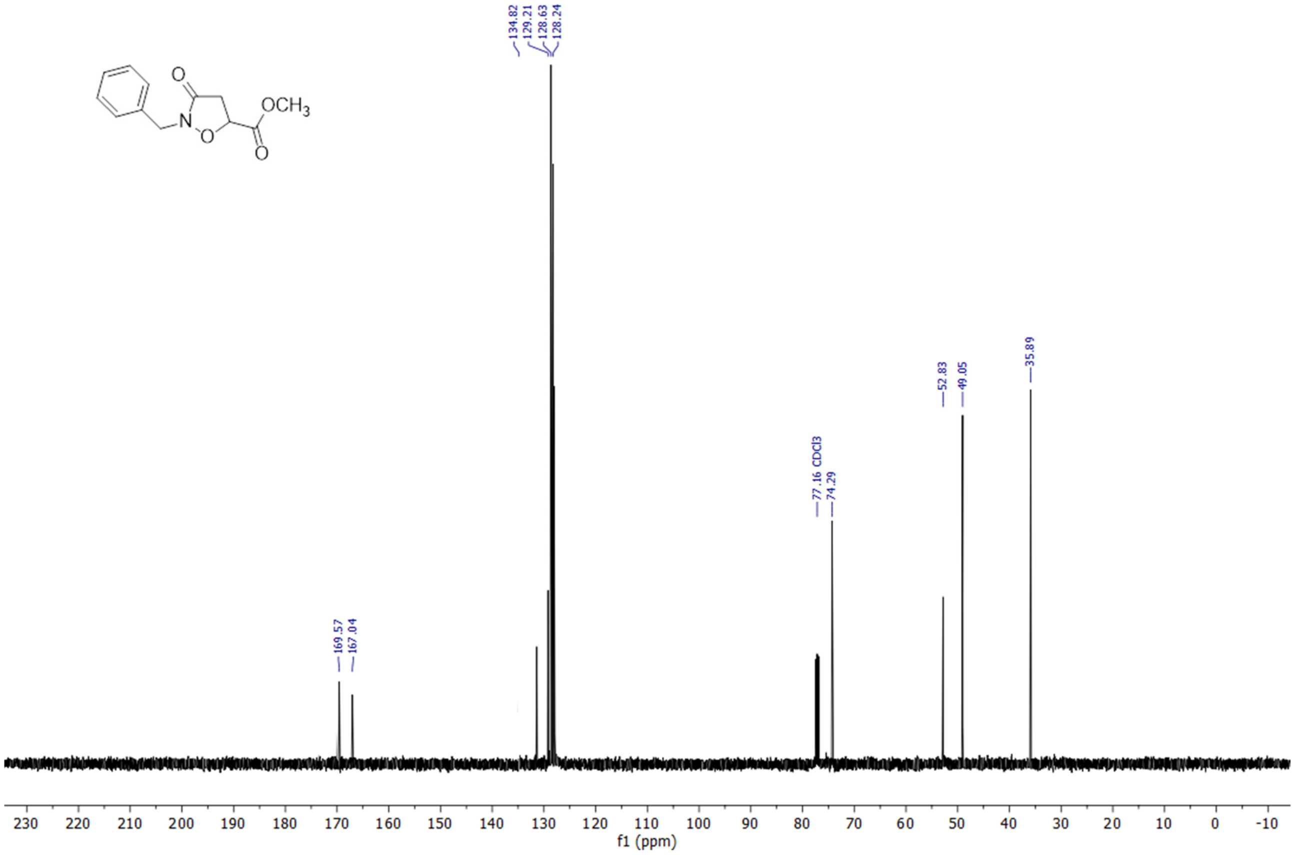

Figure S2. ^{13}C NMR spectrum of **2a** in $CDCl_3$, recorded at 25°C and 125 MHz

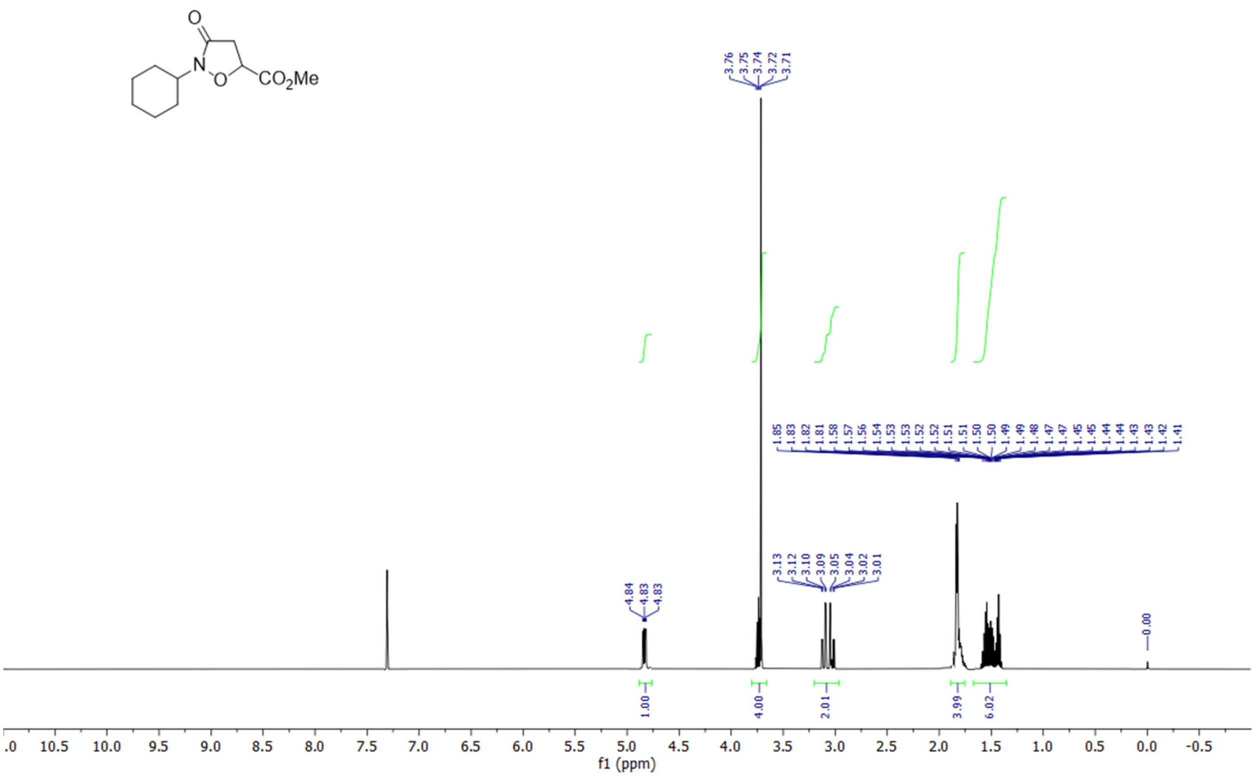

Figure S3. ^{1}H NMR spectrum of **2b** in $CDCl_3$, recorded at 25°C and 500 MHz

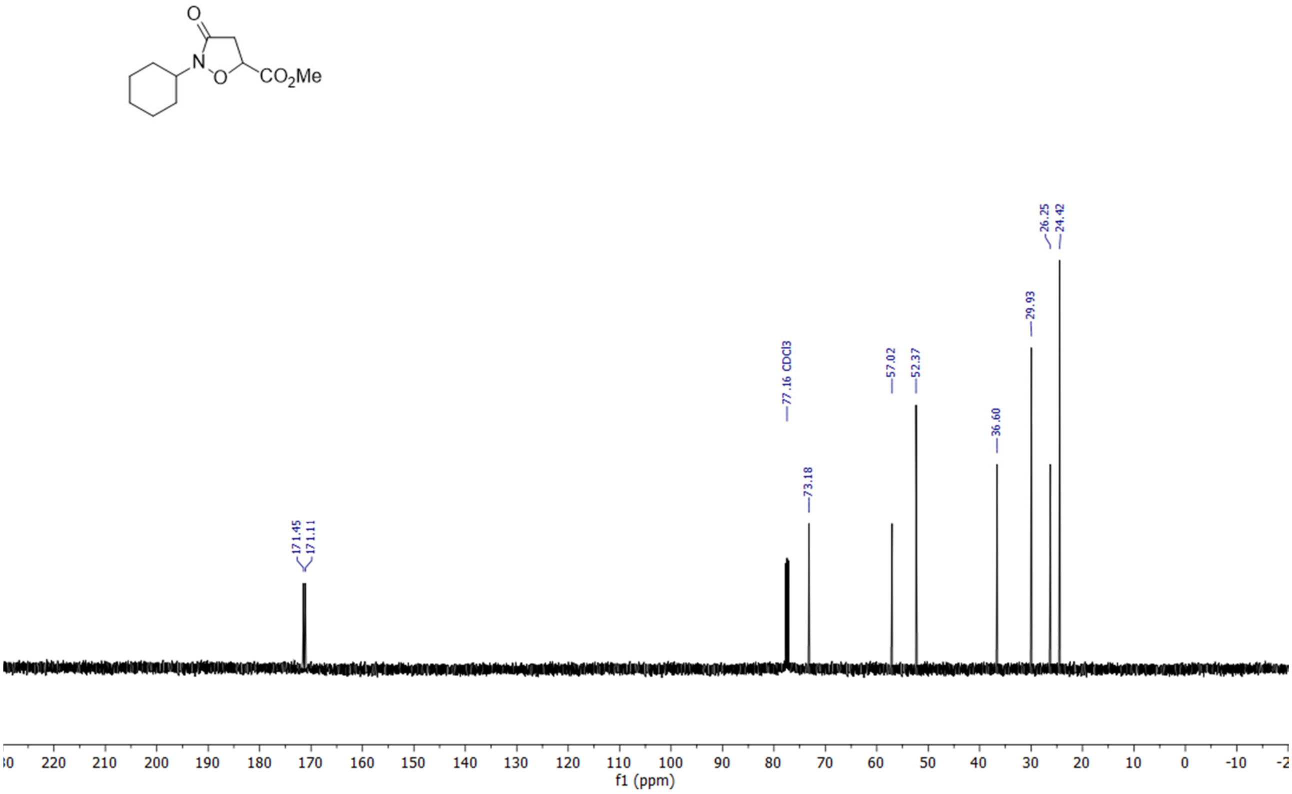

Figure S4. ^{13}C NMR spectrum of **2b** in $CDCl_3$, recorded at 25°C and 125 MHz

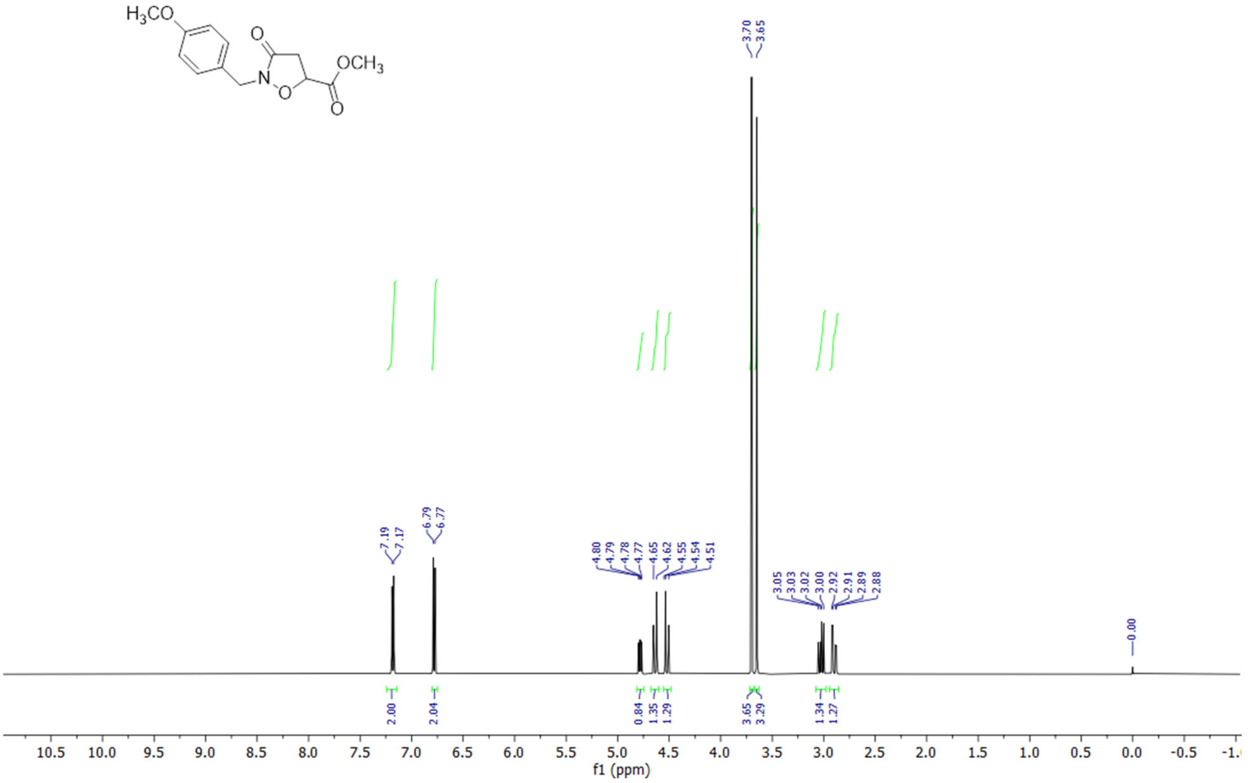

Figure S5. ^{1}H NMR spectrum of **2c** in $CDCl_3$, recorded at 25°C and 500 MHz

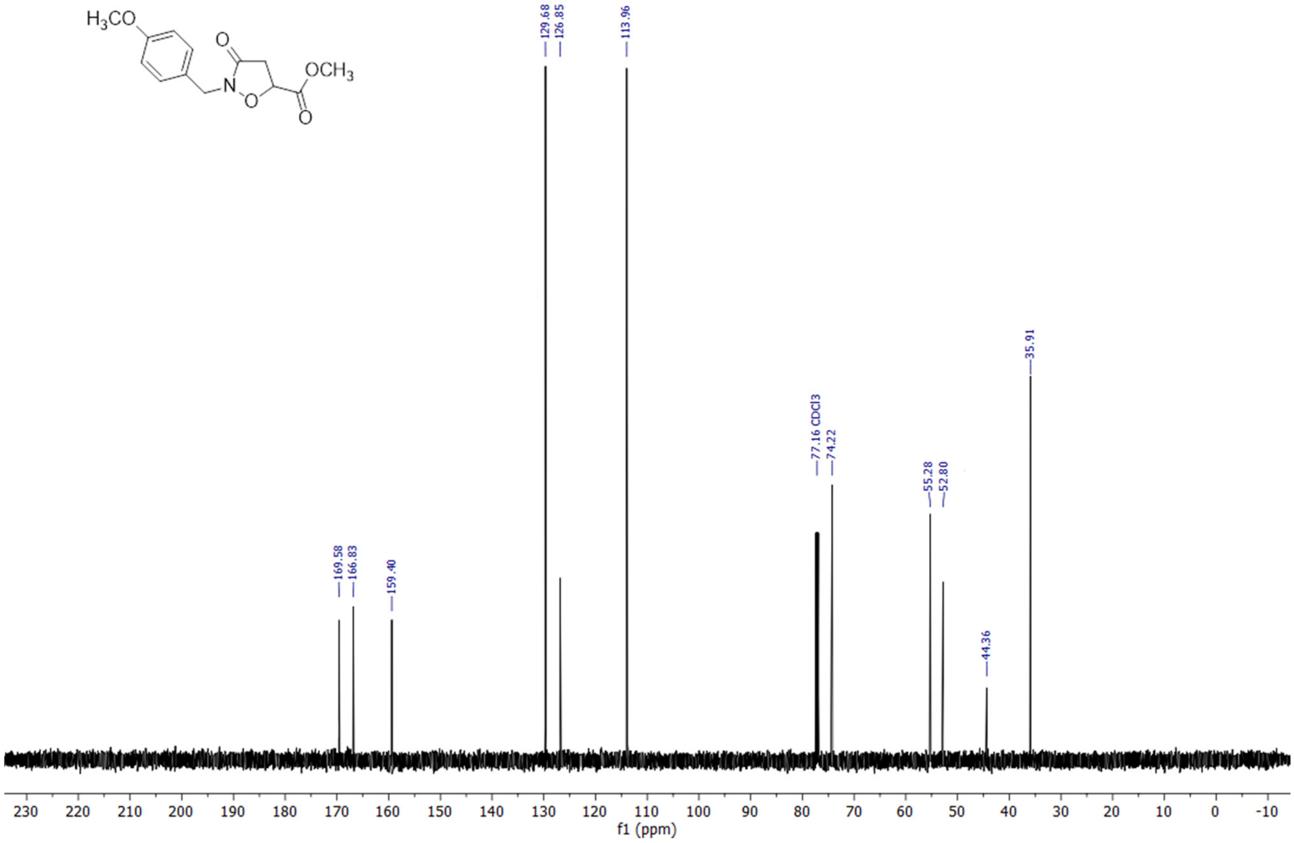

Figure S6. ^{13}C NMR spectrum of **2c** in $CDCl_3$, recorded at 25°C and 125 MHz

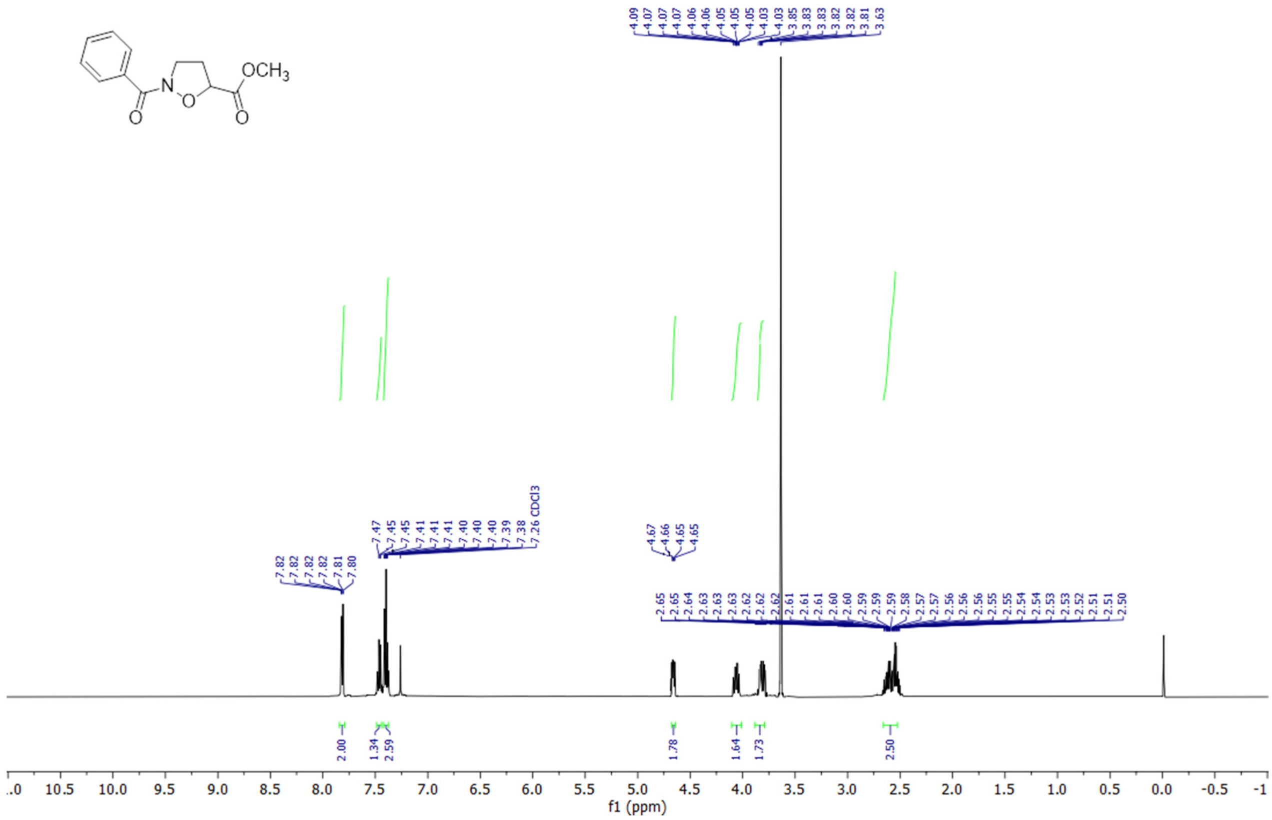

Figure S7. ^{1}H NMR spectrum of **3a** in $CDCl_3$, recorded at 25°C and 500 MHz

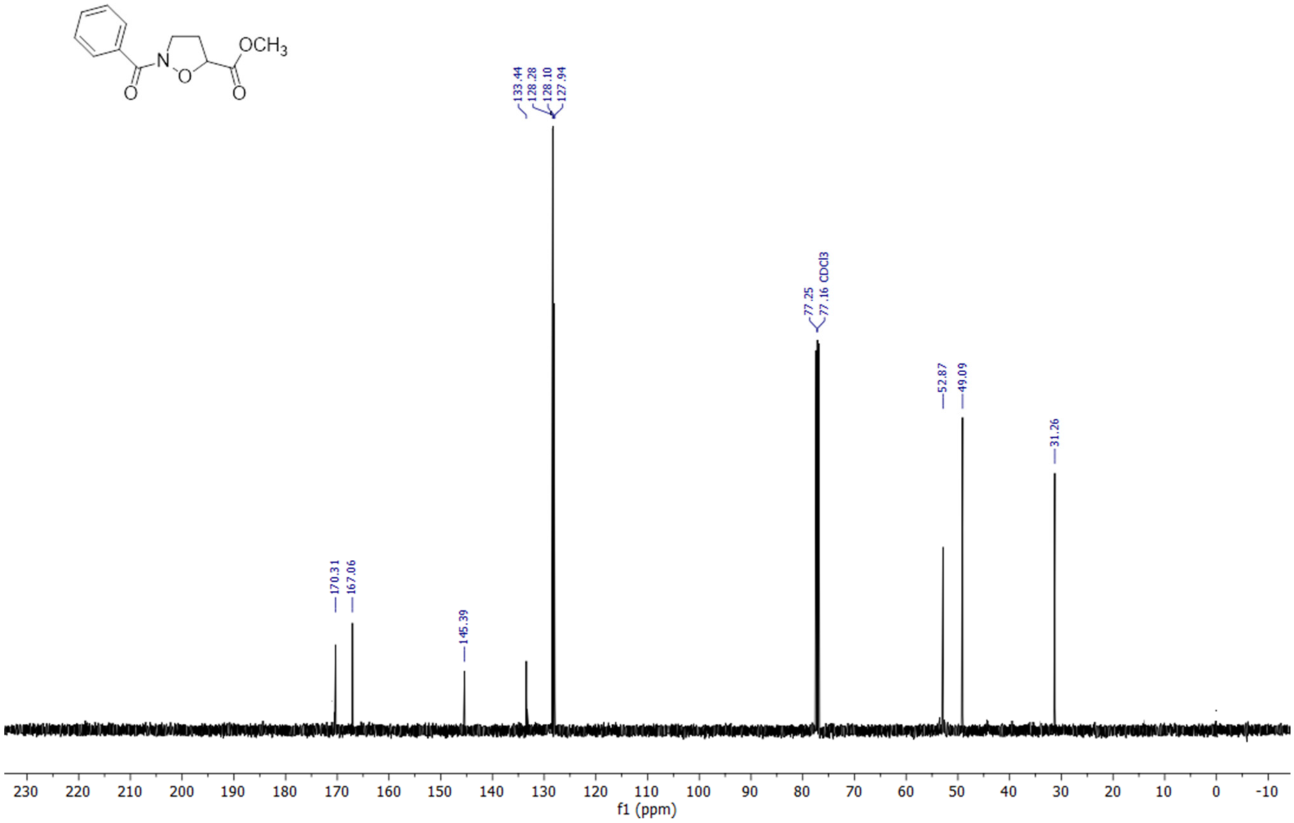

Figure S8. ^{13}C NMR spectrum of **3a** in $CDCl_3$, recorded at 25°C and 125 MHz

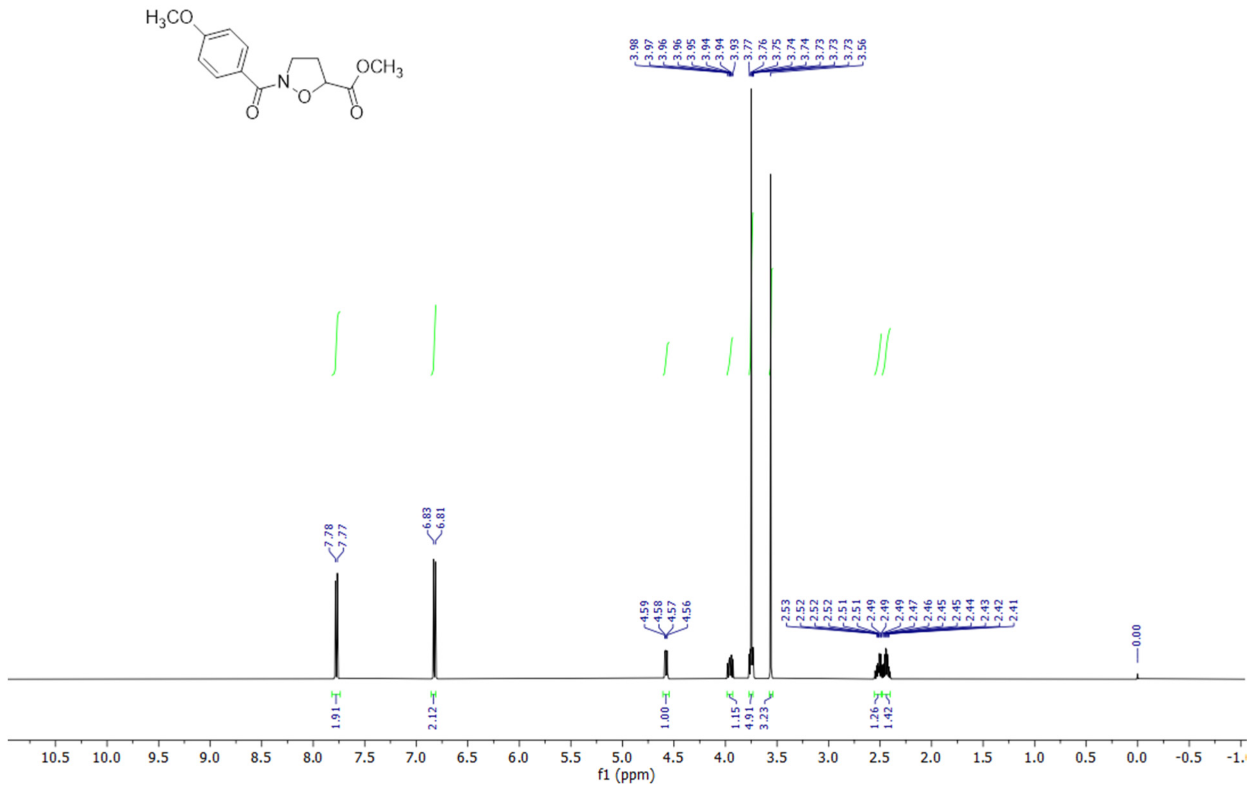

Figure S9. ^{1}H NMR spectrum of **3c** in $CDCl_3$, recorded at 25°C and 500 MHz

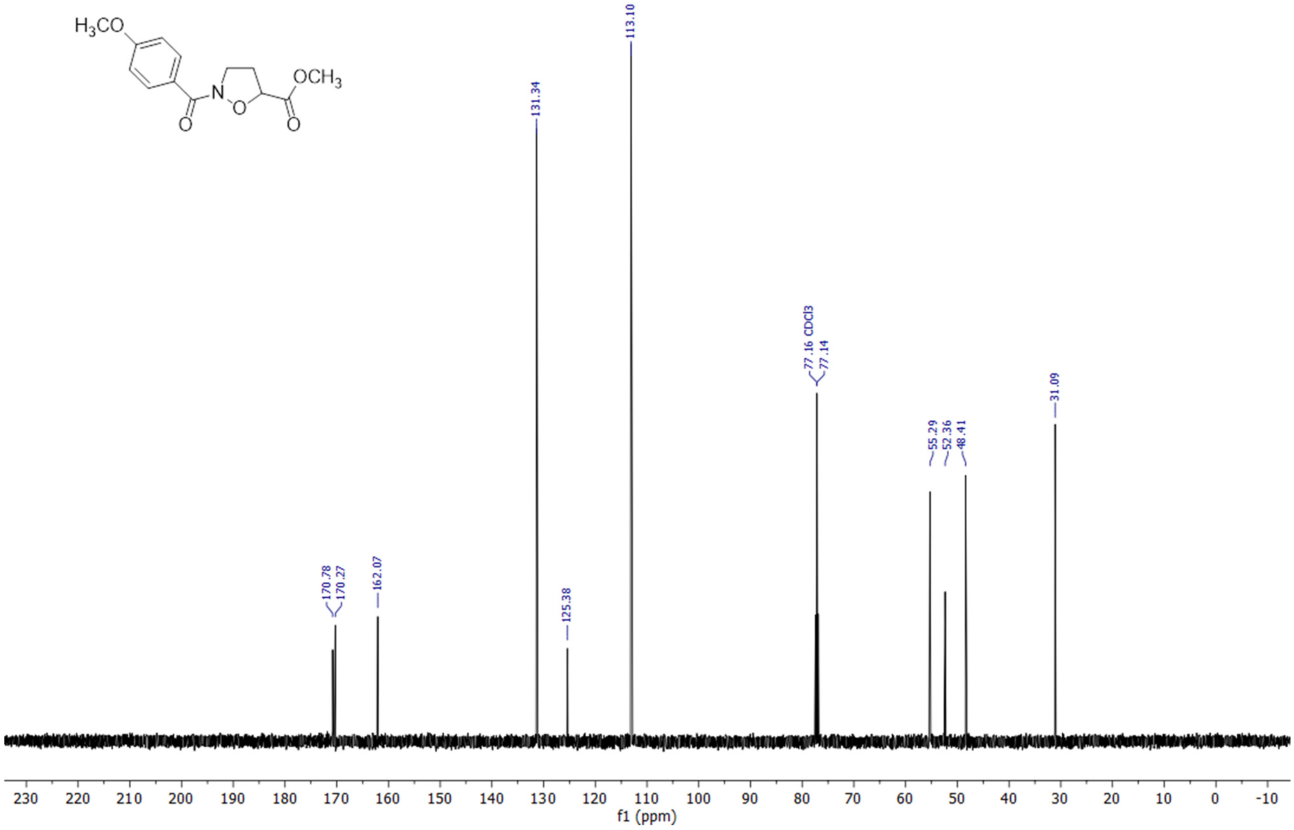

Figure S10. ^{13}C NMR spectrum of **3c** in $CDCl_3$, recorded at 25°C and 125 MHz

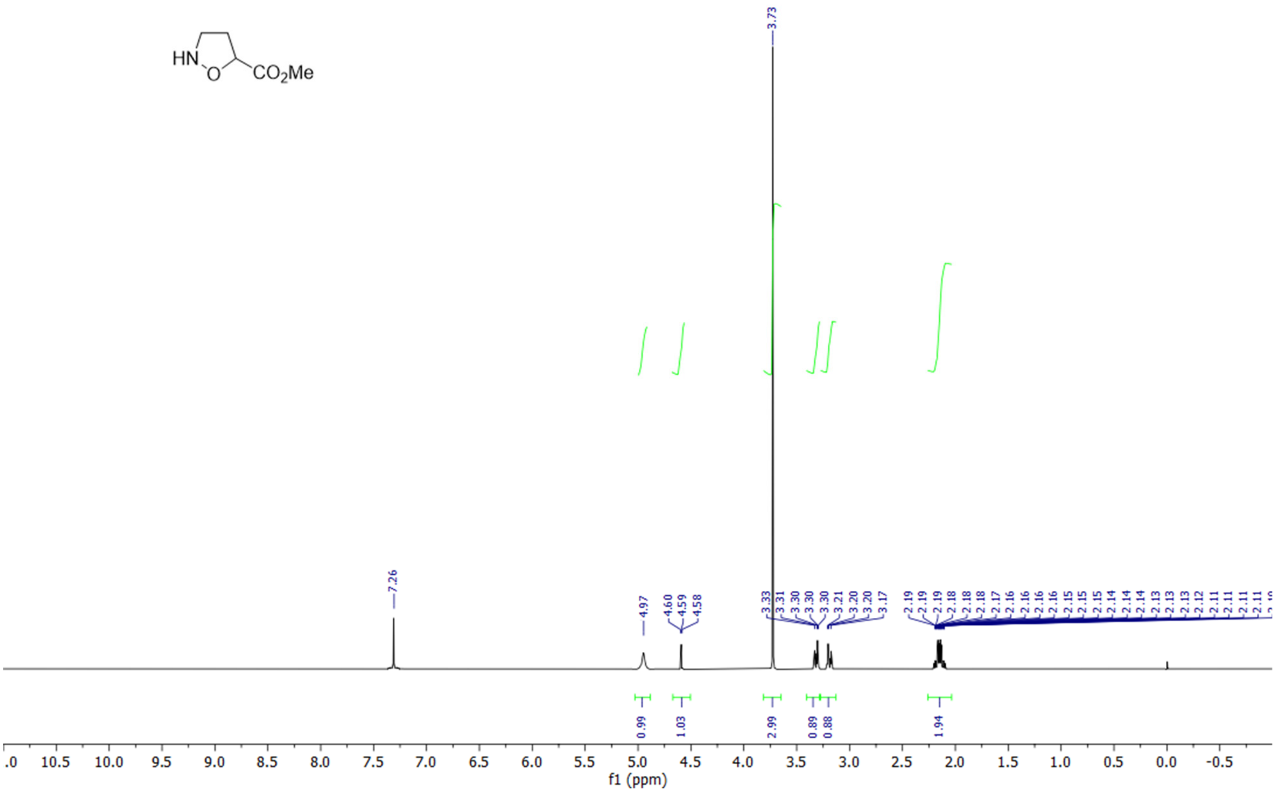

Figure S11. ^{1}H NMR spectrum of **6** in $CDCl_3$, recorded at 25°C and 500 MHz

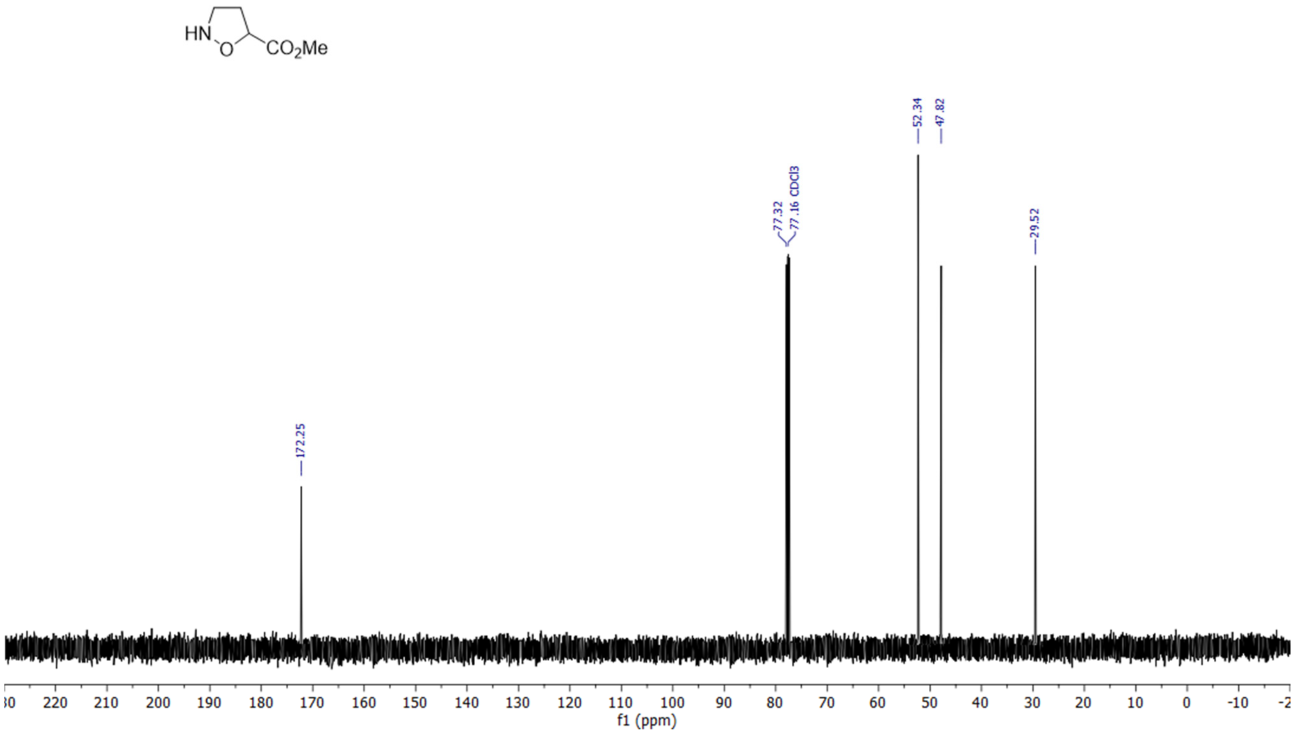

Figure S12. ^{13}C NMR spectrum of **6** in $CDCl_3$, recorded at 25°C and 125 MHz

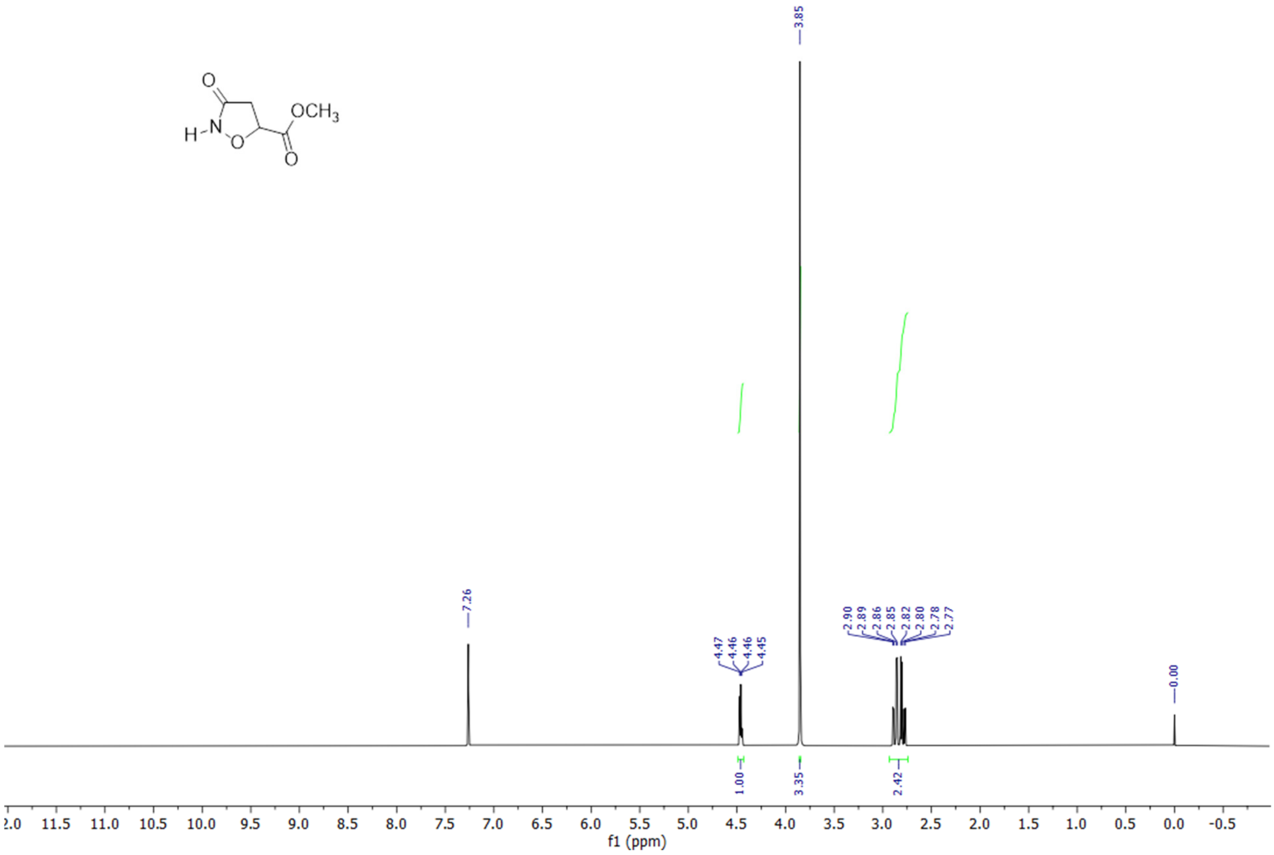

Figure S13. ^{1}H NMR spectrum of **7** in $CDCl_3$, recorded at 25°C and 500 MHz

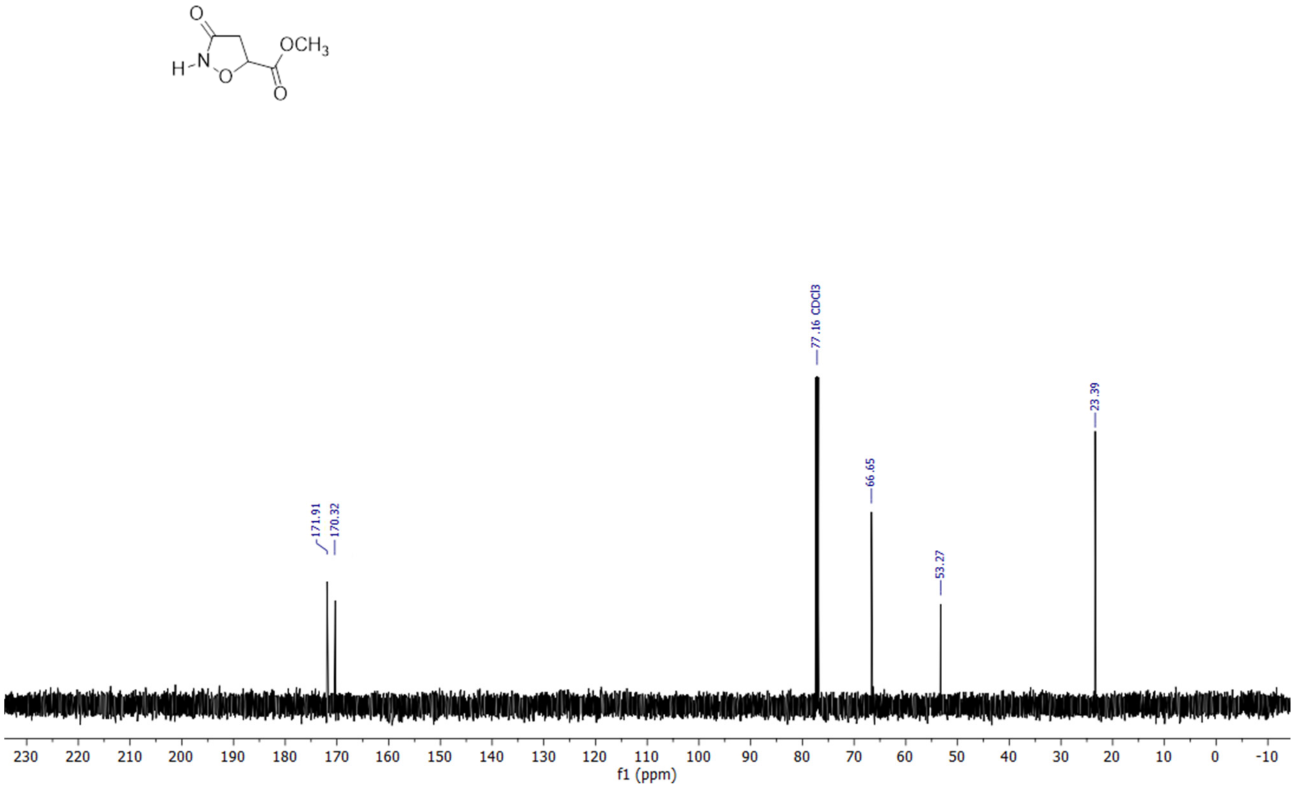

Figure S14. ^{13}C NMR spectrum of **7** in $CDCl_3$, recorded at 25°C and 125 MHz

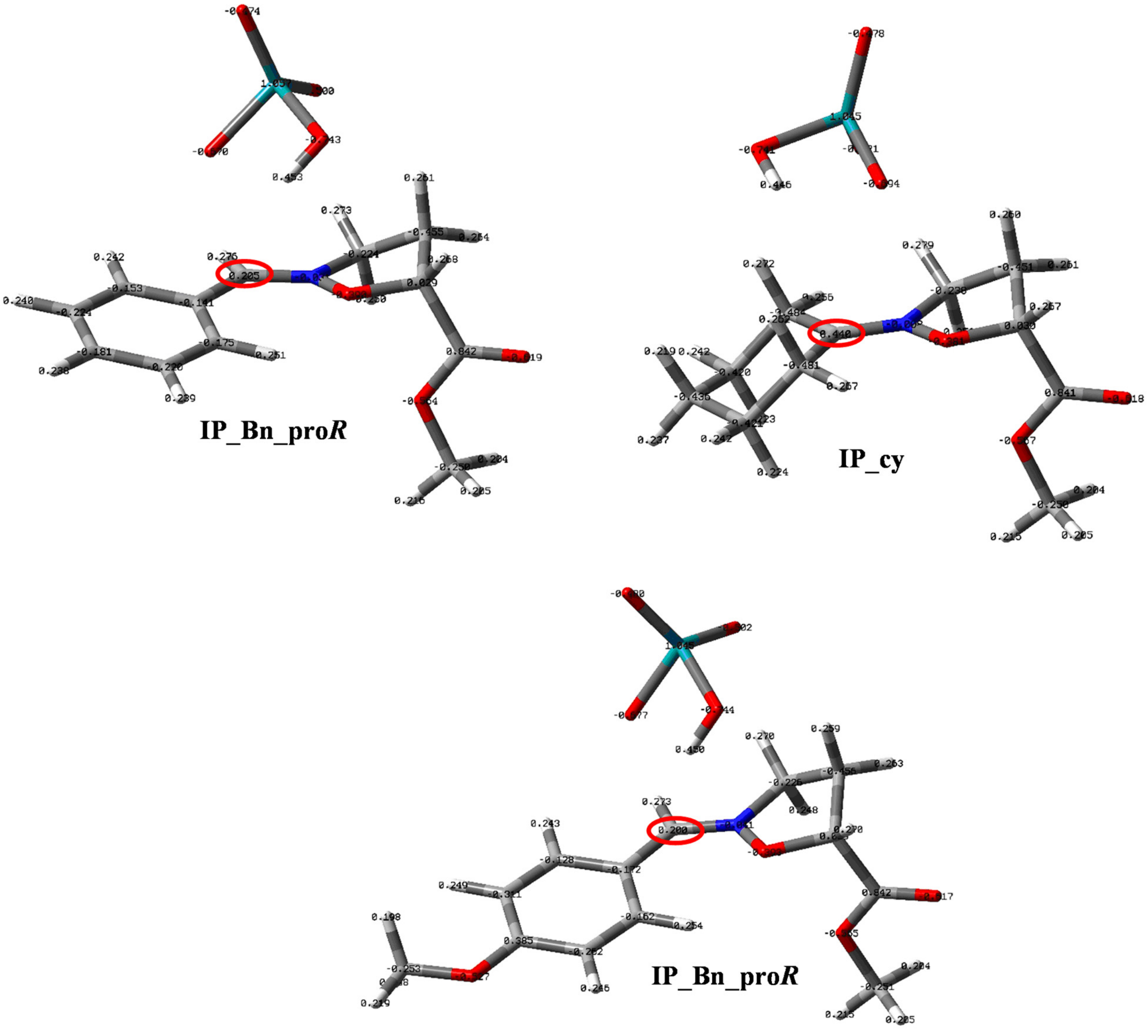

Figure S15. NBO analysis of ion pair IP located along the reaction pathway of compounds **1a-c.**

Table S1. B3LYP / def2svp / emp=gd3bj / int=ultrafine / solvent =water Energies. Free energies and imaginary frequencies for transition states related to the oxidation reaction of **1a**.

1a			
**Name**	**E(u.a.)**	**G (u.a.)**	**Imaginary frequency**
**RuO_4**	-395.512298	-395.528680	
**Isoxazoline_Bn**	-746.195798	-745.982836	
**TS1_endo_Bn**	-1141.707560	-1141.496466	-858.67
**P1_endo_Bn**	-1141.793782	-1141.574838	
**TS1_exo_proR_Bn**	-1141.709110	-1141.496277	-575.97
**TS1_exo_proS_Bn**	-1141.710093	-1141.496314	-828.61
**IP_exo_proR_Bn**	-1141.765381	-1141.549878	
**IPexo_proS_Bn**	-1141.761195	-1141.545329	
**TS_IPexo_proR_Bn**	-1141.764797	-1141.548537	-62.19
**TS_IPexo_proS_Bn**	-1141.759802	-1141.542039	-74.96
**P1_exo_proR_Bn**	-1141.795093	-1141.573633	
**P1_exo_proS_Bn**	-1141.792376	-1141.571844	
**TS2_endo_Bn**	-1141.769510	-1141.554286	-1103.01
**TSsec_exo_proS_Bn**	-1141.746553	-1141.532079	-1258.27
**TSsec_exo_proR_Bn**	-1141.740285	-1141.526535	-1459.19
**TS2_exo_proS_Bn**	-1141.766575	-1141.552351	-1167.05
**T2S_exo_proR_Bn**	-1141.750345	-1141.536255	-1374.90
**P2_exo_Bn**	-1141.831988	-1141.615800	
**P2_exo_sec_Bn**	-1141.803637	-1141.592976	
**P2_endo_Bn**	-1141.841442	-1141.622466	
**3-isoxazolidinone**	-820.176198	-819.985387	
**TS1b_ exo _proR_Bn**	-1215.684278	-1215.492033	-964.09
**P1b_exo_proR_Bn**	-1.215.771961	-1215.571169	
**TS1b_ exo _proS_Bn**	-1215.674424	-1215.483631	-1287.06
**P1b_ exo _proS_Bn**	-1215.766550	-1215.566653	
**TS2b_exo_proS_Bn**	-1215.741166	-1215.545673	-1360.87
**TS2b_exo_proR_Bn**	-1215.732996	-1215.540024	-1399.77

Table S2. B3LYP / def2svp / emp=gd3bj / int=ultrafine / solvent =water Energies. Free energies and imaginary frequencies for transition states related to the oxidation reaction of **1b**.

**1b**			
**Name**	**E(u.a.)**	**G (u.a.)**	**Imaginary frequency**
**Isoxazoline_cy**	-710.537491	-710.282032	
**TS1_cy_endo_$^4C_1$**	-1106.052503	-1105.798286	-819.56
**TS1_cy_endo_$^1C_4$**	-1106.051849	-1105.797818	-826.95
**TS1_cy_exo_$^4C_1$**	-1106.055466	-1105.800087	-446.14
**TS1_cy_exo_$^1C_4$**	-1106.055075	-1105.799707	-615.39
**IP_cy_exo_$^4C_1$**	-1106.112946	-1105.855239	
**TS_cy_IP_exo_$^4C_1$**	-1106.112055	-1105.854966	-33.22
**P1_cy_endo_$^4C_1$**	-1106.124796	-1105.862552	
**P1_cy_endo_$^1C_4$**	-1106.124502	-1105.862475	
**P1_cy_exo_$^4C_1$**	-1106.147602	-1105.884987	
**P1_cy_exo_$^1C_4$**	-1106.146465	-1105.883465	
**TS2_cy_exo_$^4C_1$**	-1106.104067	-1105.849463	
**TS2_cy_exo_$^1C_4$**	-1106.107527	-1105.850588	-1306.17
**TS2_cy_endo_$^4C_1$**	-1106.089542	-1105.832869	
**TS2_cy_endo_$^1C_4$**	-1106.090141	-1105.834009	-1404.09
**Psec_cy_exo_$^4C_1$**	-1106.141663	-1105.885345	
**Psec_cy_exo_$^1C_4$**	-1106.151572	-1105.891808	
**3-Isoxazolidinone_cy_$^4C_1$**	-784.520356	-784.284379	
**3-Isoxazolidinone_cy_$^1C_4$**	-784.522803	-784.289264	
**TS1b_exo_cy_$^4C_1$**	-1180.026049	-1179.790208	-1145.01
**TS1b_exo_cy_$^1C_4$**	-1180.024284	-1179.791177	-1145.81
**P1b_exo_cy_$^4C_1$**	-1180.123443	-1179.880154	
**P1b_exo_cy_$^1C_4$**	-1180.120996	-1179.878554	

Table S3. B3LYP / def2svp / emp=gd3bj / int=ultrafine / solvent =water Energies. Free energies and imaginary frequencies for transition states related to the oxidation reaction of **1c**.

	**c**		
**Name**	**E(u.a.)**	**G (u.a.)**	**Imaginary frequency**
**Isoxazoline_*p*OMe**	-860.641755	-860.400417	
**TS1_endo_*p*OMe**	-1256.154095	-1255.911728	-804.64
**P1_endo_*p*OMe**	-1256.232799	-1255.984506	
**TS2endo_*p*OMe**	-1256.216971	-1255.971354	-1060.73
**P2endo_*p*OMe**	-1256.288994	-1256.040418	
**TS1_exo_proR_*p*OMe**	-1256.157183	-1255.915425	-474.36
**TS1_exo_proS_*p*OMe**	-1256.157930	-1255.915095	-744.16
**IPexo_proR_*p*OMe**	-1256.215845	-1255.972016	
**IPexo_proS_*p*OMe**	-1256.211400	-1255.964751	
**TSIP_proR_*p*OMe**	-1256.214320	-1255.967994	-83.82
**TSIP_proS_*p*OMe**	-1256.210824	-1255.963270	-91.07
**P1_exo_proR_*p*OMe**	-1256.241821	-1255.991587	
**P1_exo_proS_*p*OMe**	-1256.238753	-1255.990313	
**TS2exo_proR_*p*OMe**	-1256.200284	-1255.955931	-1311.44
**TS2exo_proS_*p*OMe**	-1256.214345	-1255.970779	-1144.20
**P2exo_*p*OMe**	-1256.285624	-1256.039918	
**3-isoxazolidinone_*p*OMe**	-934.622225	-934.401371	
**TS1b_exo_proS_*p*OMe**	-1330.124556	-1329.904287	-1179.86
**P1b_ exo _proS_*p*OMe**	-1330.213099	-1329.982991	
**TS1b_ exo_proR_*p*OMe**	-1330.133379	-1329.911141	-841.49
**P1b_ exo _proR_*p*OMe**	-1330.218318	-1329.986391	
**TS2b_ exo_proR_*p*OMe**	-1330.182615	-1329.960641	-1383.12
**TS2b_ exo _proS_*p*OMe**	-1330.188813	-1329.965780	-1236.94
**1′_isoxazolidinone_*p*OMe**	-934.625484	-934.402748	
**TS1b_endo_*p*OMe**	-1330.128214	-1329.905993	-1229.59
**P1b_endo_*p*OMe**	-1330.212221	-1329.982331	
**TS2b_endo_*p*OMe**	-1330.185838	-1329.961252	-1468.88

B3LYP / def2svp / emp=gd3bj / int=ultrafine / solvent =water cartesian coordinates

RuO_4

O -1.27053900 0.62231000 0.91425100
O -0.53457700 -1.38333500 -0.79905800
O 0.49131900 1.15889200 -1.10757300
O 1.30216900 -0.39796300 0.98188800
Ru 0.00211400 0.00001700 0.00190800

Isoxazoline_Bn

C -2.87153400 -1.52724000 -0.01814600
H -3.52732000 -1.06899500 -0.76835000
H -3.47058500 -2.20897000 0.59838900
C -1.65934300 -2.24949500 -0.63631300
H -1.47335100 -3.21884300 -0.13328300
N -0.56589200 -1.30463800 -0.39738400
H -1.75866100 -2.42367000 -1.71679600
C -2.18701100 -0.44019600 0.84283000
O -0.84385000 -0.85186800 0.95696200
H -2.59551200 -0.37652200 1.86354900
C 0.76718000 -1.86185200 -0.39569400
H 0.88609800 -2.35944600 -1.37359500
H 0.87429800 -2.64788200 0.37981400
C 1.83954600 -0.80745400 -0.22302200
C 3.04371000 -1.12451300 0.41923600
C 1.66094700 0.48976600 -0.72535600
C 4.05597900 -0.16823900 0.55158700
H 3.18977500 -2.12975800 0.82467100
C 2.66735300 1.44852100 -0.58836400
H 0.71500600 0.74123800 -1.20557600
C 3.87027800 1.12295100 0.04876700
H 4.98898900 -0.43150100 1.05612900
H 2.51105600 2.45736900 -0.97921900
H 4.65692000 1.87371500 0.15676100
C -2.31036800 0.95241700 0.21057200
O -3.27129600 1.32508300 -0.42449900
O -1.25630300 1.71961900 0.48387600
C -1.26162900 3.04171500 -0.06297700
H -2.10531800 3.62390000 0.33556600
H -0.30967100 3.49658700 0.23349900
H -1.33954300 3.00428700 -1.15929700

TS1_endo_Bn

C 0.38410600 2.10779700 0.72947800
H -0.00056000 2.91005800 1.37258200
H 1.41224600 2.35647200 0.44276900
C 0.30907200 0.74016100 1.39989500
H 1.44139600 0.16461700 1.31380500
O 2.68028100 -0.27635000 1.43982800
O 4.56923300 0.81433700 -0.33002200
O 3.72811000 -2.00531600 -0.55597000
O 1.95953300 0.09331300 -1.00839900
Ru 3.39395100 -0.39201400 -0.16433700
N -0.67348400 0.04204900 0.71958000
H 0.18979200 0.69471400 2.49194100
C -0.53105200 1.90243900 -0.49765500
O -0.74335500 0.49234300 -0.58517900
H -0.05698100 2.20450300 -1.44119000
C -0.91601000 -1.37726500 0.82629900
H -0.72415100 -1.63768900 1.87852800
H -0.17010400 -1.91012700 0.20811000
C -2.32542100 -1.74347300 0.41894500
C -2.56138100 -2.84550300 -0.41034300
C -3.41249100 -0.98913800 0.88378400
C -3.86817100 -3.19680300 -0.76599200
H -1.71787900 -3.43125200 -0.78491000
C -4.71614200 -1.33412800 0.52419900
H -3.23046600 -0.11882200 1.51768800
C -4.94772100 -2.44128000 -0.30085700
H -4.04074900 -4.05884800 -1.41459200
H -5.55557400 -0.73712900 0.88869300
H -5.96832000 -2.71119800 -0.58225300
C -1.85149300 2.66614400 -0.35260200
O -1.89283400 3.82067000 0.00343400
O -2.90734400 1.92934300 -0.67116600
C -4.19499200 2.54685900 -0.54001500
H -4.28986800 3.38874300 -1.24045800
H -4.92711900 1.76651800 -0.77446800
H -4.33993600 2.91212700 0.48651900

P1_endo_Bn

C 0.67648900 1.82099700 -0.01759700

H 0.63405400 2.58524600 0.76683800
H 1.57692500 1.98627900 -0.62071900
C 0.66788900 0.39372500 0.56097200
H 2.56130300 -2.12561000 -1.04244400
O 3.49561400 -1.89003400 -0.87205700
O 3.81759000 -0.62546500 1.69659500
O 3.93052900 0.96248000 -0.94199900
O 1.60432800 -0.47522900 -0.08365200
Ru 3.46238700 -0.28130800 0.08914700
N -0.67020000 -0.04983500 0.26765900
H 0.83654600 0.34911900 1.64897400
C -0.60191000 1.83241100 -0.88055700
O -0.93390100 0.47081100 -1.05450300
H -0.44346400 2.26005900 -1.88202400
C -0.99611000 -1.46070300 0.29467600
H -0.60668400 -1.83934300 1.25464100
H -0.47703500 -2.01366000 -0.50700900
C -2.49112800 -1.68119100 0.20673100
C -3.00954900 -2.71959800 -0.57641200
C -3.37827900 -0.85873800 0.91666300
C -4.38971400 -2.93913300 -0.64736200
H -2.32704600 -3.35928700 -1.14271600
C -4.75611100 -1.07114300 0.84332400
H -2.97740600 -0.03813600 1.51389200
C -5.26724500 -2.11376400 0.06097000
H -4.77920700 -3.75203600 -1.26513500
H -5.43585600 -0.41831600 1.39675200
H -6.34582500 -2.27848700 0.00147300
C -1.73756300 2.61352100 -0.20528200
O -1.56440100 3.57989000 0.50218600
O -2.93173500 2.12392900 -0.52600500
C -4.07102800 2.76115400 0.06123600
H -4.14040400 3.80860100 -0.26635100
H -4.94443200 2.19399100 -0.27925700
H -4.00371500 2.73171300 1.15830300

TS1_exo_proR_Bn

O -0.81541600 -0.75762800 -0.71132300
O -3.56111600 -1.17859300 -1.19103300
O -1.88821400 -3.21467900 0.11639300
O -2.37756000 -0.73961900 1.29537800
Ru -2.25751100 -1.59335500 -0.19879800
C 1.77345800 -0.45871400 1.92660300
C 2.23870700 -0.73237800 -0.39997800
C 1.90641300 -1.55442300 0.86182300
H 1.07634000 -0.70620400 2.73565000
H 2.75370200 -0.20337100 2.35522500
H 1.65116400 -1.05815500 -1.26876600
H 0.96027400 -2.09150900 0.72119200
H 2.68784700 -2.28361300 1.11099400
N 1.28039100 0.68793000 1.17536200
O 1.88097000 0.63562300 -0.08960700
C -0.09671700 0.97289900 1.13355100
H -0.55937800 0.12763700 0.36927900
H -0.53028800 0.81453700 2.12511900
C -0.55213600 2.24313500 0.49103900
C -1.93591400 2.49602200 0.44813600
C 0.33365000 3.17163700 -0.07932300
C -2.42258100 3.66330900 -0.13828300
H -2.62348200 1.76085000 0.87248700
C -0.15931200 4.33934800 -0.66609800
H 1.40625300 2.98167900 -0.05656100
C -1.53499300 4.58915300 -0.69980200
H -3.49847500 3.85060100 -0.16251100
H 0.53802500 5.06004600 -1.09965600
H -1.91556400 5.50152000 -1.16469600
C 3.71316800 -0.80046300 -0.76605100
O 4.13806900 -1.43596200 -1.70178200
O 4.47128800 -0.12605100 0.09819600
C 5.88812400 -0.16596100 -0.12005700
H 6.13473800 0.24596400 -1.10897800
H 6.33485500 0.44615900 0.67094600
H 6.25636900 -1.20014400 -0.05926400

TS1_exo_proS_Bn

O 0.75288800 1.33864000 -1.43876500
O 1.66582600 3.68353300 -0.16254400
O -1.17528700 2.89914800 -0.11549000
O 0.76389200 1.49629100 1.26564700
Ru 0.46532500 2.48912300 -0.13528800
C -0.24851700 -1.23758700 1.31902400
C -2.28022900 -0.50731800 0.36910900
C -1.73083800 -1.13144500 1.65829600
H 0.28087000 -0.27762700 1.52534400
H 0.28228100 -2.06098500 1.81571300
H -2.15802900 0.58999100 0.36127800
H -1.91679900 -0.49909000 2.53430600
H -2.17735400 -2.12251500 1.82150600

N -0.24912200 -1.43232700 -0.11470400
O -1.46991200 -1.07891300 -0.66955700
C 0.83781400 -1.20195100 -0.94525700
H 0.92098200 0.04954000 -1.09248300
C 2.15788200 -1.71975800 -0.46054800
C 2.91434900 -1.00700500 0.48331200
C 2.65373300 -2.93228700 -0.96689100
C 4.13850900 -1.51157000 0.92816600
H 2.53947600 -0.05314100 0.86039900
C 3.87776700 -3.43410700 -0.51989700
H 2.07230500 -3.48724100 -1.70688000
C 4.62175200 -2.72515600 0.42937500
H 4.72043700 -0.94976400 1.66222900
H 4.25202100 -4.38156900 -0.91428800
H 5.58137000 -3.11627000 0.77527100
H 0.58599700 -1.48501000 -1.97818600
C -3.74763500 -0.81589900 0.12811700
O -4.62598900 -0.03305100 0.40033900
O -3.93633000 -2.04447900 -0.34167100
C -5.29786800 -2.45665200 -0.53298100
H -5.80066600 -1.79442100 -1.25182100
H -5.25259000 -3.47975900 -0.92149800
H -5.84175400 -2.43220000 0.42207300

IP_exo_proR_Bn

O -1.23137300 -0.93649600 -1.36758800
O -4.10938500 -1.05605900 -0.85604000
O -2.07924700 -2.20569400 1.15344200
O -2.13596400 0.49452000 0.45194100
Ru -2.59910500 -1.16197200 -0.08873700
C 1.01104800 -1.35719100 1.34166900
C 2.07239000 -1.38300000 -0.78087200
C 1.28290900 -2.29246800 0.16957700
H 0.06461800 -1.56864800 1.85061100
H 1.84453600 -1.31608700 2.05738600
H 1.89110300 -1.61371400 -1.83749500
H 0.34161100 -2.58773000 -0.30807600
H 1.84942300 -3.18395300 0.46267100
N 0.91348000 -0.04819400 0.67163800
O 1.54790500 -0.06297400 -0.55207000
C 0.22078300 0.98460000 1.03914100
H -0.96710300 -0.00514400 -1.20174800
H -0.30344000 0.83346900 1.98144300
C 0.18109600 2.30430100 0.43964000
C -0.75770700 3.20349900 0.99182200
C 1.02267100 2.74018200 -0.60629900
C -0.86629500 4.49956300 0.49995000
H -1.41227100 2.86284700 1.79528900
C 0.91135700 4.04412000 -1.08599300
H 1.76406300 2.06709200 -1.02843000
C -0.03181600 4.92250800 -0.54207900
H -1.60045400 5.18481600 0.92812100
H 1.56781600 4.37753100 -1.89223900
H -0.11454000 5.94101600 -0.92799900
C 3.57458500 -1.43865000 -0.52124100
O 4.28258000 -2.28229800 -1.01460100
O 3.97396500 -0.50297300 0.33477100
C 5.36697500 -0.50054700 0.68609100
H 5.98354200 -0.34621600 -0.21038600
H 5.49916200 0.32796300 1.38991500
H 5.64237000 -1.45480800 1.15660000

IP_exo_proS_Bn

O 2.10473600 1.05344000 -1.20888300
O 2.08872000 3.76646800 -0.05066000
O -0.50703600 2.18994000 -0.45629900
O 1.56308200 1.27846400 1.18623200
Ru 1.17061300 2.35148500 -0.21357400
C -0.65592900 -0.64803100 1.42519500
C -2.59690200 0.13916800 0.31147000
C -2.12930200 -0.36090300 1.68104100
H 0.00752800 0.23210500 1.54611000
H -0.25475700 -1.49101400 2.00107800
H -2.38688800 1.20786000 0.17349400
H -2.27307300 0.39505000 2.46153500
H -2.66643800 -1.27861500 1.95925900
N -0.64788900 -1.01534800 -0.00128000
O -1.79185800 -0.59702300 -0.63540900
C 0.20952500 -1.66298800 -0.72017200
H 2.39925400 0.46004900 -0.48175000
C 1.46750300 -2.23659800 -0.29050600
C 2.21518400 -1.79072200 0.82266400
C 1.95986000 -3.30246600 -1.07697300
C 3.40835300 -2.43119800 1.15016300
H 1.91741700 -0.89402400 1.36529500
C 3.14392500 -3.94456700 -0.72793200

H 1.39743000 -3.63180900 -1.95302900
C 3.86733700 -3.51281700 0.38978200
H 3.99266000 -2.07424600 2.00048500
H 3.50909500 -4.77712400 -1.33198600
H 4.80160600 -4.01031600 0.65902900
H -0.11557800 -1.82187800 -1.75206400
C -4.07102400 -0.12331000 0.05882000
O -4.91706900 0.70207300 0.30470200
O -4.30316900 -1.35329600 -0.38423500
C -5.67782300 -1.71636000 -0.58526000
H -6.14688000 -1.04565200 -1.31873800
H -5.66663600 -2.74574400 -0.95931700
H -6.22996600 -1.65831600 0.36348600

TS_IPexo_proR_Bn

O -1.32008800 -0.89004300 -1.42306200
O -4.15620100 -0.62745100 -0.73702300
O -2.23864600 -2.34133200 0.95612000
O -1.96528200 0.41781900 0.67009800
Ru -2.64772600 -1.06597000 -0.09355000
C 0.98410400 -1.45565300 1.29031300
C 2.06424200 -1.46079100 -0.81810700
C 1.24929700 -2.37601900 0.10260400
H 0.01944300 -1.64638200 1.77353600
H 1.79722100 -1.46813600 2.02999000
H 1.93668700 -1.69799600 -1.88130000
H 0.30710800 -2.64182100 -0.39224000
H 1.79434800 -3.28494900 0.38198400
N 0.94735400 -0.12646100 0.65263700
O 1.49408100 -0.15987100 -0.62077700
C 0.27465400 0.91556500 1.04118500
H -0.87176700 -0.04673000 -1.21088800
H -0.18143600 0.78865500 2.02064100
C 0.26056500 2.24216700 0.44459900
C -0.67957400 3.15204700 0.97470800
C 1.13542200 2.66938100 -0.57553700
C -0.76019700 4.44974500 0.48106900
H -1.35962900 2.81663400 1.75884000
C 1.05531400 3.97671900 -1.05446900
H 1.88087700 1.98745200 -0.97661900
C 0.10761700 4.86519600 -0.53661500
H -1.49848700 5.14248400 0.88991000
H 1.73980800 4.30369100 -1.83985100
H 0.04731800 5.88521600 -0.92274200
C 3.55501900 -1.48662600 -0.48744700
O 4.28216300 -2.37334000 -0.86364100
O 3.91916700 -0.46752500 0.28478800
C 5.29411700 -0.42521200 0.69868100
H 5.95249700 -0.35432200 -0.17826100
H 5.39258000 0.46747000 1.32544400
H 5.54685800 -1.32851600 1.27135800

TS_IPexo_proS_Bn

O -0.96652100 1.50663800 -1.24609600
O 1.44072900 2.87140100 -0.28828800
O -1.25694000 2.98171900 1.21313400
O 0.31820200 0.62973500 0.85867100
Ru -0.07036500 2.27904900 0.22708300
C 0.10983700 -2.87576300 0.57888200
C -1.88208000 -1.57841300 0.45825900
C -1.35959300 -2.88500200 1.02884200
H 0.80345000 -2.57578800 1.37475400
H 0.42584000 -3.83854300 0.15826800
H -1.59931100 -0.71350100 1.08218100
H -1.45209000 -2.92301300 2.12073800
H -1.90637700 -3.73224700 0.59625100
N 0.13996200 -1.83297000 -0.46737000
O -1.15572400 -1.45216600 -0.79833900
C 1.05866700 -0.94558200 -0.73977200
H -1.12399400 0.56006400 -1.05559100
C 2.47327900 -1.05849300 -0.44770200
C 3.12503400 -2.26260000 -0.10835000
C 3.22165500 0.13589000 -0.53896500
C 4.49472500 -2.26306900 0.14548600
H 2.58291700 -3.20625200 -0.08138500
C 4.58590700 0.12682000 -0.26224800
H 2.71839000 1.07083000 -0.79477100
C 5.22487300 -1.07088200 0.07805500
H 4.99718300 -3.20044400 0.39131100
H 5.15637300 1.05596900 -0.31961600
H 0.73036100 -0.13597100 -1.38663200
C -3.36416900 -1.52482700 0.13658400
O -4.16757600 -2.36280200 0.46465200
O -3.64724000 -0.40920300 -0.53016100
C -5.01755100 -0.20265500 -0.90660100
H -5.64867100 -0.12541900 -0.00997200

H -5.03663800 0.73567300 -1.47078700
H -5.37201600 -1.03554800 -1.52977200
H 6.29797300 -1.07831300 0.28153800

P1_exo_proR_Bn

O -0.78568100 -0.54115000 -1.55398000
O -3.05064700 -1.72980500 -0.34913300
O -0.21154600 -2.96304200 -0.28469300
O -0.94335500 -0.72654600 1.28522600
Ru -1.37368000 -1.74714100 -0.24880500
C 1.80845600 0.10620300 2.05276900
C 2.17823400 -0.52781500 -0.19955300
C 2.19753500 -1.11578800 1.21820400
H 1.32165400 -0.15547700 3.00025600
H 2.69027100 0.72803600 2.26389400
H 1.82772500 -1.25474800 -0.94490700
H 1.44870000 -1.91388300 1.29552900
H 3.17964900 -1.52526100 1.48852600
N 0.88267800 0.87072400 1.21458100
O 1.24701600 0.56506000 -0.15666200
C -0.52609000 0.64134100 1.40184000
H -0.21659800 0.14959800 -1.14361300
H -0.68968200 0.84631800 2.47451800
C -1.39652800 1.59954500 0.60596700
C -2.78690000 1.41011900 0.60717600
C -0.85663100 2.70109800 -0.07114100
C -3.62178200 2.29397400 -0.07864800
H -3.21755000 0.56525500 1.14659900
C -1.69405700 3.58364200 -0.75960900
H 0.22105200 2.86130400 -0.06306000
C -3.07693600 3.38210000 -0.76834100
H -4.70239900 2.13406600 -0.07105400
H -1.26091300 4.43475700 -1.29027500
H -3.72959900 4.07393500 -1.30585100
C 3.55777800 -0.05195300 -0.63602800
O 4.31251600 -0.73744800 -1.28565700
O 3.84893400 1.16273200 -0.17241700
C 5.15417200 1.67254500 -0.47679500
H 5.29000700 1.75233500 -1.56476000
H 5.20922200 2.66296100 -0.01183900
H 5.93023500 1.01145200 -0.06513400

P1_exo_proS_Bn

O -1.20700000 1.39678600 -1.30838400
O 0.72803900 3.32695400 -0.52998400
O -1.47508400 2.21038000 1.34446500
O 0.80840200 0.69676200 0.48898200
Ru -0.30508500 2.18006800 0.13488400
C 0.37831900 -2.12078200 1.08510200
C -1.80643000 -1.40195100 0.61310800
C -1.07376300 -2.51340100 1.34174300
H 0.71668400 -1.38525900 1.83063500
H 1.05989100 -2.98136500 1.09661400
H -1.82664100 -0.47211500 1.21226700
H -1.31749200 -2.55239100 2.41079300
H -1.32064600 -3.48244400 0.88571100
N 0.36158500 -1.53157200 -0.27383900
O -1.02529800 -1.20578700 -0.57842800
C 1.13767300 -0.32223300 -0.44782100
H -1.14354800 0.41199700 -1.24451400
C 2.61517300 -0.66213400 -0.31941400
C 3.16701600 -1.61298400 -1.19076400
C 3.42924900 -0.04456900 0.63550700
C 4.52099800 -1.94051000 -1.10655800
H 2.52781000 -2.09783900 -1.93146300
C 4.78623200 -0.37607200 0.71908200
H 2.99693400 0.69467600 1.30944400
C 5.33489800 -1.32222600 -0.14981200
H 4.94378100 -2.67985500 -1.79083600
H 5.41583600 0.11008000 1.46807000
H 6.39508200 -1.57801100 -0.08478700
H 0.93948100 0.03325900 -1.47148200
C -3.23105000 -1.71870500 0.18749600
O -3.86834300 -2.66677600 0.57876400
O -3.68745200 -0.79232000 -0.65372100
C -5.02633800 -0.96009700 -1.14081000
H -5.73989900 -0.96302800 -0.30462100
H -5.21632200 -0.10865000 -1.80317400
H -5.11626900 -1.90505000 -1.69538700

TS2_endo_Bn

C 0.43344300 2.19009900 -0.37713200
H 0.70713800 2.40697500 0.66142600
H 1.08674800 2.76551200 -1.04953400
C 0.55646100 0.70600700 -0.73155700
H 3.22355200 -1.54865000 -1.61048400
O 3.48825800 -1.73041000 -0.68881100

O 1.33016400 -0.57527500 1.20930500
O 4.06935900 0.79831300 0.80901000
O 1.77035800 0.18054200 -1.10884300
Ru 2.86553300 -0.30713400 0.38197100
N -0.44166000 0.46956300 -1.65684400
H 0.44865600 0.05581700 0.36513300
C -1.03983100 2.45340800 -0.68638300
O -1.37173100 1.50996300 -1.73134200
H -1.23988900 3.45273500 -1.08953300
C -0.96635400 -0.83800700 -2.00948000
H -0.08640400 -1.49302600 -2.06233700
H -1.39353000 -0.76526300 -3.02017700
C -1.98355400 -1.36122700 -1.01892600
C -3.34071500 -1.04289900 -1.16188700
C -1.57160400 -2.13273000 0.07679000
C -4.27642000 -1.50026700 -0.22986900
H -3.66530500 -0.43593300 -2.01035200
C -2.50665800 -2.59412900 1.00670800
H -0.51210700 -2.36555200 0.20802300
C -3.86111400 -2.28087400 0.85368900
H -5.33279100 -1.24970000 -0.35190300
H -2.17675300 -3.19921500 1.85442900
H -4.59231400 -2.64102500 1.58110500
C -1.99886600 2.22090300 0.48105700
O -2.95384600 2.92103100 0.71639700
O -1.66914100 1.13092700 1.17371200
C -2.53523900 0.75084300 2.25211000
H -3.53717000 0.52192400 1.86556600
H -2.08863200 -0.14609400 2.69187800
H -2.59767100 1.56097600 2.99179000

TSsec_exo_proR_Bn

O -1.68902700 0.31406300 -1.65381500
O -3.97769900 -0.38609900 0.06162700
O -1.47027000 -2.34861500 -0.11211600
O -1.57391900 -0.07555100 1.31062700
Ru -2.37736000 -0.82760800 -0.32832700
C 0.58016500 -1.91917400 1.23763200
C 1.98700300 -1.61108000 -0.64284500
C 1.49097900 -2.71040500 0.31705100
H -0.65946800 -2.29987500 0.76877500
H 0.53414100 -2.10184600 2.31744000
H 1.94459200 -1.91066700 -1.69987000
H 0.93088000 -3.49257300 -0.21382800
H 2.32750600 -3.18384400 0.84831600
N 0.69254300 -0.59661000 0.86353700
O 1.06226000 -0.53419900 -0.49691500
C -0.27655300 0.40320400 1.34733800
H -1.17094600 1.04399700 -1.26735300
H -0.03718600 0.49268400 2.42602300
C -0.06127600 1.76842700 0.71165600
C -1.17346600 2.59537400 0.50120100
C 1.22296400 2.25270800 0.42131000
C -1.00644700 3.88346800 -0.01904900
H -2.16845400 2.22636100 0.75358100
C 1.38600200 3.54030500 -0.09174700
H 2.09279200 1.61561900 0.57578800
C 0.27274400 4.35829400 -0.31728300
H -1.88078900 4.51685600 -0.18491100
H 2.38966900 3.90573400 -0.32169500
H 0.40437000 5.36422600 -0.72249800
C 3.43217700 -1.19785200 -0.33720100
O 4.28501900 -1.99690100 -0.02998000
O 3.63679700 0.10475500 -0.49566900
C 4.97317800 0.58111300 -0.27477900
H 5.66699000 0.11262300 -0.98694100
H 4.93597500 1.66461900 -0.43130300
H 5.29589400 0.35118900 0.75031600

TSsec_exo_proS_Bn

O -3.41547600 1.28449400 -0.80628900
O -3.80801100 -0.32975100 1.69028700
O -2.42430000 -1.59293200 -0.78390900
O -1.23144400 0.65677200 0.47698500
Ru -2.98029900 -0.23505500 0.22776700
C -0.07081800 -1.88739400 0.05162600
C 2.03018600 -2.14057100 -1.02320200
C 0.87362400 -2.97495400 -0.44725100
H -1.24431200 -1.94111300 -0.52005800
H -0.43237800 -1.90195400 1.09002000
H 2.43224300 -2.54522700 -1.96261600
H 0.39180300 -3.58374700 -1.22487300
H 1.21571400 -3.64258700 0.35327500
N 0.50177800 -0.68959200 -0.32329200
O 1.43994100 -0.88003600 -1.33892100
C -0.21278400 0.59103000 -0.45309900
H -2.70003500 1.93086800 -0.65122900

C 0.75359800 1.73765100 -0.24265000
C 0.71585600 2.85512300 -1.08267800
C 1.64222700 1.71007000 0.83996100
C 1.56578300 3.93995600 -0.84617000
H 0.02194500 2.87577500 -1.92730300
C 2.49239400 2.79164200 1.07561600
H 1.67242400 0.83122000 1.48656800
C 2.45488500 3.90887000 0.23266400
H 1.53574700 4.80924300 -1.50704200
H 3.18730900 2.76422700 1.91824900
H 3.12096200 4.75509800 0.41690600
H -0.60292400 0.62006900 -1.49168300
C 3.18888600 -2.00018900 -0.02780900
O 3.60266600 -2.93168100 0.62181300
O 3.67966900 -0.76875000 0.00556600
C 4.76362500 -0.52354300 0.91211400
H 4.47017400 -0.78795000 1.93778500
H 5.64506200 -1.11373700 0.62365300
H 4.97784900 0.54829500 0.84066800

TS2_exo_proR_Bn

O 3.29579800 -1.11930600 1.44352800
O 2.28822800 -0.54859900 -1.54076200
O 1.45551100 -3.00918800 0.05833300
O 1.08618600 -0.09271300 0.54436700
Ru 2.21162300 -1.50116200 -0.05534400
C -0.99562000 -1.17201000 -1.33866800
C -2.14950600 -0.52702300 0.66051000
C -1.53688600 -1.76881500 -0.02779500
H -0.08943300 -1.66033800 -1.71172800
H -1.76014600 -1.21532400 -2.12723000
H -1.72060900 -0.35846700 1.65945000
H -0.73461800 -2.19730400 0.58214000
H -2.28087000 -2.55263400 -0.22160400
N -0.75087000 0.23505000 -1.04071000
O -1.86294900 0.59075100 -0.20394000
C 0.46715100 0.65703000 -0.43572000
H 4.17328500 -1.52380200 1.32582300
H 1.37459900 0.35236900 -1.36180700
C 0.52834700 2.11725200 -0.17076200
C -0.25611600 3.02139900 -0.91451100
C 1.43474800 2.61831200 0.78309800
C -0.14682400 4.39231100 -0.69305600
H -0.94740300 2.63586700 -1.66331000
C 1.53358300 3.99111000 1.00586400
H 2.05009000 1.91809200 1.34779900
C 0.74406700 4.88236400 0.27001800
H -0.75972100 5.08509700 -1.27405600
H 2.23236100 4.36963900 1.75517700
H 0.82619000 5.95800100 0.44232600
C -3.65834300 -0.63392200 0.81401300
O -4.22217700 -0.74940400 1.87706100
O -4.27391900 -0.63760000 -0.36942400
C -5.69696300 -0.80244700 -0.35558100
H -6.17352500 0.00856100 0.21350300
H -6.01702800 -0.77230800 -1.40304500
H -5.96772100 -1.76658200 0.09890000

TS2_exo_proS_Bn

O -1.60223400 -3.07408400 1.07728000
O -0.47992200 -1.26870200 -1.23865800
O -3.33442200 -0.95734000 -0.17320300
O -0.68013500 -0.62504600 1.14372900
Ru -1.76897600 -1.59209500 -0.08093800
C 1.97059600 1.91733100 0.81664700
C 3.31087000 -0.00536200 1.12681400
C 3.36750600 1.51839500 1.29221500
H 1.58383100 2.81627900 1.31430100
H 1.95158400 2.08572700 -0.27165900
H 3.99933200 -0.53373700 1.80012900
H 3.51415100 1.76558500 2.35223300
H 4.16621900 1.98363100 0.70186800
N 1.15543200 0.74986200 1.17969200
O 1.98820800 -0.34338900 1.52495300
C 0.00255600 0.38272600 0.49574200
H -1.23389700 -2.76118000 1.92480400
C -0.83715800 1.55436900 0.04692300
C -0.72021100 2.08007900 -1.24566500
C -1.76588800 2.10212200 0.94341500
C -1.51652800 3.15894500 -1.63585300
H -0.01700700 1.63321100 -1.95154400
C -2.56238300 3.17930300 0.55003500
H -1.85972100 1.67622800 1.94424000
C -2.43708000 3.71004400 -0.73828100
H -1.42475200 3.56414800 -2.64587100
H -3.28532600 3.60421800 1.25001100
H -3.06337100 4.55057800 -1.04585300

H 0.21666500 -0.28552800 -0.58278800
C 3.64181000 -0.42733900 -0.30557300
O 4.76739100 -0.41297400 -0.74442100
O 2.55845900 -0.76876100 -0.99720300
C 2.73783300 -1.15822100 -2.36639600
H 3.17122000 -0.33018900 -2.94490300
H 3.40172600 -2.03178200 -2.42909900
H 1.73596800 -1.40601200 -2.73241800

P2_exo_Bn

O 3.36139600 -0.11351500 1.56547500
O 2.75639000 0.60311800 -1.46297300
O 4.16021300 -2.02936600 -0.72880600
O 1.11986600 -0.88154700 0.25000800
Ru 3.16371100 -0.78960100 -0.22019300
C -1.25032900 -2.24843400 0.15707600
C -3.22335200 -1.07272400 0.75151700
C -2.77058800 -2.39395200 0.11574800
H -0.81118500 -2.64098600 1.08684200
H -0.73995000 -2.71227400 -0.69700900
H -3.17518400 -1.12521200 1.85193000
H -3.13014300 -3.26845100 0.67014800
H -3.12939500 -2.44987900 -0.92179500
N -1.07488400 -0.79371400 0.11364500
O -2.28435000 -0.11038600 0.26294800
C 0.09301400 -0.14737500 0.18738000
H 4.16972200 0.42030400 1.62914900
H 1.83616400 0.51919800 -1.76495400
C 0.12247100 1.33614300 0.18495300
C -0.75849100 2.08796300 -0.61235500
C 1.08298800 1.98989000 0.97567000
C -0.67430700 3.48031800 -0.61686800
H -1.50335300 1.58561100 -1.22866300
C 1.14649200 3.38299200 0.97864900
H 1.78333100 1.39742200 1.56630900
C 0.27189500 4.13012400 0.18280800
H -1.35275000 4.06080600 -1.24563400
H 1.88796400 3.88846500 1.60133200
H 0.32884100 5.22114800 0.18324200
C -4.63798300 -0.67928500 0.36563400
O -5.59904300 -1.07882900 0.97844100
O -4.68585600 0.08186200 -0.72140900
C -5.99189900 0.45991500 -1.18235000
H -6.53003400 1.00944100 -0.39722700
H -5.83004300 1.10026300 -2.05611100
H -6.57148500 -0.43155000 -1.46121800

P2_exo_sec_Bn

O -0.24222100 -1.45983500 -0.90027900
O -3.22539400 -1.47108600 -1.46237900
O -2.30193700 -2.84406400 1.12358900
O -1.45726200 -0.38729600 1.30476900
Ru -2.04156000 -1.58399100 -0.28303700
C 2.06861700 -0.56110100 1.82204600
C 2.66868200 -0.81212600 -0.41299600
C 2.65430100 -1.59784000 0.90725500
H -2.45034500 -2.35500500 1.95255300
H 1.96346400 -0.65396600 2.90520200
H 2.21450900 -1.35566800 -1.24810800
H 1.99003400 -2.47518200 0.85804300
H 3.64772400 -1.93701700 1.23339300
N 1.62521500 0.47843100 1.23587600
O 1.83549800 0.35492400 -0.14854200
C -1.25480100 0.81710000 1.52502100
H 0.15031500 -0.59582100 -0.67328700
H -0.79092600 1.05630500 2.49684300
C -1.55895300 1.95183900 0.67262000
C -2.40248200 1.88244200 -0.45667900
C -0.97268400 3.18392900 1.03344000
C -2.63476600 3.02706500 -1.21375100
H -2.88893100 0.94438600 -0.72593400
C -1.19727700 4.32001000 0.26205200
H -0.32660900 3.23230600 1.91276800
C -2.02867100 4.24069500 -0.86165300
H -3.29458800 2.97844100 -2.08206300
H -0.73160500 5.26868000 0.53541000
H -2.21273500 5.13311200 -1.46413300
C 4.05540400 -0.34510000 -0.83379900
O 4.58088500 -0.64154200 -1.88051600
O 4.61625400 0.41850800 0.10449600
C 5.93001700 0.92389500 -0.16834900
H 6.21605800 1.51516300 0.70840200
H 6.63592700 0.09449900 -0.31818700
H 5.91895400 1.55463900 -1.06875200

P2_endo_Bn

C 0.44257500 2.31685800 -0.48709200

H 0.95817000 2.27333400 0.47838800
H 0.96768700 3.03703500 -1.13437500
C 0.42128900 0.97160900 -1.17183800
H 3.20224700 -0.95124300 -1.97686600
O 3.49697900 -1.34134700 -1.13475000
O 1.24385800 -1.13159000 1.19005000
O 3.76216500 0.56187400 1.24151100
O 1.31126900 0.09190600 -1.21958900
Ru 2.72550200 -0.33477700 0.28500800
N -0.75977000 0.82444700 -1.75696800
H 0.42430000 -0.66680100 0.94837300
C -1.05328800 2.61084900 -0.38914100
O -1.63087700 1.88687700 -1.50987300
H -1.32798400 3.66193700 -0.52335200
C -1.36272100 -0.39063200 -2.28758600
H -0.52955400 -0.98381700 -2.68514500
H -2.01151200 -0.09504500 -3.12301700
C -2.11712100 -1.13857200 -1.21211000
C -3.44917300 -0.81383900 -0.92237600
C -1.46430400 -2.11663200 -0.44787700
C -4.12375800 -1.46558000 0.11384000
H -3.95920200 -0.04701200 -1.50990200
C -2.13863300 -2.76666500 0.58881300
H -0.42133600 -2.36359400 -0.65568200
C -3.46987800 -2.44398600 0.86983400
H -5.16267900 -1.20825000 0.33204600
H -1.62031000 -3.52482700 1.18006400
H -3.99677400 -2.95157300 1.68100000
C -1.71108300 2.09617100 0.89375800
O -2.61326800 2.65805600 1.46036200
O -1.18129400 0.92963700 1.28316800
C -1.74426000 0.31712100 2.45813500
H -2.79097600 0.04568100 2.27155800
H -1.14432600 -0.57972000 2.64217200
H -1.67997300 1.00943100 3.30795900

3-isoxazolidinone

C -2.20746200 -0.79457300 0.45434800
C -1.92982900 -1.44976100 -0.90559300
H -2.85233800 -1.42634200 1.08431100
H -2.64522600 -2.24161600 -1.16003200
H -1.90714300 -0.72240500 -1.73160500
N -0.06202600 -1.55926300 0.45455800
O -0.92395800 -0.64152800 1.08524500
C 1.30377900 -1.52382500 0.90072700
H 1.75848700 -2.45358200 0.52808300
C 2.11106800 -0.32703400 0.42371800
C 3.50531400 -0.35322000 0.57871500
C 1.51183700 0.79772000 -0.15851100
C 4.28874400 0.72313900 0.15715500
H 3.98251300 -1.22844400 1.02884900
C 2.29821400 1.87442300 -0.58378700
H 0.42836200 0.84700400 -0.26766200
C 3.68624600 1.84252400 -0.42811500
H 5.37382600 0.68608700 0.28083300
H 1.81752900 2.74507800 -1.03687300
H 4.29761500 2.68456000 -0.76116600
H 1.30365300 -1.58176000 2.00117400
C -2.86508900 0.57306800 0.33771700
O -4.01554400 0.78983200 0.63259000
O -2.02978100 1.47610900 -0.17533900
C -2.54504200 2.79934800 -0.37669700
H -2.88775600 3.22515600 0.57693200
H -1.71490500 3.38787300 -0.78215200
H -3.38426600 2.77893900 -1.08640500
C -0.52292100 -2.00764000 -0.74274700
O 0.10111700 -2.71325200 -1.51721800

TS1b_ exo _proR_Bn

O -1.69604900 -0.38127200 -1.10923900
O -4.31212300 0.64293700 -0.69676900
O -3.68678500 -2.16214800 -0.16734600
O -2.53935700 -0.11057600 1.29315300
Ru -3.20806400 -0.54626800 -0.23341300
C 2.71058000 -1.72614500 -0.88697800
C 2.13141300 -2.77136700 0.06491400
H 2.83873500 -2.08819800 -1.91604700
H 1.51041900 -3.51095800 -0.46348500
H 2.89420800 -3.30086300 0.64763100
N 1.13568800 -0.68330700 0.32586600
O 1.71054700 -0.69931500 -0.94720000
C 0.15278000 0.26327800 0.60182100
H -0.78067400 -0.01662500 -0.24231200
H -0.25614200 0.02118400 1.58422000
C 0.37682300 1.71017200 0.35987300
C -0.64596300 2.59484300 0.75785300
C 1.53967900 2.22392200 -0.24237500
C -0.49489600 3.96885800 0.58529400

H -1.55700100 2.18800200 1.20193000
C 1.68479100 3.60264200 -0.40495500
H 2.33199100 1.54870300 -0.56024200
C 0.67247500 4.47820200 0.00319500
H -1.29069400 4.64632300 0.90252000
H 2.59857000 3.99626000 -0.85614100
H 0.79024600 5.55524500 -0.13582900
C 4.06950800 -1.20891700 -0.39627800
O 4.94631400 -1.95772200 -0.03762300
O 4.16469000 0.11378200 -0.43673800
C 5.40081600 0.69087600 0.01417200
H 6.22921700 0.36805500 -0.63158300
H 5.26471600 1.77586900 -0.04709600
H 5.60590500 0.38582600 1.04971600
C 1.22973000 -1.93189900 0.94648400
O 0.67014700 -2.21027200 1.98147500

TS1b_ exo _proS_Bn

O 0.34125600 1.44324700 -1.44691100
O 0.78584900 3.92588000 -0.10519100
O -1.66993900 2.35544300 0.28583500
O 0.82768200 1.44245500 1.10212300
Ru -0.01193800 2.43785900 -0.03388200
C -2.21498100 -0.94283000 0.54552700
C -1.54905900 -1.53700000 1.77116600
H -2.32580600 0.15449400 0.61972100
H -1.72086900 -0.96484100 2.69063000
H -1.86920300 -2.57760700 1.93846800
N -0.02735300 -1.31132000 0.02053200
O -1.29276300 -1.22776700 -0.54168800
C 0.99548500 -0.95500100 -0.85721100
H 0.84143600 0.34135400 -1.08053900
C 2.42186100 -1.25047200 -0.57208400
C 3.13989800 -0.64423700 0.47402700
C 3.09171800 -2.12894800 -1.44536600
C 4.48424800 -0.95186100 0.66998800
H 2.63132400 0.08135800 1.10743200
C 4.43781400 -2.43721400 -1.24329900
H 2.54852300 -2.58380100 -2.27675800
C 5.13584700 -1.85221400 -0.18255900
H 5.03431000 -0.47601000 1.48483400
H 4.94230800 -3.13127700 -1.91894100
H 6.19234400 -2.08396100 -0.02914900
H 0.68589000 -1.24245300 -1.87223600
C -3.55253200 -1.56142200 0.16034800
O -4.11778600 -2.41470000 0.79984200
O -4.00303000 -1.02198100 -0.96612800
C -5.25077100 -1.52063500 -1.47315400
H -6.05450700 -1.34528200 -0.74418200
H -5.44432200 -0.96627700 -2.39769200
H -5.17631900 -2.59777700 -1.67890300
C -0.07801200 -1.50343600 1.40592000
O 0.88654800 -1.62543300 2.12019200

P1b_exo_proR_Bn

O -1.29120700 -0.08732600 -1.71812500
O -3.74489500 0.05317600 -0.33206000
O -2.16885300 -2.56973600 -0.74328500
O -1.43317800 -0.55191700 1.13637000
Ru -2.38860600 -0.93132900 -0.45786000
C 2.23186700 -1.40010100 -0.68398300
C 2.09891100 -2.42955400 0.43434300
H 2.11365600 -1.82374100 -1.69105900
H 1.46617800 -3.27941600 0.13628900
H 3.06236100 -2.81098900 0.79188300
N 0.92950900 -0.46915000 0.91711300
O 1.12442600 -0.51508600 -0.48881100
C -0.25974700 0.22171100 1.33925500
H -0.39538100 0.07948300 -1.35896700
H -0.16382000 0.23377700 2.43941200
C -0.34895800 1.64450600 0.82003300
C -1.58180600 2.31088400 0.87257600
C 0.78352000 2.32464000 0.35230700
C -1.68472700 3.63509400 0.44118700
H -2.46467800 1.79201600 1.24705800
C 0.67664900 3.65042700 -0.07620400
H 1.74455800 1.81517400 0.30336300
C -0.55609800 4.30801400 -0.03730700
H -2.65095600 4.14299300 0.48121400
H 1.56405400 4.16878500 -0.44694200
H -0.63726800 5.34313000 -0.37708300
C 3.58417000 -0.67743900 -0.63670200
O 4.62278600 -1.27147500 -0.47151100
O 3.47804300 0.63099400 -0.83257700
C 4.70083300 1.38502400 -0.83408600
H 5.35868500 1.04049500 -1.64413900
H 4.40974800 2.42896100 -0.99243000

H 5.21910400 1.27110800 0.12828300
C 1.35798400 -1.65207300 1.50776700
O 1.15666100 -1.94688700 2.66425300

P1b_exo_proS_Bn

O 1.24584900 1.65312900 1.15139400
O -0.88973800 3.46942300 0.66475700
O 1.10718600 2.58397500 -1.49848300
O -0.95175700 0.87844300 -0.43011200
Ru 0.12575400 2.40852000 -0.14949800
C 2.06614500 -1.28754300 -0.63777300
C 1.32396600 -2.34991700 -1.42440900
H 2.19598200 -0.36302400 -1.22871700
H 1.51507200 -2.32111100 -2.50323100
H 1.54860500 -3.36118800 -1.05084800
N -0.13286000 -1.28307500 0.05535100
O 1.18907200 -1.00548900 0.48604600
C -1.06006100 -0.23483700 0.42949600
H 1.23914000 0.67000900 1.10230400
C -2.49056100 -0.75336000 0.42849800
C -2.81584200 -1.84163600 1.24918900
C -3.48425000 -0.13686100 -0.33606400
C -4.12801000 -2.31420700 1.29700800
H -2.03759400 -2.32242100 1.84667100
C -4.79855000 -0.61283500 -0.28631800
H -3.22457500 0.71075400 -0.96958300
C -5.12367000 -1.70094200 0.52742100
H -4.37549200 -3.16313000 1.93852000
H -5.57114400 -0.12875800 -0.88834700
H -6.15106200 -2.07043800 0.56577700
H -0.79089000 0.04379800 1.46099700
C 3.41345100 -1.69917300 -0.06412300
O 3.99985900 -2.70960700 -0.36532300
O 3.84984000 -0.78401600 0.79482700
C 5.10859600 -1.04081900 1.43709900
H 5.90756300 -1.12740400 0.68741900
H 5.29133800 -0.18528700 2.09588600
H 5.05549300 -1.97093500 2.02041200
C -0.12865700 -2.00036400 -1.13465700
O -1.11292700 -2.28201500 -1.77798000

TS2b_exo_proR_Bn

O -1.56960500 -3.00519300 -1.64015000
O -1.63620200 -1.71474400 1.30872100
O 0.92278900 -2.62303200 -0.03036000
O -1.11487500 -0.61811400 -0.81558500
Ru -0.73795500 -2.32702600 -0.08166200
C 2.44672500 0.23258700 0.48082100
C 2.17419400 0.61608000 1.92915100
H 2.52145500 -0.86423300 0.36437600
H 2.62128600 -0.06809000 2.66016300
H 2.51185900 1.64006600 2.15314700
N 0.22828800 0.54466700 0.68961600
O 1.27607900 0.68187700 -0.23948100
C -1.07016100 0.34374200 0.14247400
H -1.77818300 -3.94783800 -1.51660900
H -1.61910000 -0.42271000 1.10012200
C -1.88713300 1.54601800 -0.13738600
C -1.43097100 2.83447700 0.19055600
C -3.16204300 1.38144200 -0.71120100
C -2.23753600 3.94371600 -0.06154700
H -0.43956100 2.96241800 0.62718700
C -3.96032100 2.49404300 -0.96650600
H -3.51667800 0.37717000 -0.94940500
C -3.50111000 3.77673500 -0.64016100
H -1.87767400 4.94391800 0.18871800
H -4.94792300 2.36391000 -1.41404400
H -4.13130000 4.64744700 -0.83467200
C 3.66308300 0.88660500 -0.15288000
O 4.53059100 1.44574300 0.47422600
O 3.65174700 0.73573100 -1.47298200
C 4.74637600 1.31208500 -2.20087100
H 5.69499800 0.84830400 -1.89513800
H 4.54567300 1.11047100 -3.25866400
H 4.79853400 2.39502200 -2.01967800
C 0.65583800 0.54762700 1.99479900
O -0.07715200 0.48519300 2.96021100

TS2b_exo_proS_Bn

O 2.76240300 -2.65267900 -0.77808600
O 1.07916600 -1.14951600 1.39724200
O 3.65911200 0.03045500 0.21046800
O 1.09959000 -0.64332100 -1.01595500
Ru 2.42270000 -1.11805700 0.25193000
C -3.04047500 -1.33001600 -0.89762900
C -3.31853500 -0.17666900 -1.86512300
H -3.46347400 -2.28946800 -1.22315100

H -3.39533800 -0.52777900 -2.90549700
H -4.21419800 0.40803000 -1.62222400
N -1.14611400 -0.14942800 -1.04826500
O -1.61821800 -1.46821300 -0.93182200
C 0.09300000 0.10203600 -0.45569100
H 2.31332200 -2.56891900 -1.64050100
C 0.36813800 1.52178700 -0.09942500
C -0.48420600 2.19212100 0.79556500
C 1.49626700 2.17895200 -0.61484700
C -0.21601500 3.50775500 1.16069800
H -1.35027300 1.66982300 1.20721100
C 1.76249300 3.49844600 -0.24013400
H 2.15099400 1.65890700 -1.31388600
C 0.91018700 4.16433200 0.64404700
H -0.88029000 4.02466400 1.85656000
H 2.63855100 4.00862100 -0.64643600
H 1.12122100 5.19596300 0.93452400
H 0.15602900 -0.51889100 0.72412300
C -3.57275600 -1.02441600 0.50653300
O -4.75109800 -0.87769600 0.72542200
O -2.60831200 -0.93141100 1.41753600
C -3.01454800 -0.63703000 2.76358400
H -3.54381500 0.32550600 2.79911900
H -3.67467200 -1.42927600 3.14317800
H -2.09147500 -0.59032700 3.35094900
C -2.05927800 0.66594200 -1.71339200
O -1.85867100 1.80416000 -2.06432400

Isoxazoline_cy

C -1.59415600 -2.19803800 -0.38802800
H -2.06040400 -2.01591000 -1.36376600
H -1.98115300 -3.14302800 0.01329200
C -0.05384500 -2.20348900 -0.44441800
H 0.36568800 -3.05006400 0.13526000
N 0.30356800 -0.91502600 0.15573400
H 0.33340300 -2.25268800 -1.47049100
C -1.86852400 -1.02502000 0.57661500
O -0.65110600 -0.81656100 1.25159100
H -2.62934400 -1.25701900 1.33846600
C 1.64126700 -0.80625700 0.72687800
C 2.68435900 -0.94977000 -0.39397200
C 1.80998500 0.55251000 1.42408300
H 1.78917800 -1.62047000 1.46860200
C 2.61386400 0.20477200 -1.40007300
H 2.58071300 -1.92133700 -0.90157500
H 3.67678000 -0.95965700 0.08749000
C 1.73122500 1.71883800 0.43380300
H 1.04986900 0.65926300 2.21063400
H 2.79470200 0.55023400 1.92280400
C 2.75089800 1.56205300 -0.70009200
H 1.64835600 0.16440500 -1.93159400
H 3.40248100 0.08433500 -2.16112100
H 3.77129800 1.64861200 -0.28260700
H 2.64210400 2.38100600 -1.43078100
H 0.71303100 1.75582900 0.01455000
H 1.89395600 2.67175000 0.96452700
C -2.33203300 0.23211200 -0.17152200
O -2.99275400 0.21344500 -1.18569900
O -1.96798700 1.35114400 0.45538300
C -2.36034700 2.58379600 -0.15733000
H -3.45511700 2.64415800 -0.24186600
H -1.98305100 3.38105100 0.49296100
H -1.91885900 2.67143900 -1.16078100

TS1_cy_endo_$^4C_1$

C 0.33734500 -1.64189900 1.18178700
H 0.90695400 -2.02797000 2.03690100
H -0.65866000 -2.10015300 1.19617000
C 0.24365800 -0.12030400 1.18111800
H -0.97125600 0.21967900 1.07752900
O -2.26032400 0.50946500 1.25980700
O -4.06185600 -1.45410000 0.33721800
O -3.84143800 1.15707800 -1.01925200
O -1.74635300 -0.66269400 -0.97815100
Ru -3.15085700 -0.10607400 -0.12827100
N 0.99470000 0.31498500 0.09997900
H 0.47714900 0.40676300 2.11580700
C 1.08076200 -1.90846800 -0.14134300
O 1.02348600 -0.68032100 -0.86292100
H 0.58346400 -2.66060500 -0.76912100
C 0.89599900 1.63778200 -0.51218000
C 0.80021900 2.72086500 0.56793800
C 2.10071900 1.88149700 -1.43149700
H -0.03872300 1.61673000 -1.10655200
C 2.10175900 2.86948500 1.36493000
H -0.06282700 2.53745500 1.22580600
H 0.58333200 3.66310300 0.03936000

C 3.40183300 2.00466800 -0.63268400
H 2.16921800 1.07494000 -2.17455300
H 1.90374400 2.81488600 -1.98412700
C 3.30187300 3.09660700 0.43812400
H 2.27615700 1.96183600 1.96835700
H 2.00178900 3.70224700 2.07907500
H 3.19408900 4.07889500 -0.05674700
H 4.23209300 3.14228900 1.02746200
H 3.61758800 1.03345400 -0.15521400
H 4.23968100 2.21281000 -1.31728800
C 2.52187900 -2.36908200 0.09770200
O 2.79575800 -3.23084200 0.89978000
O 3.40135000 -1.74467400 -0.67644300
C 4.77670200 -2.12594200 -0.52373400
H 4.90738900 -3.19037100 -0.76457600
H 5.34229200 -1.50133100 -1.22341100
H 5.10986100 -1.94485800 0.50794300

TS1_cy_endo_$^{1}C_{4}$

C 0.82461600 -1.89654300 1.19373800
H 1.47125400 -2.28053300 1.99365700
H -0.05965600 -2.54146800 1.12770700
C 0.42798600 -0.44316800 1.41445800
H -0.82393000 -0.33354200 1.26065000
O -2.15481400 -0.27321900 1.33851000
O -3.60159600 -2.18139200 -0.16649100
O -3.55642800 0.67088700 -0.93292400
O -1.27643100 -0.91425500 -1.01035400
Ru -2.81365200 -0.68405600 -0.24165300
N 1.12651100 0.29686700 0.47180000
H 0.50495800 -0.03484700 2.43110300
C 1.59344600 -1.81881100 -0.13899300
O 1.38276900 -0.49619900 -0.63355700
H 1.19022800 -2.49801200 -0.90352300
C 0.79087600 1.65296900 0.05712700
C 0.25192400 2.47566300 1.22886700
C 1.99902700 2.32532800 -0.60578200
H -0.01670500 1.53382300 -0.69351200
C -0.08706400 3.89795500 0.76687700
H 1.01313100 2.51073500 2.02727500
H -0.64684900 1.99737800 1.64544000
C 1.64904000 3.74393600 -1.06166000
H 2.82109300 2.35533200 0.13004300
H 2.34291300 1.71156000 -1.45060700
C 1.10747000 4.58473300 0.09845700
H -0.44135800 4.48580500 1.62817400
H -0.92789900 3.84960900 0.05206500
H 2.53773400 4.22011400 -1.50535900
H 0.88823600 3.69098200 -1.86108400
H 0.81920400 5.58736900 -0.25643300
H 1.90806900 4.73126100 0.84578600
C 3.08230300 -2.13476500 0.02770000
O 3.47981600 -3.02387300 0.74374800
O 3.85565800 -1.36086800 -0.72365600
C 5.26774600 -1.61080400 -0.66350900
H 5.48989900 -2.63472200 -0.99616000
H 5.73282700 -0.88061700 -1.33458600
H 5.63597900 -1.47745500 0.36360100

TS1_cy_exo_$^{4}C_{1}$

C 0.24353600 1.55878400 0.16287500
H -0.90114500 1.15644600 0.30060700
O -2.26595700 0.93817600 0.69601600
O -2.89392900 -1.77504100 1.09802400
O -4.08758100 -0.24142000 -1.11929500
O -1.37293200 -0.81180900 -1.00415300
Ru -2.79831600 -0.54332700 -0.06244600
C 1.15299500 -0.29769600 1.62078000
C 1.38003700 -1.70366300 -0.28560900
C 0.84412800 -1.72222200 1.15306300
H 0.41020300 0.08734300 2.32908500
H 2.15243900 -0.22787200 2.07637600
H 0.75840200 -2.29183600 -0.97218300
H -0.23544800 -1.91582700 1.14839300
H 1.33447000 -2.48121100 1.77537000
N 1.12306300 0.48889000 0.39003400
O 1.29718100 -0.33882700 -0.70496200
C 0.37690800 2.64683700 1.23300000
H -0.48964900 3.31696300 1.12065400
H 0.31503600 2.22142500 2.24520400
C 0.32785200 2.14229500 -1.24490200
C 1.68275400 3.43659000 1.04969700
H 1.73043000 4.24205900 1.79991400
H 2.53475300 2.76325300 1.24622100
C 1.79763300 4.01065200 -0.36578900
H 2.76464500 4.52557800 -0.48741600
H 1.01372700 4.77613100 -0.50949800
C 1.63773800 2.92377700 -1.43362500

H 0.22070300 1.34407900 -1.98829100
H -0.53262400 2.82019200 -1.36261500
H 1.65814000 3.36693200 -2.44215200
H 2.48329400 2.21697600 -1.37686400
C 2.81400100 -2.21567400 -0.36737600
O 3.09129000 -3.35941700 -0.63915800
O 3.69959900 -1.27356400 -0.04578400
C 5.08046700 -1.66234200 -0.03135100
H 5.38902800 -2.01140500 -1.02692700
H 5.64452100 -0.76700900 0.25163300
H 5.24388500 -2.46685900 0.69978300

TS1_cy_exo_$^1C_4$

C -0.43271300 1.71909800 0.12616700
H -1.03093900 0.66131500 0.39066300
O -1.69450100 -0.39532700 1.09952100
O -0.51225800 -2.91340000 0.63283400
O -3.14640600 -2.20698400 -0.52087600
O -0.77981400 -0.91350500 -1.22680200
Ru -1.60556800 -1.77065100 0.02337300
C 1.46629600 0.98909900 1.62746800
C 2.11666900 -0.45962300 -0.15879900
C 1.79821300 -0.48042100 1.34819700
H 0.73496200 1.12454100 2.43023900
H 2.37411800 1.56404000 1.86388100
H 1.63979600 -1.28907300 -0.69874900
H 0.93868000 -1.13182800 1.54437600
H 2.64276700 -0.83415700 1.95301400
N 0.92525500 1.46037900 0.36101500
O 1.57070500 0.78043600 -0.66031200
C -1.05812200 2.68500000 1.13848400
H -0.86842900 2.35501100 2.16973400
H -0.55969400 3.66306600 1.01567400
C -0.75685300 2.10676800 -1.31560700
C -2.56674000 2.82892600 0.90663500
H -2.96718700 3.57498800 1.61073400
C -2.26583000 2.23707200 -1.52919600
H -0.26888200 3.08067500 -1.50722700
H -0.32316200 1.37232000 -2.00053600
H -2.45823100 2.55756500 -2.56528500
C -2.89166400 3.22055100 -0.53712400
H -3.98327300 3.26796400 -0.67980600
H -2.50192400 4.23498800 -0.73726800
H -3.05658300 1.87027500 1.14566600
H -2.73281100 1.24408800 -1.41495300
C 3.61290400 -0.50451000 -0.43045600
O 4.18432600 -1.47966200 -0.85666300
O 4.21107900 0.63502600 -0.08378400
C 5.63650400 0.68833500 -0.23352400
H 5.91791000 0.51909200 -1.28254400
H 5.93804300 1.69175600 0.08615100
H 6.11599000 -0.07505800 0.39590200

IP_cy_exo_$^4C_1$

C 0.57376000 1.37628300 -0.12346300
H -2.34727500 0.90528800 -1.24720700
O -3.09309000 0.89091500 -0.59876600
O -2.15818100 -0.74568100 1.68183500
O -3.90847700 -1.94399200 -0.53350700
O -1.37478000 -0.77575400 -1.01073600
Ru -2.79035100 -0.83525000 0.09739200
C 0.93219200 -0.81701300 1.03672500
C 2.19199900 -1.65176100 -0.78406200
C 1.20354600 -2.11624100 0.28926700
H -0.07875900 -0.76496900 1.45682900
H 1.68886500 -0.59762300 1.80263000
H 2.13741200 -2.24134600 -1.70706700
H 0.27924900 -2.45516800 -0.19455600
H 1.61394200 -2.90075800 0.93596500
N 1.06735100 0.19100100 -0.03619500
O 1.75201200 -0.32697400 -1.11851100
C -0.23546000 1.95370300 0.99384000
H -1.27910000 1.94061600 0.63036300
H -0.20302600 1.32752100 1.89128100
C 0.76170100 2.20354700 -1.35524500
C 0.18422000 3.40258300 1.29938000
H -0.51330400 3.81441300 2.04378500
H 1.18461300 3.39978300 1.76415700
C 0.20670400 4.25992200 0.03367600
H 0.52716700 5.28485700 0.27729900
H -0.81484000 4.33449600 -0.37960000
C 1.14209600 3.65535400 -1.01361700
H 1.48877300 1.73994500 -2.02932200
H -0.21676300 2.18745700 -1.86938700
H 1.13141800 4.24468600 -1.94253300
H 2.17866800 3.67254000 -0.63662300
C 3.63536900 -1.66178100 -0.28790400
O 4.34354900 -2.63559400 -0.36891400

O 3.97640400 -0.50862400 0.28213300
C 5.29774300 -0.42583600 0.83912800
H 6.05069500 -0.58149700 0.05396900
H 5.38514500 0.58141400 1.26004500
H 5.42838400 -1.18456200 1.62345500

TS_cy_IP_exo_$^4C_1$

C 0.35590700 1.01100800 -0.14088700
H -3.50788900 1.66720500 -0.09093000
O -4.04157600 0.94637800 0.32128900
O -2.09190400 -0.97597800 1.40908500
O -3.52130900 -1.47620300 -1.25042600
O -1.87587400 0.79717700 -0.75978400
Ru -2.89308300 -0.49999700 -0.01692000
C 0.92364700 -0.97923000 1.30147800
C 2.02079000 -2.01348500 -0.52070100
C 1.29355600 -2.36547900 0.78267600
H -0.03332000 -0.95257100 1.83052500
H 1.71822100 -0.53765800 1.91943300
H 1.98087100 -2.81071800 -1.27306800
H 0.39414400 -2.95397200 0.55671400
H 1.92530100 -2.92480900 1.48258400
N 0.80604100 -0.19108000 0.05482900
O 1.26979500 -0.91857700 -1.04245600
C 0.00633900 1.85204400 1.04822900
H -0.88366300 2.43350300 0.77247000
H -0.25546300 1.24248700 1.92061000
C 0.66781000 1.75710800 -1.39967800
C 1.19302000 2.79729400 1.33987200
H 0.91155900 3.46908900 2.16512800
H 2.05495300 2.20392400 1.69008300
C 1.58815200 3.59739000 0.09418000
H 2.47410300 4.21439000 0.31280000
H 0.77136100 4.29431900 -0.16386200
C 1.86920300 2.68361000 -1.10294000
H 0.87935400 1.07350500 -2.22798500
H -0.21786300 2.35372100 -1.65466600
H 2.08805900 3.27575200 -2.00474800
H 2.75502100 2.05980600 -0.90109200
C 3.48495000 -1.66460200 -0.25262300
O 4.32268900 -2.51089300 -0.05603100
O 3.69425800 -0.35116300 -0.19241000
C 5.01822000 0.08186100 0.15941300
H 5.74726800 -0.28345000 -0.57664100
H 4.98661200 1.17662600 0.15863100
H 5.28652000 -0.29477800 1.15643000

P1_cy_endo_$^4C_1$

C -1.40941600 -0.99043600 1.32250100
H -1.54982000 -2.07813500 1.33715200
H -1.10833400 -0.66046400 2.32498200
C -0.34313900 -0.57446000 0.28955600
H 2.67219300 -0.53861000 2.04532900
O 3.34822900 -0.81626600 1.39437700
O 2.83041100 0.09036100 -1.24839100
O 2.37813000 -2.82138100 -0.40342500
O 0.93506400 -0.65222100 0.83770800
Ru 2.42911700 -1.15772800 -0.20193300
N -0.72579200 0.79157400 -0.11755800
H -0.44478300 -1.20564800 -0.60849200
C -2.63201200 -0.21766800 0.80807700
O -2.12832900 0.61197000 -0.27269200
H -3.09753500 0.40674400 1.58516800
C -0.42471200 1.87534700 0.85803300
H -0.50585900 1.50101200 1.89607900
C -1.41264500 3.03932800 0.68270700
H -1.18537700 3.76757300 1.47987300
H -2.44382400 2.70002300 0.84944900
C 1.00443500 2.40146200 0.63005800
H 1.20099000 3.14263800 1.42417500
H 1.74394900 1.60496300 0.76842900
C 1.16588900 3.06430600 -0.74063100
H 2.19567600 3.44199600 -0.85108200
H 1.02160600 2.30264400 -1.52341600
C -1.28035100 3.72052300 -0.68336000
C 0.15417100 4.19882500 -0.93466500
H 0.24387700 4.62567800 -1.94766800
H 0.38955100 5.01627300 -0.22816300
H -1.98229500 4.56881600 -0.74367700
H -1.57683800 3.00756000 -1.47016800
C -3.71336900 -1.11942800 0.23545400
O -4.83984300 -1.18874100 0.66811800
O -3.25301500 -1.84662000 -0.78462600
C -4.16996200 -2.76758000 -1.38801900
H -3.62073300 -3.25538100 -2.20084300
H -4.50558300 -3.51266400 -0.65220500
H -5.04599900 -2.23415200 -1.78378000

P1_cy_endo_$^1C_4$

C -1.51944500 -0.53389600 1.33474900
H -1.78061500 -1.54008300 1.68492700
H -1.15990000 0.05437800 2.18866400
C -0.44252300 -0.58049400 0.23427100
H 2.71428100 -0.05498500 1.60503500
O 3.31669500 -0.58640000 1.04626500
O 2.38332600 -0.88390400 -1.63980300
O 2.23079300 -3.16155300 0.41566200
O 0.84062500 -0.63497800 0.77239400
Ru 2.22258900 -1.57100000 -0.11849300
N -0.67156900 0.64084700 -0.56824100
H -0.64679800 -1.43601100 -0.43029600
C -2.66147400 0.17669800 0.59481800
O -2.08955600 0.60388900 -0.67018300
H -3.05161800 1.04300400 1.14975200
C -0.20075100 1.91389500 0.04453400
H -0.23541400 1.85587600 1.15103000
C -1.09096400 3.08391500 -0.39489500
H -2.12664300 2.91993700 -0.06899600
H -1.10774600 3.11268200 -1.49845600
C 1.25198700 2.18237200 -0.37795500
H 1.90866800 1.37365000 -0.04218000
H 1.28858300 2.18893700 -1.48063500
C 1.76826800 3.51442400 0.17464700
H 1.80425900 3.45920200 1.27802200
H 2.80528800 3.67511800 -0.16243800
C -0.57807500 4.41838900 0.15572300
C 0.87519100 4.68317700 -0.24353500
H -1.23132900 5.23451500 -0.19364900
H -0.65475900 4.40753700 1.25825300
H 0.93420600 4.81078800 -1.33971200
H 1.23316400 5.62475900 0.20456100
C -3.83908100 -0.73187000 0.27844700
O -4.96395100 -0.55253400 0.68260700
O -3.46920600 -1.76245200 -0.48483800
C -4.49034200 -2.70085600 -0.84508800
H -4.00085100 -3.45996000 -1.46515100
H -4.92404000 -3.16202300 0.05389100
H -5.28822900 -2.20030200 -1.41233900

P1_cy_exo_$^4C_1$

C -0.10598800 1.51470700 0.19272200
O -3.80480800 0.01885500 -0.43068100
O -2.12315300 -1.52221200 1.07962700
O -1.69989100 -0.82451400 -1.93302100
O -1.51910500 1.20708600 0.18806700
Ru -2.00590400 -0.55032200 -0.29811500
H -3.80894200 0.97227900 -0.22754300
C 0.46356200 0.00069000 2.13335000
C 0.94752100 -1.83397800 0.63559500
C 0.86358600 -1.48697700 2.13240100
H -0.58056200 0.13327800 2.45847000
H 1.11835200 0.60866500 2.76991100
H 0.40602000 -2.75400500 0.37243500
H 0.10029800 -2.09396200 2.63098400
H 1.83122100 -1.66817500 2.61454500
N 0.66964100 0.41443900 0.73562100
O 0.28294400 -0.76670500 -0.03587300
C 0.35497500 1.81700800 -1.24125200
C 0.07613500 2.75306600 1.07864400
H -0.26339700 2.53413100 2.10123100
H -0.60528200 3.51841200 0.67626000
C 1.52036600 3.26729600 1.05426300
H 1.58713200 4.18260200 1.66349600
H 2.18290100 2.52281700 1.52650900
C 1.99503300 3.53876800 -0.37687200
C 1.80242800 2.31435200 -1.27672500
H 0.23302200 0.92232100 -1.86603200
H -0.32614900 2.58736900 -1.63653200
H 3.05280300 3.84781100 -0.37197200
H 1.42035700 4.38643600 -0.79183500
H 2.46435700 1.49780300 -0.94570700
H 2.08169900 2.55316200 -2.31540700
C 2.38625300 -1.96731000 0.13000200
O 3.27470100 -2.44881200 0.79265900
O 2.52066600 -1.54066300 -1.12097900
C 3.82711300 -1.65128900 -1.70227400
H 4.55449800 -1.06825800 -1.11986600
H 4.14630100 -2.70283600 -1.73125600
H 3.74090500 -1.24900800 -2.71748000

P1_cy_exo_$^1C_4$

C -0.79332800 1.13482100 0.43132800
O -2.63995100 -2.34109600 -0.41493700
O -0.26250600 -2.52217000 0.93755400
O -0.59255200 -1.47563200 -1.99466500

O -1.64775500 -0.03117200 0.34529600
Ru -0.89675200 -1.61457000 -0.34094600
H -3.23982300 -1.63305800 -0.11526000
C 0.77141500 0.18355800 2.17083900
C 2.23596900 -0.69287400 0.46013700
C 2.04457600 -0.66453300 1.98622600
H -0.07682600 -0.43975700 2.49492600
H 0.91216900 1.00085000 2.88860800
H 2.43143900 -1.70010700 0.06518100
H 1.89498200 -1.67654500 2.37731200
H 2.92536400 -0.22402200 2.46751900
N 0.55278900 0.77732000 0.83915200
O 0.98460800 -0.27929600 -0.08229600
C -0.74874500 1.82942900 -0.93852600
C -1.42229100 2.04872300 1.49323800
H -0.73271700 2.89273200 1.65592700
H -1.50650900 1.49656200 2.43998600
C -2.79763300 2.55979700 1.05433200
H -3.49412900 1.70682200 1.00486300
H -3.18655100 3.24586100 1.82321300
C -2.74067300 3.25141500 -0.30986800
H -3.74975000 3.57201800 -0.61524700
H -2.12792100 4.16763500 -0.22864300
C -2.12890900 2.32912900 -1.36758000
H -2.04468900 2.84994100 -2.33456900
H -2.79046100 1.46148700 -1.52964500
H -0.04402200 2.67132800 -0.85013200
H -0.33172400 1.13837400 -1.68366700
C 3.35829700 0.23262400 -0.01736100
O 4.38272600 0.39711300 0.60247900
O 3.09052000 0.78379500 -1.19576500
C 4.09222300 1.65427600 -1.73990600
H 5.03044700 1.10540000 -1.90429000
H 3.69088300 2.02004700 -2.69129400
H 4.28125900 2.49376700 -1.05571100

TS2_cy_exo_$^4C_1$

C -0.20702500 1.25197300 0.21040600
O -4.06619100 0.60691200 0.24587600
O -2.08148300 -1.50316100 1.14183800
O -2.47496100 -0.80464000 -1.85044300
O -1.60045600 1.14525300 0.40056200
Ru -2.54490600 -0.43221100 -0.21183500
H -3.72789900 1.50901600 0.40168700
C 0.29756000 -0.83047200 1.58715800
C 1.55296600 -1.94556500 -0.11057600
C 1.19289500 -2.04195400 1.39074300
H -0.95147400 -1.27598700 1.54076400
H 0.30683500 -0.29061400 2.54037100
H 1.25099700 -2.82598000 -0.68884700
H 0.68188000 -2.98179700 1.63646900
H 2.08675800 -1.96218000 2.02605500
N 0.43790100 -0.05105200 0.47123100
O 0.78898300 -0.83920100 -0.62989100
C 0.13393400 1.72467700 -1.21058300
C 0.30507700 2.26584700 1.24743400
H 0.07166300 1.90255700 2.25997300
H -0.29033200 3.17976000 1.09678900
C 1.79656400 2.56183300 1.07624100
H 2.11366900 3.29788900 1.83175100
H 2.37128300 1.64004200 1.26382400
C 2.09937600 3.06932500 -0.33689800
H 1.59805200 4.04241300 -0.48772500
H 3.18030300 3.24917300 -0.45431900
C 1.61297700 2.07797900 -1.39904200
C 3.03384200 -1.69099600 -0.37594000
O 3.73328100 -2.39569100 -1.06279000
O 3.44131700 -0.58082800 0.23455800
C 4.78977700 -0.15343700 -0.00453300
H 5.50090900 -0.90080800 0.37464000
H 4.95543100 -0.00557600 -1.08093700
H 4.90552400 0.79381700 0.53329000
H -0.49105700 2.61755300 -1.36870300
H -0.19764800 0.96690200 -1.93388300
H 1.75816600 2.49492700 -2.40859800
H 2.21589700 1.15913400 -1.34458600

TS2_cy_exo_$^1C_4$

C 0.08113500 0.86555500 0.27923800
O -3.90332800 0.43795000 0.34682400
O -2.22340300 -1.61036200 1.03198700
O -2.30914700 -0.61881500 -1.92874200
O -1.30121000 1.11970000 0.42756400
Ru -2.27614500 -0.36635200 -0.27661000
H -3.72576400 1.39038200 0.43455700
C 0.22831200 -1.49172200 1.30590300
C 1.80446300 -2.09944300 -0.32649700
C 1.13150000 -2.65129100 0.92459800

H -1.12394100 -1.84399600 1.36093100
H 0.34724800 -0.98921000 2.26904400
H 2.02515300 -2.84614300 -1.10042400
H 0.55338800 -3.55445100 0.68024700
H 1.85227100 -2.88909000 1.71580300
N 0.12487500 -0.65233500 0.20166700
O 0.80818800 -1.22248200 -0.89540400
C 0.60205000 1.45258300 -1.03556300
C 0.82816000 1.43322700 1.48893700
H 1.87284500 1.08512500 1.46101200
H 0.37293500 1.04234800 2.41056300
C 0.77846300 2.96616600 1.49642300
H -0.26395400 3.28353600 1.66394000
H 1.36738600 3.34049400 2.34833100
C 1.28516100 3.55859900 0.17970500
H 1.19792700 4.65668200 0.19863800
H 2.35921600 3.32642000 0.06200700
C 0.51030500 2.98045300 -1.00648500
H 0.88989700 3.38356100 -1.95820900
H -0.55007800 3.27422500 -0.93342700
H 1.64378600 1.13651100 -1.18129100
H 0.01747700 1.03594300 -1.86854400
C 3.07326300 -1.29592200 -0.01064900
O 3.57005300 -1.20907100 1.08924100
O 3.53591700 -0.69405600 -1.09828200
C 4.69262700 0.14340100 -0.93871900
H 4.46269900 0.98492900 -0.26945500
H 5.52835100 -0.43490200 -0.52119000
H 4.94023200 0.51092200 -1.94019200

TS2_cy_endo_$^{4}C_{1}$

C -1.54711000 -0.74969700 1.54722200
H -1.79461500 -1.81793100 1.57485100
H -1.33879000 -0.41482100 2.57548100
C -0.33016100 -0.46554600 0.67662900
H 2.45855600 -2.52637500 1.79721200
O 2.78933100 -2.67022900 0.89038900
O 3.20262200 -0.06666800 -0.48636300
O 0.63596200 -1.74652400 -1.14530500
O 0.86753600 -0.86202200 1.13209100
Ru 2.10649300 -1.33875200 -0.24412400
N -0.50466700 0.84295400 0.12823300
H -0.30076500 -1.23144200 -0.42635400
C -2.60965000 0.12260500 0.86856700
O -1.89000800 0.88166600 -0.14262800
H -3.11507800 0.80684300 1.56472200
C -0.04954800 1.99868600 0.97300400
H -0.19884900 1.74202700 2.03905800
C -0.87181700 3.25122000 0.64071800
H -0.55625700 4.02358900 1.36278100
H -1.94043900 3.07080700 0.81704100
C 1.43659500 2.27606700 0.71956800
H 1.74371000 3.04847100 1.44564500
H 2.04607200 1.38916100 0.93174500
C 1.69739000 2.76741000 -0.70705700
H 2.77192000 2.97512300 -0.83367400
H 1.45229900 1.95498500 -1.40994200
C -0.62884000 3.75494800 -0.78519800
C 0.86090600 4.00555900 -1.04267200
H 1.02302300 4.30570800 -2.09141000
H 1.19816000 4.85304600 -0.41786800
H -1.20990800 4.67619800 -0.95553700
H -1.00773300 3.00576400 -1.50015600
C -3.67782700 -0.69052800 0.15236100
O -4.85762100 -0.63419600 0.40485700
O -3.13791900 -1.49236300 -0.76669800
C -4.03347800 -2.34548600 -1.49140400
H -3.40974200 -2.92302000 -2.18223300
H -4.56453100 -3.01678600 -0.80134000
H -4.76804600 -1.74569800 -2.04736800

TS2_cy_endo_$^{1}C_{4}$

C 1.39779300 -0.77133200 1.61597600
H 2.19079500 -0.18104500 2.09047400
H 0.75425900 -1.19511800 2.40327800
C 0.54635100 0.03666000 0.65347500
H -0.91704600 3.17509100 1.94191000
O -0.80599800 3.62385500 1.08288700
O -1.92780700 1.95949400 -1.14191100
O 1.10707600 1.92339200 -0.76777700
O -0.20578500 1.05014900 1.10336900
Ru -0.53071700 2.33633600 -0.26578200
N -0.01441400 -0.89366200 -0.28098700
H 1.31006700 0.79213800 -0.13937500
C 1.91126400 -1.86503500 0.66876700
O 1.06867400 -1.78388100 -0.51890400
H 1.85593700 -2.87381200 1.09915500
C -1.20165600 -1.70478300 0.13028900

H -0.88166600 -2.38993500 0.94075300
C -1.63214900 -2.53850600 -1.08106900
H -0.78539300 -3.15113300 -1.42089800
H -1.87712500 -1.83766200 -1.89839400
C -2.36262200 -0.85388100 0.64531100
C -3.58941500 -1.72440900 0.94926300
C -2.84841000 -3.41279800 -0.76793700
C -4.01767400 -2.57207400 -0.25003600
H -4.37342200 -1.90728100 -1.05800600
H -4.86690200 -3.21917500 0.02432600
H -2.57428400 -4.16685700 -0.00798300
H -3.14241100 -3.97234800 -1.67075800
C 3.34890600 -1.63887300 0.21511700
O 4.24378500 -2.43464900 0.37119900
O 3.49170100 -0.44562300 -0.36341300
C 4.79711400 -0.10014600 -0.84384100
H 4.70395500 0.90235500 -1.27536500
H 5.52142800 -0.09714300 -0.01695500
H 5.12469400 -0.81847000 -1.60885800
H -2.07292900 -0.31455500 1.55559300
H -2.61244400 -0.09371400 -0.11359400
H -4.41619200 -1.07510100 1.27984000
H -3.35905200 -2.39021400 1.80071100

Psec_cy_exo_$^4C_1$

C -0.32897300 1.18076900 0.44693500
O -3.79576300 1.03223900 -0.49802700
O -2.61563300 -1.50154900 1.38267600
O -2.34971900 -1.37033100 -1.64165100
O -1.54180400 0.80453300 0.84036800
Ru -2.68804200 -0.49674600 -0.23890800
H -3.23496100 1.81048700 -0.33006800
C 0.91977400 -0.78683700 1.66834100
C 1.84102000 -1.91671700 -0.15076300
C 1.57364900 -2.08931200 1.36041900
H -1.88832500 -1.12548900 1.90850800
H 0.66429900 -0.38208200 2.64736300
H 1.55850300 -2.78752800 -0.75148700
H 0.86615400 -2.90750200 1.57582000
H 2.47882500 -2.26237600 1.95877500
N 0.62339600 -0.15353000 0.60068700
O 0.97372000 -0.82148300 -0.54243200
C -0.18057000 1.62074100 -1.01415900
C 0.27327900 2.20488700 1.41604100
H 0.22350500 1.81011900 2.44225100
H -0.42776500 3.05435400 1.38435200
C 1.67933900 2.67279900 1.03743200
H 2.00394100 3.44936100 1.74747800
H 2.39274800 1.83752500 1.13781100
C 1.71338300 3.20118400 -0.39917500
H 1.07884900 4.10292200 -0.46935500
H 2.73634200 3.51165900 -0.66602800
C 1.21270900 2.14177400 -1.38488900
C 3.30037800 -1.56601900 -0.44007600
O 4.10935900 -2.37609200 -0.81610200
O 3.55327200 -0.28871900 -0.16910400
C 4.90107800 0.17266100 -0.35798700
H 5.58512200 -0.37837100 0.30224400
H 5.20624800 0.03072300 -1.40371100
H 4.89127600 1.23716100 -0.10121600
H -0.92480800 2.42684200 -1.12445500
H -0.49898200 0.80469900 -1.67671300
H 1.17722600 2.55186100 -2.40657400
H 1.92401200 1.30278900 -1.40641800

Psec_cy_exo_$^1C_4$

C 0.07215000 0.91405200 0.02990700
O 1.84005200 -1.97813000 -1.55345000
O 1.74255100 -1.82514800 1.16949200
O 4.17200500 -0.23185400 -0.10322400
O 1.03449200 0.40660700 -0.77652900
Ru 2.58023100 -0.73628500 -0.21897400
H 1.48909300 -1.42407900 -2.27117100
C -0.77989900 -1.48053700 -0.01139000
C -2.79883600 -1.12551200 1.09526000
C -1.89115000 -2.26418500 0.59959300
H 1.44427900 -1.27508700 1.91011400
H 0.03561500 -1.81733800 -0.66857200
H -3.22869100 -1.30663900 2.08907700
H -1.48024600 -2.87978000 1.41669600
H -2.39572000 -2.93184500 -0.11092100
N -0.86417900 -0.25601900 0.35918200
O -1.91557800 0.00678200 1.21834600
C 0.56362600 1.49761300 1.36621500
C -0.77591600 1.94284500 -0.73944500
H -1.68294300 2.16823900 -0.15838700

H -1.08931900 1.47902100 -1.68679100
C 0.03984200 3.21452100 -0.98563800
H 0.88477900 2.97162900 -1.65068300
H -0.58462800 3.95127600 -1.51535900
C 0.57360100 3.80175300 0.32475100
H 1.19138500 4.69074100 0.11976800
H -0.27597900 4.14210700 0.94413800
C 1.38339300 2.76467400 1.10902600
H 1.71819800 3.18003500 2.07249200
H 2.29210200 2.49628800 0.54483400
H -0.30315000 1.72829800 2.00335100
H 1.16730900 0.74033700 1.88757800
C -3.94499000 -0.85147200 0.11388000
O -4.66179400 -1.73951300 -0.28033000
O -4.04599100 0.42644800 -0.21736600
C -5.09730900 0.77577400 -1.13327000
H -4.97469200 0.22777900 -2.07785200
H -6.07670600 0.53488500 -0.69704400
H -5.00457900 1.85408900 -1.29954800

3-Isoxazolidinone_cy_$^{4}C_{1}$

C 1.99649100 1.81050900 0.18862200
H 2.74167900 1.74941700 -0.61322700
H 2.25405100 2.64891700 0.85364100
N -0.21288000 1.12334000 0.26988600
C 1.81975600 0.50129700 0.95489700
O 0.45414500 0.50813000 1.35827900
H 2.41908400 0.43839800 1.87468900
C -1.68025600 1.12028600 0.26769300
C -2.18635600 0.70077500 -1.12379700
C -2.25638500 0.20494600 1.35636200
H -2.00002400 2.15809300 0.46646500
C -1.92660400 -0.78069000 -1.40601600
H -1.72100800 1.34263200 -1.88503200
H -3.27107900 0.90307600 -1.15097900
C -2.05496200 -1.28752400 1.06710800
H -1.83763600 0.47506800 2.33656100
H -3.33543100 0.42994400 1.39887300
C -2.55769700 -1.66982600 -0.32940300
H -0.83780900 -0.95783700 -1.43114000
H -2.31398400 -1.04370900 -2.40379600
H -3.65728900 -1.56065300 -0.36986900
H -2.33952500 -2.73149000 -0.53266000
H -0.98378200 -1.52527100 1.14272900
H -2.57531500 -1.87999900 1.83771900
C 2.17724400 -0.71107000 0.08331700
O 3.16637400 -0.74420400 -0.61107900
O 1.30951700 -1.70956400 0.20894900
C 1.56204600 -2.88946700 -0.56600400
H 2.51985500 -3.34276300 -0.27372400
H 0.73260700 -3.57240800 -0.35248400
H 1.58956700 -2.64329800 -1.63681200
C 0.58980700 2.02714300 -0.35867500
O 0.22092600 2.83191500 -1.20049600

3-Isoxazolidinone_cy_$^{1}C_{4}$

C 1.40205600 -0.45510300 -0.27972600
C -1.92883900 -0.65670800 0.99271100
C -1.99408100 -1.94009900 0.16467300
H -2.37485600 -0.75183000 1.99323500
H -1.98970200 -2.84190200 0.79573700
H -2.86027000 -1.97950800 -0.50610600
N 0.06003700 -0.90972000 0.00172800
O -0.53498400 -0.42213300 1.18291100
C 2.38589900 -0.85318100 0.83051500
C 1.43420200 1.05460000 -0.55471500
H 1.04483000 1.57747900 0.33405900
H 0.74817900 1.28638200 -1.38438300
C 2.85678300 1.52872300 -0.86574600
H 3.19111200 1.07536800 -1.81661900
H 2.86091500 2.61980200 -1.02076100
C 3.83552000 1.13981500 0.24718900
H 4.85745500 1.46094600 -0.01243900
H 3.56154000 1.67480400 1.17468000
C 3.80346400 -0.36951600 0.50900400
H 4.48405800 -0.63407400 1.33433400
H 4.17394500 -0.90104400 -0.38626900
H 2.04502600 -0.40335300 1.77823100
H 2.36314300 -1.94659500 0.96398600
C -2.63107900 0.50735500 0.28134400
O -3.73710500 0.40167700 -0.19601500
O -1.90934200 1.62220100 0.28073600
C -2.49434200 2.76720700 -0.35426600
H -2.71125200 2.54990200 -1.40979600
H -3.42652800 3.05318000 0.15348400
H -1.75275600 3.56941600 -0.27385300
H 1.66317100 -0.99887900 -1.20264400
C -0.68234300 -1.86431900 -0.61380500

O -0.34628300 -2.50108200 -1.60138900

TS1b_exo_cy_$^{4}C_{1}$

C 0.22781800 1.18976400 0.20063500
H -1.06113500 1.04185200 0.38661700
O -2.24004000 0.91907900 0.84650200
O -3.24553800 -1.71507400 0.62140100
O -4.33729900 0.47577300 -1.02712400
O -1.72150000 -0.38912800 -1.27972900
Ru -3.05536900 -0.24726100 -0.19284000
C 1.77276200 -1.91542600 -0.60034800
C 1.08256300 -2.36430900 0.68394000
H 1.61533800 -2.59545900 -1.44798900
H 0.18026700 -2.96004400 0.47716200
H 1.73117900 -2.92649700 1.36601200
N 0.80791400 -0.08254800 0.27365000
O 1.11881000 -0.68979900 -0.94011700
C 0.52355700 2.05422200 1.42943400
H -0.17443200 2.90515000 1.40718200
H 0.34205400 1.49266800 2.35228000
C 0.45682100 1.92965600 -1.10292300
C 1.97602000 2.56402000 1.35789000
H 2.16848900 3.20470600 2.23336300
H 2.66008500 1.70147600 1.43155900
C 2.23945800 3.32382000 0.05588000
H 3.29348400 3.64367300 0.01201100
H 1.62933000 4.24477800 0.04452400
C 1.89955500 2.47156400 -1.16945200
H 0.23180600 1.28365600 -1.95762800
H -0.25683400 2.76839100 -1.12250700
H 2.01582100 3.05597800 -2.09603500
H 2.58977100 1.61616100 -1.23415600
C 3.28403600 -1.75071800 -0.39982300
O 3.97101300 -2.65549700 0.01002100
O 3.72539800 -0.54249000 -0.72525000
C 5.13100800 -0.29885400 -0.55741000
H 5.71378600 -0.97588300 -1.19730500
H 5.29130100 0.74351300 -0.85274500
H 5.41963200 -0.45072800 0.49195100
C 0.64631900 -1.04267900 1.28343200
O 0.22308000 -0.81566600 2.39274500

TS1b_exo_cy_$^{1}C_{4}$

C 0.21398600 1.22235500 0.26335600
H -1.00970500 0.75632800 0.40817100
O -2.09868900 0.26453700 0.89584700
O -2.29058900 -2.52340100 0.36189500
O -4.04388200 -0.57581000 -0.99953100
O -1.29822200 -0.59053700 -1.34396400
Ru -2.57819400 -0.97851200 -0.25688700
C 2.36803400 -1.52598000 -0.42428600
C 1.83968800 -2.02452400 0.91438400
H 2.30912800 -2.27383700 -1.22719700
H 1.08378300 -2.81482400 0.79001900
H 2.62302200 -2.38342300 1.59227100
N 1.05655600 0.11495500 0.39683900
O 1.47093600 -0.47452600 -0.79462300
C 0.20093100 2.15591800 1.48035800
H 0.03915500 1.58285100 2.39967900
H 1.21488600 2.59414000 1.54298800
C 0.33169600 1.95682000 -1.06230400
C -0.84422400 3.26452200 1.32458100
H -0.75204000 3.95795400 2.17501100
C -0.73551400 3.04607900 -1.18754700
H 1.34040500 2.41245100 -1.08588800
H 0.27042700 1.24859800 -1.89334000
H -0.58725000 3.58655500 -2.13524600
C -0.70075600 4.01368400 -0.00213000
H -1.49827600 4.76742000 -0.10034000
H 0.25772800 4.56321000 -0.01124900
H -1.85101100 2.81891300 1.38881500
H -1.72800700 2.56678100 -1.24887200
C 3.82294000 -1.05035000 -0.33547100
O 4.68042800 -1.71006700 0.20229100
O 4.01620400 0.11736200 -0.93300200
C 5.35927400 0.62588300 -0.93253900
H 6.03246100 -0.07458400 -1.44655900
H 5.32003600 1.58175100 -1.46573000
H 5.71007600 0.77434700 0.09845200
C 1.14374800 -0.79583900 1.46262600
O 0.72269300 -0.60083100 2.57834000

P1b_exo_cy_$^{4}C_{1}$

C -0.21271300 1.43527300 0.04780100
O -3.91921200 0.09073900 -0.30213900

O -2.06992600 -1.50864000 1.10952000
O -1.89772600 -0.94468300 -1.95398700
O -1.61422600 1.12242100 0.05083600
Ru -2.18844000 -0.63139400 -0.32680600
H -3.81536700 1.04593100 -0.12326600
C 1.13672200 -1.86135200 0.46619700
C 1.00505400 -1.54374900 1.94957800
H 0.67400100 -2.81431700 0.17321100
H 0.14990000 -2.05905700 2.40810900
H 1.91307000 -1.77142200 2.52069100
N 0.53067100 0.32513100 0.62893100
O 0.38054500 -0.83433300 -0.18785300
C 0.26536900 1.67727900 -1.39167200
C -0.04456600 2.71104900 0.88218000
H -0.38198000 2.52646500 1.90799500
H -0.72521600 3.44677700 0.42654700
C 1.39654100 3.23092300 0.85056000
H 1.44545900 4.17723900 1.41233400
H 2.04849000 2.51792700 1.38118700
C 1.89802100 3.43165000 -0.58280100
C 1.71627200 2.16499200 -1.42414400
H 0.14188900 0.76392400 -1.98695600
H -0.40707600 2.43994500 -1.81443900
H 2.95705000 3.73591300 -0.57437600
H 1.33507300 4.25921400 -1.05052700
H 2.37007600 1.36274600 -1.04494100
H 2.01438700 2.34797500 -2.46862000
C 2.59422100 -1.87509400 -0.00776200
O 3.47985800 -2.37
703100 0.64245100
O 2.74453700 -1.31851600 -1.20247200
C 4.07211500 -1.31202000 -1.74946500
H 4.75562100 -0.76786700 -1.08281800
H 4.43516100 -2.34108800 -1.88065700
H 3.99626200 -0.80477000 -2.71713000
C 0.71568100 -0.04952000 1.95279000
O 0.69784300 0.69295300 2.91002100

P1b_exo_cy_$^{1}C_{4}$

C -0.58471800 1.15728300 0.34945800
O -3.22478100 -1.73390400 -0.26971400
O -0.74866900 -2.41507700 0.89990300
O -1.08378100 -1.48850500 -2.05076300
O -1.66950500 0.21523900 0.24663900
Ru -1.36531900 -1.52599000 -0.39373200
H -3.57428600 -0.87539500 0.04069200
C 2.20737400 -1.07612900 0.15607300
C 2.04907300 -1.14517400 1.66974900
H 2.26181700 -2.05926000 -0.33227800
H 1.58936500 -2.08907100 1.99328000
H 2.99173300 -0.99824300 2.21043600
N 0.63236300 0.45688700 0.72612300
O 0.99078800 -0.47128700 -0.29720700
C -0.37800300 1.84621700 -1.01004400
C -0.99194000 2.16982000 1.43041300
H -0.13145600 2.83604400 1.60111400
H -1.17980800 1.63540100 2.36824700
C -2.21729300 2.97997400 0.99796600
H -3.08663500 2.30535500 0.93140800
H -2.44661300 3.71799600 1.78266200
C -2.00139400 3.67106300 -0.34966600
H -2.91117500 4.21527100 -0.64996800
H -1.19929100 4.42464400 -0.24913200
C -1.60760200 2.65559300 -1.42461400
H -1.40112900 3.16052300 -2.38140900
H -2.44722200 1.96348400 -1.60512800
H 0.50312600 2.50039800 -0.91209400
H -0.13402500 1.09061100 -1.76777000
C 3.43430100 -0.26288800 -0.27006200
O 4.51209700 -0.40281300 0.25753900
O 3.17687400 0.55946500 -1.27785100
C 4.27480300 1.34770900 -1.76229600
H 5.07501000 0.69626900 -2.14095000
H 3.86881400 1.96605700 -2.56997400
H 4.67388800 1.98043200 -0.95702100
C 1.06804000 -0.01636000 1.95879700
O 0.75692200 0.42590500 3.04203000

Isoxazoline_*p*OMe

C 3.82219400 -0.99595900 -0.11987500
H 4.40396700 -0.41676800 0.60748600
H 4.51912700 -1.52593400 -0.78109100
C 2.82575900 -1.96852100 0.54035200
H 2.82476200 -2.94966500 0.02559300
N 1.54631900 -1.27569700 0.37519100
H 3.01608200 -2.12955600 1.61065800

C 2.87602100 -0.07121800 -0.92078500
O 1.65036900 -0.76423400 -0.98236700
H 3.20917500 0.09622100 -1.95698100
C 0.35775600 -2.09686900 0.43698800
H 0.40202000 -2.60781800 1.41446000
H 0.37232600 -2.88757200 -0.34115300
C -0.90976000 -1.27886700 0.32932200
C -2.02596200 -1.76756700 -0.35283100
C -1.00448500 -0.01348500 0.93365900
C -3.21736800 -1.03458500 -0.43456200
H -1.97424800 -2.74466100 -0.84153000
C -2.17565500 0.73123000 0.85726200
H -0.13279400 0.39110000 1.44979600
C -3.29708500 0.22609300 0.17358200
H -4.06433000 -1.45218500 -0.97804600
H -2.24910000 1.71791000 1.31985200
C 2.72064600 1.30305000 -0.25637700
O 3.60361000 1.86738800 0.34993100
O 1.51210600 1.82245800 -0.46556200
C 1.24769500 3.09918000 0.12270600
H 1.91582700 3.86620000 -0.29569300
H 0.20327600 3.33200800 -0.11402100
H 1.38966200 3.05768800 1.21243400
O -4.39655500 1.02225600 0.15114800
C -5.55383500 0.56872600 -0.52394200
H -6.30975700 1.35699300 -0.41489100
H -5.35989900 0.40182400 -1.59773000
H -5.94210100 -0.36610000 -0.08366400

TS1_endo_*p*OMe

C 1.31539000 2.16496700 0.59608500
H 1.10810700 3.06433400 1.19035500
H 2.35518700 2.20614300 0.25257100
C 1.03660000 0.87891100 1.36961600
H 2.04177400 0.11534300 1.28805200
O 3.21143700 -0.51607600 1.40731400
O 5.17652000 0.16572400 -0.49022400
O 3.89843800 -2.49354700 -0.50996300
O 2.46615300 -0.17410800 -1.03708800
Ru 3.83786500 -0.82963100 -0.20649800
N -0.08189000 0.32154200 0.77344900
H 0.95528300 0.93338600 2.46474300
C 0.31942300 2.04202000 -0.57803600
O -0.12736400 0.68537400 -0.55793200
H 0.78925800 2.20136400 -1.55786800
C -0.59426900 -1.00935000 1.00712100
H -0.39322600 -1.22296600 2.06807900
H -0.00709100 -1.72606700 0.40370700
C -2.06726300 -1.10365100 0.68861900
C -2.56636300 -2.10370500 -0.14660700
C -2.96855200 -0.17342900 1.23436700
C -3.93388000 -2.19787900 -0.43466500
H -1.88042300 -2.82960100 -0.59114700
C -4.32634600 -0.24746500 0.95414200
H -2.59377300 0.62697500 1.87638400
C -4.82377900 -1.26474100 0.11682300
H -4.28677900 -2.99245200 -1.09099000
H -5.03083600 0.47398100 1.37262600
C -0.84972000 3.02213500 -0.43329100
O -0.68446100 4.18809000 -0.15973900
O -2.02633500 2.45031900 -0.65173600
C -3.18945700 3.27618700 -0.50692900
H -3.19394000 4.06951000 -1.26799800
H -4.04858600 2.60965700 -0.63947300
H -3.21067700 3.73342500 0.49240200
O -6.16091700 -1.26002200 -0.09787400
C -6.72605900 -2.25686100 -0.92934400
H -7.80607700 -2.06460900 -0.95728900
H -6.32338200 -2.20538000 -1.95542800
H -6.54977400 -3.26893600 -0.52641200

P1_endo_*p*OMe

C 1.49922300 1.80162800 -0.16378700
H 1.61580400 2.55920400 0.62055500
H 2.38481600 1.81534900 -0.81059100
C 1.27997600 0.38079100 0.40134400
H 3.29606600 0.89705100 1.57743500
O 4.13421400 0.48228600 1.31533600
O 4.63933600 -0.17291200 -1.38760500
O 4.10031100 -2.30209000 0.78293500
O 2.08711600 -0.59284500 -0.26369100
Ru 3.93456600 -0.83326300 -0.01490500
N -0.10968100 0.14666200 0.11046200
H 1.40950200 0.29311500 1.49253600
C 0.20730200 2.03329800 -0.97274200
O -0.31888900 0.74106500 -1.18747700
H 0.39208100 2.48179900 -1.96038700
C -0.65639700 -1.19405000 0.13066200

H -0.25709500 -1.66119600 1.04678100
H -0.29341600 -1.79383000 -0.72205800
C -2.16841700 -1.16776700 0.16051200
C -2.92271400 -2.05500800 -0.60916900
C -2.85193100 -0.25604200 0.98326900
C -4.32349600 -2.05536600 -0.56742300
H -2.41426700 -2.76683000 -1.26545700
C -4.24015500 -0.23676200 1.03333100
H -2.27716500 0.45598000 1.57814300
C -4.99152500 -1.14014300 0.25829600
H -4.87547700 -2.76328500 -1.18480200
H -4.77349100 0.47455200 1.66743000
C -0.78135600 2.93016600 -0.21452800
O -0.44640200 3.82280000 0.53081200
O -2.04297900 2.63055500 -0.50704500
C -3.05880100 3.38677500 0.16049200
H -2.99643700 4.44875200 -0.11817000
H -4.01518000 2.96071900 -0.16212000
H -2.95125500 3.29432100 1.25075000
O -6.34043600 -1.04952400 0.37176500
C -7.15044200 -1.93521600 -0.37725500
H -8.19182700 -1.69165500 -0.13076000
H -6.99761800 -1.80709700 -1.46281500
H -6.95395900 -2.98849500 -0.11211900

TS2endo_*p*OMe

C -1.29048400 2.26655000 -0.08308100
H -1.48395700 2.18746500 -1.15868800
H -2.10258800 2.84006600 0.38782900
C -1.18853600 0.90431500 0.60776700
H -3.45704000 -1.65935700 1.72107000
O -3.54932200 -2.07655700 0.84350400
O -1.42884200 -0.87913500 -1.05367300
O -4.40295200 -0.08709700 -1.24779700
O -2.32330900 0.22254800 0.97968500
Ru -3.08280300 -0.79128900 -0.45792300
N -0.30330600 1.09618600 1.65372700
H -0.80066200 0.08498900 -0.28427400
C 0.07239400 2.87112200 0.25045500
O 0.43841300 2.27552800 1.51586500
H 0.05280800 3.95604900 0.40534600
C 0.39908300 0.02965400 2.35435600
H -0.36869200 -0.72861400 2.55927800
H 0.73409000 0.43888500 3.31819900
C 1.54758500 -0.54975500 1.56430400
C 2.81883200 0.03010900 1.61311500
C 1.34258100 -1.64345900 0.70707900
C 3.87273400 -0.45955700 0.83548800
H 2.99674200 0.88629500 2.26817300
C 2.37988600 -2.14462000 -0.07063500
H 0.35307100 -2.10103600 0.63786500
C 3.65497100 -1.55370600 -0.01653200
H 4.84997600 0.01739800 0.89908100
H 2.22697600 -2.99461300 -0.73840600
C 1.17629600 2.56962600 -0.76461700
O 1.99195800 3.38084100 -1.13133500
O 1.13985000 1.29889400 -1.16467200
C 2.15846300 0.86396300 -2.07558700
H 3.14988400 0.99121900 -1.62105600
H 1.96209900 -0.19704100 -2.25788900
H 2.10211200 1.43955400 -3.01003600
O 4.59989500 -2.09566900 -0.82067600
C 5.90162100 -1.53791000 -0.82353500
H 6.37361000 -1.60433900 0.17160100
H 6.49046100 -2.12419100 -1.54018300
H 5.88949300 -0.48214100 -1.14406300

P2endo_*p*OMe

C -1.43291000 2.29669000 -0.42081600
H -1.73931600 1.75333200 -1.32133800
H -2.22995900 3.00730200 -0.15040700
C -1.19009300 1.36956200 0.74698500
H -3.48668700 -0.88912400 2.06431000
O -3.53726500 -1.61177800 1.41346100
O -1.05590100 -1.57668600 -0.69020100
O -3.86165600 -0.78416800 -1.51477700
O -1.80768100 0.33107700 1.07940600
Ru -2.81022700 -0.98151700 -0.22846500
N -0.14532400 1.82512200 1.42513600
H -0.42115700 -0.84908600 -0.57089500
C -0.06856900 2.97318000 -0.53259900
O 0.45975900 2.92948500 0.82024600
H -0.09854200 4.02910400 -0.81997400
C 0.65648000 1.12474700 2.42469500
H -0.05298700 0.51256600 2.99551500
H 1.07589700 1.88864800 3.09304300
C 1.71798200 0.28374000 1.76230700

C 2.94360400 0.84869900 1.37330300
C 1.45441300 -1.04707800 1.42129600
C 3.87546900 0.10270400 0.66128600
H 3.16500400 1.88884700 1.62300100
C 2.38038100 -1.80934000 0.70365600
H 0.50247500 -1.50173300 1.70274700
C 3.59825100 -1.23195900 0.31208900
H 4.82816300 0.53523800 0.35023200
H 2.13557700 -2.83878300 0.44634800
C 0.91421200 2.25015200 -1.45926500
O 1.73850000 2.81331300 -2.13335800
O 0.75848800 0.92104500 -1.40849000
C 1.62851800 0.13076700 -2.23991800
H 2.67317700 0.27935000 -1.93953500
H 1.32878000 -0.90966200 -2.07945600
H 1.49689300 0.41505200 -3.29248900
O 4.54836800 -1.86990100 -0.40831400
C 4.31303400 -3.20302900 -0.82756900
H 5.19578700 -3.50868200 -1.40285600
H 4.18903700 -3.88221500 0.03279200
H 3.41960500 -3.27137400 -1.47103400

TS1_exo_proR_*p*OMe

O -0.19735200 1.30663400 -0.71434400
O 1.89419600 3.14501900 -1.11377500
O -0.75028100 3.99680500 -0.14146300
O 0.89642000 2.32002700 1.34755600
Ru 0.47809100 2.83826000 -0.24197200
C -2.31783800 -0.34602500 1.88206000
C -2.65399900 -0.54501100 -0.47206900
C -2.96455500 0.39813900 0.70708700
H -1.97067200 0.31188400 2.68751300
H -3.00428300 -1.09820600 2.29736400
H -2.29742700 0.00326400 -1.35437400
H -2.48929400 1.37287800 0.54180000
H -4.04085400 0.55466900 0.85413700
N -1.18568600 -1.03248000 1.27331100
O -1.59808700 -1.42167300 -0.01306100
C 0.08791000 -0.41925400 1.28532800
H 0.00745400 0.48783500 0.47590300
H 0.26102300 0.02798400 2.26837200
C 1.25823300 -1.19000600 0.78113100
C 2.52353800 -0.57894700 0.82694600
C 1.15763900 -2.48234500 0.23028300
C 3.66386400 -1.23052500 0.36229200
H 2.60921200 0.43361600 1.22786600
C 2.28753400 -3.14258300 -0.23255100
H 0.18673800 -2.97331900 0.17533600
C 3.55236500 -2.52530400 -0.17611300
H 4.62700600 -0.72493500 0.41712200
H 2.21991400 -4.15025800 -0.64693300
C -3.85953200 -1.37732200 -0.88142200
O -4.48728500 -1.18933300 -1.89676500
O -4.16577700 -2.29291600 0.03824300
C -5.32189500 -3.10156900 -0.21690700
H -5.19726100 -3.66341500 -1.15355400
H -5.40568600 -3.78870700 0.63216000
H -6.22083400 -2.47289000 -0.29168200
O 4.58850500 -3.24172300 -0.65815300
C 5.89012200 -2.67879600 -0.63890400
H 6.55955900 -3.43038700 -1.07515200
H 5.93885000 -1.75633100 -1.24143300
H 6.21732500 -2.45518400 0.39027200

TS1_exo_proS_*p*OMe

O -0.35691700 1.57841300 -1.48874500
O 0.06172500 4.08583300 -0.30364000
O -2.42050100 2.54108100 0.14794100
O 0.02580000 1.75376500 1.18503100
Ru -0.74948400 2.61041000 -0.12092500
C -0.36130100 -1.17000900 1.23175100
C -2.57873100 -1.01497900 0.43782100
C -1.78907800 -1.47714700 1.66972800
H -0.09427600 -0.10595900 1.42903700
H 0.40577300 -1.82089700 1.67327800
H -2.77239600 0.07207100 0.46001000
H -2.07865800 -0.93016600 2.57459500
H -1.93420700 -2.55455900 1.83251000
N -0.40843200 -1.35735000 -0.20274700
O -1.71659800 -1.31890500 -0.66779100
C 0.51923000 -0.83578300 -1.10207400
H 0.24579900 0.37994000 -1.21185500
C 1.96062200 -0.98303000 -0.73909200
C 2.57101400 -0.14826800 0.20764000
C 2.74520900 -1.96808800 -1.36990500
C 3.91648200 -0.30077700 0.54755200
H 1.98492400 0.64513200 0.67667100
C 4.08388100 -2.13108900 -1.03998700

H 2.29336000 -2.62211100 -2.11937100
C 4.68348100 -1.30036300 -0.07349500
H 4.35727200 0.36927300 1.28440600
H 4.69422600 -2.89961800 -1.51782000
H 0.27114900 -1.17437200 -2.11918700
C -3.91422900 -1.72121400 0.28105700
O -4.95342700 -1.22760900 0.64880800
O -3.78672100 -2.93949500 -0.23424400
C -4.98869900 -3.71416900 -0.35637700
H -5.71179800 -3.19912700 -1.00442000
H -4.68920100 -4.66960000 -0.80054100
H -5.44134700 -3.87543800 0.63237600
O 5.98910200 -1.52847700 0.18170600
C 6.65600000 -0.72674100 1.14245200
H 7.68978900 -1.09111500 1.18721700
H 6.65890300 0.33640300 0.84868700
H 6.19403200 -0.82338100 2.13937500

IPexo_proR_*p*OMe

O 1.27265900 -1.50197500 -1.43914500
O 1.11219000 -4.34593800 -0.63793300
O 2.70324700 -2.30660300 1.00788300
O -0.05054300 -2.08009700 0.55822800
Ru 1.47972300 -2.78810800 -0.07450300
C 2.07801000 0.86660800 1.09214100
C 1.99676400 2.07640200 -0.93729300
C 2.91347700 1.12769700 -0.15647900
H 2.24162400 -0.12776000 1.52373000
H 2.20108300 1.64801800 1.85561600
H 2.13255400 2.01153700 -2.02365800
H 3.04186500 0.19947700 -0.72757500
H 3.88989400 1.57250000 0.06746500
N 0.70524700 0.94692900 0.56067900
O 0.67386300 1.59191600 -0.66194000
C -0.36444200 0.38306100 1.03222000
H 0.40710500 -1.11206700 -1.18076900
H -0.17872400 -0.18679200 1.94112300
C -1.71584800 0.47173200 0.54951500
C -2.65486300 -0.38424400 1.16637500
C -2.16790400 1.34558600 -0.47126500
C -3.98771600 -0.39710300 0.78195300
H -2.31761800 -1.06787500 1.94695800
C -3.49770600 1.34673500 -0.84972800
H -1.47216500 2.02908300 -0.95105200
C -4.42258600 0.47335000 -0.23745700
H -4.68020200 -1.07878000 1.27292700
H -3.85940200 2.01971800 -1.62866800
C 2.17388700 3.53209900 -0.51068700
O 3.08227400 4.21430900 -0.91690400
O 1.25984100 3.91436400 0.37617200
C 1.37055800 5.25286700 0.88534800
H 1.28091500 5.97954500 0.06589600
H 0.54696500 5.37385600 1.59703200
H 2.33807700 5.38982300 1.38836700
O -5.68373900 0.54222100 -0.68286700
C -6.67706300 -0.30680600 -0.12101300
H -7.61233200 -0.07126300 -0.64246700
H -6.42838400 -1.36871000 -0.27828300
H -6.80152400 -0.11554800 0.95699000

IPexo_proS_*p*OMe

O -1.76009300 1.54641100 -1.14793700
O 0.76685300 2.80171800 -0.39686600
O -1.80840200 3.28910600 1.21242500
O -0.59105600 0.75427300 0.95923500
Ru -0.76914500 2.38930100 0.21602600
C -0.25374200 -2.88832900 0.58510500
C -2.32542500 -1.70813700 0.48782800
C -1.71785500 -2.98752300 1.04446600
H 0.43454000 -2.58649100 1.38534100
H 0.10574400 -3.81541000 0.12082600
H -2.10857700 -0.83015900 1.12378900
H -1.80005700 -3.03793900 2.13647000
H -2.21190700 -3.86597900 0.61069400
N -0.28654300 -1.80238200 -0.41421100
O -1.59310000 -1.50111700 -0.75136000
C 0.61518500 -0.92757200 -0.75621800
H -1.83894200 0.61127400 -0.86590300
C 2.02491800 -0.98769800 -0.51134800
C 2.74336500 -2.14400400 -0.11004400
C 2.73512000 0.22400900 -0.68399200
C 4.10282300 -2.07706600 0.11795000
H 2.24817900 -3.10850600 -0.01360000
C 4.09790100 0.30052800 -0.43569800
H 2.18890900 1.12554300 -0.97218900
C 4.79731000 -0.85421300 -0.03129700
H 4.66931700 -2.96208300 0.41063400
H 4.61011100 1.25327000 -0.55941300

H 0.21822500 -0.07870000 -1.31166500
C -3.80463100 -1.74904000 0.16584400
O -4.57309700 -2.56289800 0.61711700
O -4.13740400 -0.74004900 -0.63477500
C -5.52060600 -0.62478100 -1.00197700
H -6.14148300 -0.48301700 -0.10621200
H -5.58556000 0.25103200 -1.65625800
H -5.85062300 -1.52817900 -1.53392300
O 6.10821900 -0.89124700 0.22256700
C 6.88871000 0.29258700 0.09355600
H 7.91600600 0.01065800 0.35212700
H 6.86216900 0.67444200 -0.93938400
H 6.53832100 1.07554700 0.78470500

TSIP_proR_*p*OMe

O 0.88449000 -1.49797700 -1.49592800
O -0.15869900 -4.08100900 -0.70475900
O 2.36017800 -2.85940400 0.59974500
O -0.18090900 -1.79759700 0.92553900
Ru 0.84099800 -2.82267800 -0.15920600
C 2.21982300 0.53565500 1.38610600
C 2.39479100 1.43946900 -0.80045700
C 3.12741500 0.49259200 0.15790000
H 2.19109400 -0.41831300 1.92493200
H 2.47903700 1.35281300 2.07439100
H 2.55407300 1.18632500 -1.85579500
H 3.14889800 -0.51801700 -0.26828000
H 4.15094000 0.82150000 0.37266700
N 0.89642700 0.79868900 0.80126700
O 1.00764000 1.21970600 -0.53235500
C -0.25130700 0.29752500 1.20193900
H 0.35344700 -0.73171300 -1.20691000
H -0.21055400 -0.06656200 2.22669600
C -1.56163700 0.61410800 0.64723600
C -2.66313800 -0.10785400 1.14643300
C -1.80194700 1.63574600 -0.29847500
C -3.95881600 0.15237700 0.71582800
H -2.49041800 -0.90205100 1.87423700
C -3.09289600 1.91065200 -0.72310500
H -0.97443800 2.22770700 -0.68293200
C -4.18520100 1.17246300 -0.22790800
H -4.78320300 -0.43312300 1.12003600
H -3.28780200 2.70679500 -1.44359900
C 2.80358200 2.89400300 -0.58166700
O 3.81055100 3.35977300 -1.05803200
O 1.96601900 3.54139100 0.22374600
C 2.30143900 4.89832800 0.55225600
H 2.32444200 5.51564200 -0.35658000
H 1.51694600 5.24669100 1.23251600
H 3.28370900 4.93904500 1.04371800
O -5.39759400 1.51198300 -0.70092700
C -6.54742100 0.82229800 -0.23368600
H -7.40653600 1.27198700 -0.74613900
H -6.49892200 -0.25148300 -0.47882500
H -6.67111600 0.94069000 0.85544100

TSIP_proS_*p*OMe

O -1.64583000 1.50083200 -1.17321900
O 0.77211800 2.93132400 -0.38345700
O -1.80614300 2.95860500 1.32255100
O -0.22649900 0.65349800 0.84737300
Ru -0.67865200 2.29442000 0.23942000
C -0.33726700 -2.84038000 0.57471800
C -2.36694100 -1.60836200 0.45736200
C -1.80458500 -2.90112900 1.02513800
H 0.33811300 -2.49232200 1.36731200
H 0.02115900 -3.79912800 0.17939600
H -2.09557600 -0.73876100 1.08217000
H -1.89604300 -2.94684100 2.11667500
H -2.32184600 -3.76516600 0.58864600
N -0.34821300 -1.81957900 -0.49229700
O -1.65945000 -1.46580600 -0.80390200
C 0.55147800 -0.91325600 -0.78735400
H -1.71754500 0.54070700 -0.99871400
C 1.96247000 -0.98046200 -0.52495600
C 2.66489100 -2.13709800 -0.10496400
C 2.68627200 0.22021600 -0.70438900
C 4.02506000 -2.08284700 0.13293200
H 2.15869200 -3.09510800 -0.00087100
C 4.04860900 0.28569300 -0.44655400
H 2.15633000 1.12448200 -1.01196900
C 4.73339200 -0.87094400 -0.02420800
H 4.57869600 -2.97138600 0.43949900
H 4.57137500 1.23167000 -0.57828200
H 0.18593700 -0.10727400 -1.41888300
C -3.85623400 -1.58709400 0.17886300
O -4.64618200 -2.35026500 0.67899500

O -4.16752100 -0.59031500 -0.64446100
C -5.55523000 -0.42301700 -0.97266000
H -6.14010300 -0.22637800 -0.06312900
H -5.60207700 0.43490600 -1.65171700
H -5.94217000 -1.32597400 -1.46554600
O 6.04458200 -0.91820100 0.23935700
C 6.83459400 0.25712400 0.10292500
H 7.85834000 -0.02925000 0.37115800
H 6.81859700 0.62916200 -0.93406300
H 6.48667100 1.05121500 0.78289400

P1_exo_proR_*p*OMe

O 0.28761900 0.78845000 -1.47935300
O 1.80291500 2.84160700 -0.24566400
O -1.26785700 2.94626000 -0.65152200
O -0.00373400 1.30567000 1.30175500
Ru 0.22891400 2.25405100 -0.31649600
C -2.30966800 -0.41805000 2.01253700
C -2.65297700 -0.15562000 -0.31517400
C -3.02123000 0.50486300 1.02080400
H -2.04699200 0.07928400 2.95432000
H -2.92912500 -1.29879400 2.23518900
H -2.51379200 0.58197200 -1.11737100
H -2.61483000 1.52348200 1.05347900
H -4.10648100 0.55155500 1.18035900
N -1.09211900 -0.85896900 1.32899600
O -1.40312200 -0.82533600 -0.08881500
C 0.10988900 -0.10246500 1.57057900
H -0.05519300 -0.01324900 -1.02322300
H 0.22407600 -0.12122500 2.66858300
C 1.34127800 -0.73149900 0.94723000
C 2.56515100 -0.05591200 1.02017500
C 1.31140500 -1.99573600 0.33662200
C 3.73010600 -0.59674000 0.47180700
H 2.61718800 0.91908600 1.50730800
C 2.46331300 -2.54568200 -0.21537800
H 0.37282800 -2.54669000 0.28416000
C 3.68348000 -1.84951600 -0.16085500
H 4.66147200 -0.03663200 0.54388100
H 2.44226400 -3.52319700 -0.70099600
C -3.72401600 -1.13700100 -0.77370500
O -4.61574400 -0.82759000 -1.52923800
O -3.59707100 -2.33488100 -0.20443900
C -4.59945900 -3.30918300 -0.52392900
H -4.61162300 -3.50583600 -1.60537300
H -4.32715900 -4.21654800 0.02627000
H -5.59239700 -2.95471000 -0.21190700
O 4.74880700 -2.45791100 -0.73143500
C 6.00401200 -1.80158700 -0.71858900
H 6.70735100 -2.46378400 -1.23900100
H 5.96037900 -0.83387600 -1.24683400
H 6.36242500 -1.63168100 0.31097600

P1_exo_proS_*p*OMe

O 1.94072800 1.32569300 1.27188200
O 0.19283700 3.44204600 0.57821800
O 2.20973000 2.15155500 -1.37727800
O -0.19360300 0.83261400 -0.45592200
Ru 1.07236800 2.19997600 -0.13822000
C -0.05993600 -2.02225100 -1.04689400
C 2.20117200 -1.55116400 -0.63609700
C 1.33039600 -2.58726900 -1.32218500
H -0.32121400 -1.25851600 -1.79543200
H -0.83948800 -2.79526000 -1.03762900
H 2.31325000 -0.64380100 -1.25908100
H 1.54438000 -2.67891500 -2.39442100
H 1.47360200 -3.56730900 -0.84565300
N 0.05276900 -1.43002300 0.30489600
O 1.47611700 -1.24146400 0.56622000
C -0.59504000 -0.14882800 0.49463400
H 1.77887200 0.35173600 1.20703800
C -2.09892200 -0.33971400 0.40821900
C -2.73442600 -1.17389900 1.34396800
C -2.87203300 0.28776200 -0.56898600
C -4.10822100 -1.37096600 1.30010900
H -2.13954400 -1.67511100 2.11062200
C -4.25810100 0.09725300 -0.62519300
H -2.38851200 0.93877200 -1.29736000
C -4.88552400 -0.73530100 0.31267100
H -4.61055300 -2.01407700 2.02525900
H -4.83215600 0.60314700 -1.40052500
H -0.33024000 0.18614200 1.51013100
C 3.58968100 -2.01713500 -0.22709500
O 4.09593100 -3.05693000 -0.57410400
O 4.17816900 -1.10766100 0.54840000
C 5.49906900 -1.41048400 1.01888300
H 6.18451000 -1.55008600 0.17089200
H 5.80902500 -0.55168200 1.62403200

H 5.48799700 -2.32517200 1.62864200
O -6.21593600 -0.98275300 0.34930100
C -7.05476300 -0.37118900 -0.61388900
H -8.07681800 -0.70481700 -0.39436200
H -7.01214200 0.72944200 -0.54832200
H -6.78645900 -0.67895200 -1.63901700

TS2exo_proR_*p*OMe

O -0.88729300 -3.42568300 1.47962200
O -0.64001500 -2.41533600 -1.53672000
O -3.19790300 -2.10092200 0.11287200
O -0.41464000 -1.08132000 0.51257100
Ru -1.55991400 -2.50194100 -0.03005400
C -1.84276600 0.70259900 -1.43470500
C -1.47618400 1.92111600 0.60056100
C -2.57019200 1.11816700 -0.14195900
H -2.12426800 -0.28866400 -1.80612200
H -2.02751600 1.43522400 -2.23315300
H -1.23976300 1.47660500 1.57877900
H -2.88341400 0.24873100 0.44592500
H -3.45716800 1.72608000 -0.36413000
N -0.41994400 0.75073600 -1.11164800
O -0.31796100 1.89893100 -0.25515900
C 0.21559800 -0.36962300 -0.49526400
H -1.08556000 -4.37463700 1.39665400
H 0.06602600 -1.32368400 -1.39067800
C 1.65556800 -0.18021300 -0.23616900
C 2.40950700 0.80078600 -0.90938500
C 2.32517600 -1.04682100 0.65814800
C 3.77598500 0.93675800 -0.69033400
H 1.90896700 1.46502400 -1.61313500
C 3.68369300 -0.91641800 0.88767400
H 1.75306400 -1.81708800 1.17555600
C 4.42680000 0.07930100 0.21856100
H 4.32815700 1.70709200 -1.22688400
H 4.20698400 -1.57530500 1.58278400
C -1.86884100 3.37081700 0.83253400
O -2.11901400 3.83559800 1.92024000
O -1.95090600 4.04964000 -0.31281500
C -2.36950700 5.41672700 -0.22275000
H -1.67564900 5.99155500 0.40733600
H -2.36422500 5.80482400 -1.24743000
H -3.38032800 5.48240000 0.20542400
O 5.73887300 0.13179400 0.50306500
C 6.55436400 1.10975500 -0.12623400
H 6.21475700 2.12999700 0.11636600
H 7.56866500 0.96280600 0.26402700
H 6.56275100 0.97996600 -1.22087800

TS2exo_proS_*p*OMe

O 0.81578100 3.75667500 -0.81462700
O 0.67043900 1.46612300 1.32036100
O -1.84880000 2.84095100 0.24439500
O 0.25643300 1.22965200 -1.11223600
Ru -0.18731300 2.52348100 0.20517800
C 1.16854900 -2.33142500 -1.24860500
C 3.35013600 -1.43555600 -1.08061300
C 2.61324000 -2.66695200 -1.62288600
H 0.43284500 -2.73448400 -1.95769700
H 0.92088200 -2.70507400 -0.24367400
H 4.29891700 -1.24181200 -1.59886000
H 2.74305300 -2.71730400 -2.71236800
H 2.96098300 -3.60645000 -1.17641600
N 1.15332900 -0.86504100 -1.28768800
O 2.47458700 -0.35822600 -1.38349800
C 0.28985500 -0.04660200 -0.57384200
H 0.99503000 3.35375100 -1.68488600
C -1.04362200 -0.65290500 -0.24695300
C -1.21351000 -1.47015000 0.88417800
C -2.15209500 -0.37665300 -1.05569300
C -2.45372700 -2.01689600 1.18068200
H -0.36740200 -1.66551000 1.54673700
C -3.40647600 -0.91839700 -0.76720600
H -2.03000100 0.27178900 -1.92499200
C -3.56348400 -1.74888200 0.35463700
H -2.59756400 -2.65083700 2.05728700
H -4.24874300 -0.68727900 -1.41793100
H 0.75598500 0.31476700 0.56585700
C 3.65115500 -1.57583400 0.41251200
O 4.53617900 -2.28114800 0.83515300
O 2.81296000 -0.87069700 1.16879300
C 2.98019700 -0.94428200 2.59163300
H 2.82690700 -1.97521900 2.94078500
H 3.98827100 -0.61065900 2.87451600
H 2.22087500 -0.27597200 3.01151700
O -4.72907900 -2.32243500 0.72083700
C -5.89051200 -2.08660300 -0.05745200
H -6.70434400 -2.64407500 0.42253100

H -6.14979800 -1.01479300 -0.08071800

P2exo_*p*OMe

O 3.45652500 -0.80374400 1.17273600
O 2.12981800 -0.06454100 -1.57211600
O 3.25027300 -2.89268400 -1.13364700
O 0.85591200 -1.31058200 0.63340400
Ru 2.65009400 -1.50385100 -0.42426900
C -1.57455600 -2.46710400 1.10460300
C -3.52390000 -1.08312300 1.09146900
C -3.05906600 -2.48687100 1.49821100
H -0.91862500 -2.90373000 1.87034600
H -1.37142400 -2.96708500 0.14668300
H -4.33343000 -0.69744600 1.72680100
H -3.17514100 -2.61372500 2.58186500
H -3.62745600 -3.27059800 0.98485000
N -1.29799000 -1.03261600 0.95895900
O -2.39281200 -0.25438500 1.34234100
C -0.10291800 -0.48531700 0.71457900
H 4.30492600 -0.38316800 0.95974500
H 1.16120200 0.00956400 -1.59765300
C 0.06310800 0.97583400 0.58499500
C -0.91398900 1.80459300 0.00886700
C 1.27978200 1.54857300 1.01314100
C -0.69005300 3.17172500 -0.14378600
H -1.85247300 1.37691400 -0.33784300
C 1.49776000 2.91019600 0.88378100
H 2.05634300 0.90723200 1.43340900
C 0.51780400 3.73790000 0.30092100
H -1.46038700 3.78542100 -0.60847600
H 2.43094800 3.36595900 1.21964000
C -4.01126100 -1.03874300 -0.36053400
O -4.85741300 -1.79328100 -0.77685200
O -3.42130300 -0.08914200 -1.08008200
C -3.82110300 0.02778000 -2.45456300
H -4.88813400 0.28243200 -2.51981200
H -3.20762100 0.83040100 -2.87775400
H -3.63958400 -0.91747500 -2.98454500
O 0.82250900 5.04615400 0.20752300
C -0.11312500 5.93915900 -0.37655500
H -0.32441400 5.67111400 -1.42513600
H -1.05908500 5.95769300 0.18975100
H 0.34721100 6.93425500 -0.34396800

3-isoxazolidinone_*p*OMe

C 3.02171400 -0.46451300 -0.18378000
C 2.61577700 -1.01286200 1.19064500
H 3.87276800 -1.02498400 -0.60201100
H 3.41757700 -1.56457200 1.69641800
H 2.25098700 -0.22469100 1.86805400
N 1.13579500 -1.70444700 -0.45190000
O 1.86926500 -0.63971900 -1.02052300
C -0.13532500 -1.96147700 -1.08811600
H -0.40412900 -2.99649600 -0.83395900
C -1.25074800 -1.01866000 -0.67382800
C -2.56824600 -1.48287100 -0.61939000
C -1.00541000 0.32866200 -0.35406300
C -3.62883000 -0.64179700 -0.26444500
H -2.78287100 -2.52938200 -0.85367900
C -2.04905600 1.17441800 0.00884200
H 0.01269400 0.71969300 -0.38481300
C -3.37190800 0.70006000 0.05406500
H -4.64040700 -1.04481400 -0.23102200
H -1.86187800 2.21985900 0.26297000
H 0.04148300 -1.93027500 -2.17501000
C 3.40387000 1.00718500 -0.15005600
O 4.54604400 1.39699000 -0.18985000
O 2.33761400 1.79373200 -0.01085800
C 2.58065700 3.20339500 0.09539200
H 3.09803500 3.57111900 -0.80192500
H 1.59551900 3.67313400 0.18869500
H 3.19474600 3.41957700 0.98114500
C 1.43282100 -1.91211900 0.86099100
O 0.83290300 -2.67126600 1.60198200
O -4.32167300 1.59769100 0.41627600
C -5.67115700 1.17655700 0.48285200
H -5.81009000 0.37260000 1.22613700
H -6.25761600 2.05239700 0.78842200
H -6.03555100 0.82266500 -0.49698500

TS1b_exo_proR_*p*OMe

O -1.95254000 -0.16760300 -1.10550100
O -4.17996900 1.54351900 -0.70352800
O -4.37622000 -1.35142400 -0.29592500
O -2.76553900 0.26117900 1.29455100
Ru -3.46990300 0.07298600 -0.26645700
C 1.75179700 -2.85692600 -0.89564900

C 0.86246900 -3.68268900 0.03205000
H 1.75658300 -3.21297000 -1.93501000
H 0.03737900 -4.16829900 -0.51112400
H 1.41182600 -4.44475500 0.59715000
N 0.60451300 -1.39468000 0.36172700
O 1.14240300 -1.56015800 -0.92075000
C -0.02883700 -0.18343900 0.65693700
H -0.97286700 -0.12234600 -0.18880900
H -0.51871300 -0.30802700 1.62445500
C 0.66340400 1.10790100 0.47097800
C -0.00866900 2.27505900 0.90157100
C 1.92822700 1.23989200 -0.12807600
C 0.58107100 3.52041800 0.77538800
H -1.00665100 2.18292900 1.33643300
C 2.53315900 2.48954000 -0.24975100
H 2.45693700 0.35553900 -0.47999400
C 1.86423400 3.64284300 0.20041600
H 0.07225300 4.42574600 1.11073900
H 3.52484300 2.55538800 -0.69549000
C 3.20537900 -2.82141400 -0.40493500
O 3.78553700 -3.81670400 -0.04267300
O 3.73635500 -1.60596300 -0.45596300
C 5.09745900 -1.47302200 -0.01629200
H 5.76467400 -2.06280400 -0.66019600
H 5.33391300 -0.40621200 -0.09117800
H 5.19589600 -1.81763400 1.02248200
C 0.27778500 -2.61883700 0.94013200
O -0.35097300 -2.73297300 1.96818100
O 2.36205800 4.88995800 0.11952500
C 3.64605900 5.09375100 -0.45119000
H 3.67558400 4.76091400 -1.50178600
H 3.83571800 6.17307700 -0.40768600
H 4.42666600 4.56373800 0.11920400

TS1b_exo_proS_*p*OMe

O -0.27482400 1.65541000 -1.48710500
O -0.37699100 4.10561700 -0.01031300
O -2.55197300 2.13911500 0.08298900
O -0.01701700 1.64041900 1.13347100
Ru -0.90570700 2.50128200 -0.07466100
C -2.57728200 -1.21872900 0.11607000
C -1.95359700 -1.75023400 1.40481900
H -2.87564600 -0.16091900 0.23523500
H -2.33583100 -1.26877700 2.31218000
H -2.06512900 -2.84142200 1.50949600
N -0.32583700 -1.15798000 -0.14667300
O -1.51588600 -1.29815000 -0.85313900
C 0.71992600 -0.63887600 -0.92074700
H 0.38053700 0.60453400 -1.15094700
C 2.12995800 -0.68428600 -0.49435900
C 2.61694700 -0.07518700 0.68390600
C 3.05722400 -1.30022200 -1.35738600
C 3.95856600 -0.14345400 1.01017400
H 1.92164300 0.47311100 1.31702500
C 4.41015900 -1.38153800 -1.03685400
H 2.70864300 -1.74936900 -2.29018800
C 4.87155100 -0.80817700 0.16184500
H 4.34382700 0.32890300 1.91551400
H 5.09135200 -1.88695800 -1.71973100
H 0.57145600 -0.98346300 -1.95398400
C -3.76116000 -2.00554100 -0.41196100
O -3.79685600 -2.59667900 -1.46084100
O -4.75912400 -1.93312000 0.46763900
C -5.97151400 -2.62382400 0.13102000
H -5.77856100 -3.69912600 0.01020500
H -6.66005100 -2.45030000 0.96499900
H -6.39174400 -2.22399400 -0.80269200
C -0.48279700 -1.42521300 1.20927000
O 0.40246800 -1.41164700 2.03191800
O 6.15240500 -0.82367500 0.56623100
C 7.13434400 -1.45552500 -0.24276200
H 8.08839200 -1.34378200 0.28631500
H 6.91404200 -2.52759500 -0.37460000
H 7.20746700 -0.97487000 -1.23205600

P1b_ exo _proS_*p*OMe

O -1.92096300 1.50219300 -1.23419700
O -0.14936500 3.60955300 -0.58701800
O -2.14722500 2.37476100 1.40946400
O 0.25890200 1.02629000 0.48630500
Ru -1.02295700 2.38307600 0.16008000
C -2.39189200 -1.51464500 0.61328500
C -1.53352200 -2.48965900 1.39405300
H -2.63012800 -0.61596100 1.21032600
H -1.74001600 -2.50594900 2.47029200
H -1.62363900 -3.51348800 0.99856200
N -0.20479600 -1.22498200 -0.05261900
O -1.54681500 -1.12673300 -0.50275200

C 0.56376700 -0.04177900 -0.38792100
H -1.79267500 0.52856500 -1.17251300
C 2.05381800 -0.32853400 -0.36213100
C 2.56307500 -1.38267300 -1.13799900
C 2.93905000 0.46818300 0.36185200
C 3.92797600 -1.63244000 -1.18162000
H 1.88007100 -2.01545200 -1.70939800
C 4.31772900 0.22735600 0.32588700
H 2.55190200 1.28993800 0.96403400
C 4.82133000 -0.82680800 -0.44949300
H 4.33375300 -2.44878200 -1.78212400
H 4.98319900 0.86815100 0.90301800
H 0.27282000 0.21183300 -1.42003500
C -3.68250600 -2.07147700 0.03310100
O -4.14555200 -3.14915700 0.31571600
O -4.22787800 -1.19653300 -0.80546900
C -5.45635600 -1.58258700 -1.44144800
H -6.23232000 -1.77057300 -0.68609900
H -5.74110000 -0.74213500 -2.08321300
H -5.30579000 -2.49121900 -2.04122600
C -0.13366500 -1.95198700 1.13109600
O 0.86796800 -2.11194800 1.78887100
O 6.13391600 -1.14000000 -0.55798900
C 7.08593900 -0.35661500 0.13858600
H 8.07075400 -0.77862500 -0.09837200
H 7.05732000 0.69796800 -0.18504000
H 6.92716400 -0.40018500 1.22964300

P1b_ exo _proR_*p*OMe

O 0.07055300 -1.31032300 -1.70363400
O -0.51682600 -3.70741700 -0.37670500
O 2.37041200 -2.75135100 -0.97524100
O 0.73396600 -1.65074700 1.09848700
Ru 0.74974900 -2.60487700 -0.53909700
C 2.32408000 1.76426400 -0.65799400
C 3.32176700 1.44327800 0.45094700
H 2.70703700 1.54685600 -1.66477100
H 4.04929700 0.67878500 0.13850200
H 3.86668900 2.32183100 0.81582200
N 1.18736800 0.66620800 0.95486700
O 1.23973200 0.85749700 -0.44785600
C 0.27201200 -0.34264200 1.38627000
H 0.09893700 -0.41583500 -1.30700200
H 0.36216000 -0.29201000 2.48579700
C -1.15774000 -0.09330200 0.96191600
C -2.09708400 -1.13190100 1.09390700
C -1.59741600 1.15551400 0.51241600
C -3.42977900 -0.92746900 0.77054700
H -1.77937200 -2.11161300 1.45547500
C -2.93770000 1.37343600 0.18536800
H -0.89083000 1.97751000 0.39405200
C -3.87020700 0.33191300 0.31321600
H -4.16592900 -1.72766900 0.86905100
H -3.24091700 2.35836600 -0.17085900
C 1.88942200 3.23428800 -0.63873700
O 2.67081300 4.13058500 -0.42038100
O 0.60155800 3.39501900 -0.92035900
C 0.11507500 4.74489000 -0.98442700
H 0.63376400 5.29738400 -1.78046900
H -0.95494500 4.66760000 -1.20635600
H 0.27529300 5.25378100 -0.02368900
C 2.44121700 0.83722100 1.52621000
O 2.71758200 0.56237800 2.67297700
O -5.18043800 0.44875200 0.02014200
C -5.67907100 1.69841400 -0.41322300
H -6.75744000 1.56961700 -0.56861300
H -5.51791800 2.48607300 0.34368400
H -5.21264300 2.01788300 -1.36235200

TS2b_ exo_proR_*p*OMe

O 0.69781200 -3.51881400 -1.65905300
O 0.32234600 -2.47434100 1.30819700
O 2.75130500 -1.72246100 -0.37086600
O -0.15201800 -1.23651900 -0.75570300
Ru 1.22257100 -2.42570700 -0.20669900
C 2.42277200 1.46582400 0.38361000
C 2.14780000 1.54616100 1.87925700
H 3.08264900 0.60962200 0.14755000
H 2.98250100 1.20568700 2.50346100
H 1.86741500 2.56361700 2.19349900
N 0.46499500 0.43189500 0.75997300
O 1.12933300 1.20944500 -0.20864900
C -0.55171200 -0.44452400 0.27933800
H 1.09209400 -4.40257800 -1.55535000
H -0.45631000 -1.40931800 1.20098000
C -1.91015400 0.10799700 0.14957100
C -2.22354700 1.41819800 0.56811800
C -2.93368100 -0.69814000 -0.38226800

C -3.51363600 1.90545200 0.44535600
H -1.43987000 2.05852500 0.97513800
C -4.23081000 -0.21603000 -0.51785200
H -2.70182000 -1.71726500 -0.69637400
C -4.53231900 1.09610000 -0.10131600
H -3.76769800 2.91975200 0.75739300
H -5.00010600 -0.86089000 -0.93995200
C 3.00399600 2.72141400 -0.24011600
O 3.58005000 3.57621200 0.38962100
O 2.83285600 2.73032100 -1.55776100
C 3.35959300 3.86049300 -2.26860900
H 4.44837700 3.92500500 -2.13150800
H 3.11610100 3.69495100 -3.32360200
H 2.89493100 4.78935200 -1.90853000
C 0.95419800 0.61545000 2.02967900
O 0.49706300 0.09908200 3.02871900
O -5.74970400 1.65524600 -0.18652400
C -6.83465900 0.89573700 -0.70080000
H -7.71379400 1.54989500 -0.66239600
H -6.65174200 0.59281300 -1.74472200
H -7.02021000 -0.00150500 -0.08828700

TS2b_ exo_proS_*p*OMe

O 1.02043800 4.01245400 -0.49211800
O 0.61311100 1.57362700 1.43578300
O -1.75692200 3.09744900 0.25104300
O 0.36553000 1.54263300 -1.02607100
Ru -0.10503600 2.76045500 0.35088100
C 3.34938600 -1.39592500 -0.67944100
C 2.71992500 -2.31767400 -1.72902100
H 4.40711200 -1.17668900 -0.87158400
H 3.20629200 -2.20842800 -2.71045600
H 2.71878100 -3.38063900 -1.45632100
N 1.31804600 -0.55806900 -1.14885100
O 2.63106000 -0.16820900 -0.83271200
C 0.31189700 0.23919400 -0.58805800
H 1.26789100 3.66971000 -1.37174800
C -1.02677200 -0.38434000 -0.43809700
C -1.18173300 -1.55608400 0.31969100
C -2.16852900 0.22606900 -0.99008200
C -2.43619600 -2.12214100 0.51904600
H -0.30494000 -2.02560800 0.76993700
C -3.42547300 -0.33131300 -0.79709200
H -2.06017900 1.13469600 -1.58202700
C -3.57431300 -1.50795500 -0.04067800
H -2.52348700 -3.02958700 1.11461500
H -4.31697900 0.12684400 -1.22871400
H 0.66099500 0.50786400 0.64049200
C 3.23440200 -1.97071600 0.73582200
O 3.91690300 -2.89086800 1.11564400
O 2.30185300 -1.36113000 1.46476400
C 2.11670200 -1.82801900 2.80992400
H 1.82361100 -2.88727500 2.81085400
H 3.04470400 -1.70587500 3.38580200
H 1.31878300 -1.20705900 3.23062700
C 1.29515300 -1.78385800 -1.80656400
O 0.32404700 -2.29881800 -2.30853000
O -4.83299800 -1.97366100 0.09765700
C -5.06215800 -3.14725600 0.86060600
H -4.73815700 -3.01661400 1.90657200
H -6.14439600 -3.32459100 0.83792200
H -4.54270700 -4.01801800 0.42718500

1′_isoxazolidinone_*p*OMe

C -3.63397500 -0.97087500 0.82398400
H -4.54274500 -0.65883700 0.29676800
H -3.92071200 -1.45416200 1.76600300
C -2.73340900 -1.89764800 -0.01668700
H -2.65737200 -2.91109200 0.40820700
N -1.43604000 -1.21957700 0.01965200
H -3.05985200 -1.98837900 -1.06129200
C -2.71487600 0.22653400 1.10873300
O -1.40647000 -0.31919000 1.10973300
H -2.87165600 0.66159900 2.10700300
C 1.02300700 -1.09304600 -0.21330100
C 2.18326400 -1.88528100 -0.31771400
C 1.17028500 0.28221400 0.02306500
C 3.44477100 -1.32999200 -0.16571300
H 2.07306400 -2.95124900 -0.52283600
C 2.43617300 0.85519500 0.16033700
H 0.29069200 0.91911200 0.08978000
C 3.58473100 0.05064800 0.07495300
H 4.34643600 -1.94166000 -0.23314500
H 2.51534200 1.92807300 0.33148500
C -2.89580000 1.35242600 0.08248600
O -3.97391600 1.68419300 -0.35164200
O -1.74533100 1.94326300 -0.23293900
C -1.80841100 3.02559400 -1.17204800

H -2.41421200 3.84865900 -0.76717800
H -0.77382100 3.35098500 -1.32658100
H -2.24935300 2.68438500 -2.11924900
C -0.28051100 -1.77785800 -0.45264000
O -0.36101000 -2.82548400 -1.09651500
O 4.84778300 0.50904200 0.20540500
C 5.06118500 1.88972000 0.44636200
H 6.14704700 2.02817800 0.51962900
H 4.67214800 2.50869800 -0.37955200
H 4.59021800 2.21234800 1.39015100

TS1b_endo_*p*OMe

C 1.50586000 2.28198000 0.79118200
H 1.36542000 3.13241000 1.47319300
H 2.52846800 2.32322300 0.40030300
C 1.21687500 0.96515500 1.48517800
H 2.16118200 0.06801700 1.27243500
O 3.11144000 -0.74476300 1.21382800
O 4.94644100 -0.38101800 -0.91502800
O 3.01774400 -2.60207500 -0.93778000
O 2.20497700 0.05200500 -1.03189000
Ru 3.44485000 -1.02975000 -0.49715600
N -0.00245600 0.53447900 0.99518700
H 1.28447200 0.90303000 2.57855900
C 0.44528300 2.30109300 -0.33042300
O -0.23058700 1.03403000 -0.26827300
H 0.89626000 2.34870700 -1.33137600
C -1.97569300 -0.97074200 0.81456400
C -2.42210300 -2.29519800 1.01819400
C -2.80771300 -0.07973300 0.11277400
C -3.63970900 -2.72058600 0.51824800
H -1.78637400 -2.98418600 1.57603500
C -4.04295000 -0.49536300 -0.37986200
H -2.50262100 0.95201200 -0.04702400
C -4.46710900 -1.82255300 -0.18843700
H -3.98476800 -3.74622700 0.65937700
H -4.66807100 0.22173000 -0.90997100
C -0.52359100 3.47709000 -0.19700100
O -0.15051600 4.58795100 0.09528500
O -1.77918900 3.14498700 -0.47602300
C -2.75701900 4.19568200 -0.41541500
H -2.51810300 4.97931400 -1.14785300
H -3.71726200 3.72602200 -0.65396900
H -2.78224100 4.63468500 0.59164800
C -0.68974200 -0.61000900 1.44247700
O -0.17067800 -1.23505100 2.35519000
O -5.63364700 -2.31779100 -0.63556500
C -6.52044400 -1.47471900 -1.35671500
H -7.38877300 -2.09170900 -1.61768900
H -6.85164300 -0.62183200 -0.74168200
H -6.05168600 -1.09632800 -2.27995000

P1b_endo_*p*OMe

C -2.39125500 -1.27687000 -1.30056900
H -2.55608900 -0.78159500 -2.26418400
H -2.38678700 -2.36282500 -1.44859900
C -1.05354500 -0.85677500 -0.65081100
H 1.44728200 0.04479500 -0.79448300
O 2.13908800 -0.63046000 -0.67664800
O 1.93429300 -3.32534000 -1.32268500
O 1.80216200 -2.30026600 1.58023600
O -0.36244300 -2.02924900 -0.27223500
Ru 1.50233800 -2.29016800 -0.07020700
N -1.51270400 -0.10222300 0.50735000
H -0.45303700 -0.22027800 -1.30953700
C -3.43262600 -0.84820900 -0.24896200
O -2.69527900 -0.72095800 0.95762700
H -4.20375600 -1.61205700 -0.07331900
C 0.36819700 1.35317100 0.93435700
C 1.52720800 1.42263600 1.72943800
C 0.35091700 2.02252900 -0.29812400
C 2.65294500 2.09188300 1.27745400
H 1.53872900 0.91708700 2.69627800
C 1.47711100 2.70765300 -0.76129700
H -0.55909500 2.03129800 -0.90124000
C 2.64557900 2.73062800 0.02055700
H 3.56736100 2.12799000 1.87193300
H 1.43087700 3.21984500 -1.72094700
C -4.13852200 0.45388400 -0.64537400
O -4.56818000 0.64943400 -1.75872900
O -4.24139300 1.30751900 0.36723700
C -4.89350200 2.55516100 0.09347500
H -5.93357700 2.38505800 -0.21926300
H -4.86380600 3.12395500 1.02930300
H -4.36243700 3.09776000 -0.70164900
C -0.81425300 0.61422100 1.46033100
O -1.16230700 0.65549500 2.62795000
O 3.79012100 3.33530300 -0.34597100

C 3.86931500 3.96188300 -1.61755600
H 4.88973200 4.35405900 -1.70584000
H 3.68500400 3.24059100 -2.43100400
H 3.15171900 4.79468100 -1.70155000

TS2b_endo_*p*OMe

C 1.57006200 1.64323500 -1.13783500
H 2.24360200 2.31527800 -0.59479100
H 1.98513700 1.43642200 -2.13467700
C 1.32202300 0.30979900 -0.43206800
H 2.93171800 -2.88943400 -0.49984000
O 3.63439700 -2.64560500 0.13242500
O 2.85792000 0.09933800 1.44070000
O 5.04950800 -0.15756000 -0.71949800
O 2.08934700 -0.76741900 -0.72525800
Ru 3.75766000 -0.76786900 0.17933100
N -0.06356500 0.09325600 -0.52255300
H 1.79058500 0.42331200 0.83618800
C 0.15199600 2.20113100 -1.23158600
O -0.68306000 1.04466800 -1.34153700
H -0.02178300 2.79360700 -2.14107100
C -2.26015000 -0.96915700 0.04267600
C -3.12554000 0.09692400 -0.25690600
C -2.82667700 -2.22811100 0.34278000
C -4.50864400 -0.08518300 -0.27032700
H -2.72544600 1.08682200 -0.45483300
C -4.19655000 -2.42246500 0.31431400
H -2.16201100 -3.05478200 0.59648500
C -5.05673400 -1.34924900 0.00551700
H -5.14980500 0.76647500 -0.49356700
H -4.63569700 -3.39795900 0.53055300
C -0.20383300 3.07488200 -0.02121900
O 0.57637500 3.85183800 0.47472300
O -1.45950300 2.89906400 0.37121900
C -1.91565000 3.69120500 1.47870300
H -1.88378600 4.75893900 1.22010600
H -2.94578300 3.37246700 1.67035200
H -1.28537600 3.51073000 2.36041600
C -0.78196500 -0.89030200 0.16739900
O -0.13695600 -1.68210700 0.83895800
O -6.37420600 -1.62459800 0.00330300
C -7.30041700 -0.59296200 -0.30059400
H -8.29745500 -1.04709000 -0.24779400
H -7.23758000 0.23136700 0.42896600
H -7.13651000 -0.19213600 -1.31459000
